# The impact of parent treatment preference and other factors on recruitment: lessons learned from a paediatric epilepsy randomised controlled trial

**DOI:** 10.1186/s13063-023-07091-9

**Published:** 2023-02-06

**Authors:** Bernie Carter, Lucy Bray, Nadia al-Najjar, Agnès Tort Piella, Catrin Tudur-Smith, Catherine Spowart, Amber Collingwood, Holly Crudgington, Janet Currier, Dyfrig A. Hughes, Eifiona Wood, Rachael Martin, Christopher Morris, Deborah Roberts, Alison Rouncefield-Swales, Heather Sutherland, Victoria Watson, Georgia Cook, Luci Wiggs, Paul Gringras, Deb Pal

**Affiliations:** 1 Faculty of Health, Social Care and Medicine, Edge Hill University, Ormskirk, UK; 2 Liverpool Clinical Trials Centre, University of Liverpool, Liverpool, UK; 3 Department of Basic and Clinical Neurosciences, Institute of Psychiatry, Psychology & Neuroscience, Kings College London, London, UK; 4 Independent Consultant, London, UK; 5 Centre for Health Economics & Medicines Evaluation, Bangor University, Bangor, UK; 6 University of Exeter Medical School, University of Exeter, Exeter, UK; 7 Centre for Psychological Research, Oxford Brookes University, Oxford, UK; 8 Newcomen Children’s Neurosciences Centre, Evelina London Children’s Hospital, London, UK; 9 Department of Women and Children’s Health, Faculty of Life Sciences and Medicine, King’s College London, London, UK; 10 MRC Centre for Neurodevelopmental Disorders, King’s College London, London, UK; 11 King’s College Hospital, London, UK

**Keywords:** Parent treatment preference, Recruitment, Consent, Patient and public involvement, Randomised trial design

## Abstract

**Background:**

In paediatric epilepsy, the evidence of effectiveness of antiseizure treatment is inconclusive for some types of epilepsy. As with other paediatric clinical trials, researchers undertaking paediatric epilepsy clinical trials face a range of challenges that may compromise external validity

**Main body:**

In this paper, we critically reflect upon the factors which impacted recruitment to the pilot phase of a phase IV unblinded, randomised controlled 3×2 factorial trial examining the effectiveness of two antiseizure medications (ASMs) and a sleep behaviour intervention in children with Rolandic epilepsy. We consider the processes established to support recruitment, public and patient involvement and engagement (PPIE), site induction, our oversight of recruitment targets and figures, and the actions we took to help us understand why we failed to recruit sufficient children to continue to the substantive trial phase.

The key lessons learned were about parent preference, children’s involvement and collaboration in decision-making, potential and alternative trial designs, and elicitation of stated preferences pre-trial design.

Despite pre-funding PPIE during the trial design phase, we failed to anticipate the scale of parental treatment preference for or against antiseizure medication (ASMs) and consequent unwillingness to be randomised. Future studies should ensure more detailed and in-depth consultation to ascertain parent and/or patient preferences*.* More intense engagement with parents and children exploring their ideas about treatment preferences could, perhaps, have helped predict some recruitment issues. Infrequent seizures or screening children close to natural remission were possible explanations for non-consent. It is possible some clinicians were unintentionally unable to convey clinical equipoise influencing parental decision against participation. We wanted children to be involved in decisions about trial participation. However, despite having tailored written and video information to explain the trial to children we do not know whether these materials were viewed in each consent conversation or how much input children had towards parents’ decisions to participate. Novel methods such as parent/patient preference trials and/or discrete choice experiments may be the way forward.

**Conclusion:**

The importance of diligent consultation, the consideration of novel methods such as parent/patient preference trials and/or discrete choice experiments in studies examining the effectiveness of ASMs versus no-ASMs cannot be overemphasised even in the presence of widespread clinician equipoise.

**Supplementary Information:**

The online version contains supplementary material available at 10.1186/s13063-023-07091-9.

## Background

Randomised controlled trials (RCT) provide the best quality evidence for comparative effectiveness in medicine [1] but, historically, have been under-utilised amongst the paediatric population. Legislature and industry, including the pharmaceutical industry, now recognise that children are not small versions of adults [2, 3], but children were, and continue to be, treated with off-label treatments lacking paediatric safety and efficacy data [4]. In the field of paediatric epilepsy, there is agreement that the evidence base for antiseizure treatment overall remains of poor quality [5, 6] and antiseizure medications should be used more selectively [7].

As with other paediatric clinical trials, researchers undertaking paediatric epilepsy clinical trials face a range of challenges [8] including recruiting children to epilepsy medication effectiveness trials, the role of parent/guardian as ‘gatekeeper’ for trial participation [9–11] and factors related to consent and outcome selection and assessment. Without a robust evidence base for the best treatment option for the child, uncertainty can exist for both parents and paediatrician. In clinical trials, parent or patient preference for treatment (or non-treatment) is a common cause for recruitment and participation challenges, declining randomisation and this may compromise external validity [12, 13].

The purpose of this paper is to reflect critically upon the factors which impacted recruitment to the pilot phase of the CASTLE (Changing Agendas in Sleep, Treatment and Learning in Epilepsy) RCT examining the effectiveness of two antiseizure medications (ASMs) and a sleep behaviour intervention (details below). We were aware that issues such as randomisation, equipoise, treatment, and other aspects of and parent and/or and patient preference [11, 14, 15] were essential aspects to consider in relation to both trial design and implementation.

This paper will consider the processes which were established within the trial to support recruitment processes in the pilot CASTLE study, including our public and patient involvement and engagement (PPIE) work, the comprehensive training that underpinned site induction and our oversight of recruitment targets and figures. The paper will then report on the actions taken by the trial team to help us understand why we failed to recruit sufficient children to continue to the substantive trial phase. We reflect on the lessons learned and discuss the implications for paediatric clinical trials.

## Main text

### Trial description

CASTLE was a phase IV unblinded, randomised controlled 3×2 factorial trial comparing carbamazepine, levetiracetam or active monitoring combined with or without a sleep behaviour intervention in children (≥5 to <13 years) with Rolandic epilepsy (RE) (see Supplementary File 1: Protocol). The clinical trial was funded by the UK government’s National Institute for Health Research and registered at clinicaltrials.gov as NCT04610879. A 9-month internal pilot phase was built into the trial protocol.

### Patient and public involvement and engagement

Children with RE and parents had been involved from the inception of the idea for the CASTLE trial. In the pre-funding stage, parents were involved in two stakeholder meetings and they helped to shape the design of the study and the development of the research questions. Our engagement with parents in the pre-trial stakeholder meetings meant that we were aware that some parents of children with RE had ambivalent feelings towards medicating their children and they talked of the difficulties they experienced when trying to weigh up the potential benefits and harms of treating their child’s epilepsy with antiseizure medications. In addition, our parent co-applicants made us aware of their uncertainty about which issues to discuss with clinicians at the time of an epilepsy diagnosis and when making treatment decisions. We were clear that these ambiguities and uncertainties would need to be carefully considered in the design of the study and child/parent-facing documents.

We built on our pre-funding stakeholder work with parents when, in the early stages of the funded programme of work, we created our dedicated Patient and Public Involvement and Engagement (PPIE) Advisory Panel (AP). Our AP consists of 12 adults (including parents) with experience of childhood epilepsy, and five children with epilepsy. We recruited our AP through social media and liaison with health professionals and epilepsy charities. We worked with the AP members during face-to-face meetings and remotely (during lockdown) using open discussion facilitated by using creative methods such as road maps and idea trees and/or by email or through a dedicated WhatsApp group. The relationship and depth of consultation with the AP evolved over time and through sustained engagement.

Our AP has been influential in the programme of research related to the trial. For example, we co-created a proposed Core Outcome Set (COS) for childhood epilepsy research using a Delphi process to decide which outcomes were of key importance to stakeholders and thus the trial [16–18]. The methodology and content were informed and shaped by the priorities, experiences and preferences of children and parents from the AP panel, as well as other children with epilepsy (*n*=3), parents of children with epilepsy (*n*=16), and professionals working with children with epilepsy (*n*=61).

We specifically worked with the AP to develop the child/parent facing documentation (the trial information sheets, a short trial explainer animation) which we knew would be key to successful recruitment. Furthermore, the AP reported that the trial was quite complex for families to understand; therefore, appreciating the challenges associated with clinicians recruiting to trials we co-developed clinician-facing documentation such as a clinician guide ‘Top Tips for Recruiting Families’ sheet (Supplementary File 2) and a ‘Pictorial Trial Flow Chart’ (Supplementary File 3).

### Site induction and training

We adopted a conscientious, robust, and ongoing approach to training site research staff acknowledging that treatment preference [19] might exist.

Prior to the pilot trial launch, we invited all site investigators (clinicians and research nurses) to an in-person recruitment training event to rehearse discussions around equipoise, how to explain randomisation [20] and handling patient preference [15, 21]. Learning from the event led to the development in partnership with the AP members (children, parents), study team members, and trial recruitment experts of a portfolio of recruitment materials (e.g. consent training videos, cartoons, and materials) designed to aid understanding of the study by both investigators and families. Additional face-to-face site-specific induction training covered all the details about the trial and included informed consent, issues of randomisation, equipoise, and patient preference. Open dialogue was encouraged to ensure any queries or concerns relating to the CASTLE trial were discussed and clarified.

### Recruitment targets, issues with recruitment and consent

As a key part of quality control, throughout the internal pilot period of the trial, the CASTLE Trial Management Group performed monthly review of the trial screening data by site. The internal pilot trial aimed to assess recruitment and consent during the first 9 months of recruitment. We were guided by clear recruitment progression criteria (see Table 1) and, in addition, we had a target to open 22 sites.

**Table 1:** Recruitment targets

Recruitment % of planned number of participants (***n***=30)	Expected outcome/action
Recruitment 80–100% (*n*=28)	Trial would progress
Recruitment 50–79%	Trial would progress (following review of screening logs and protocol and once barriers to achieve adequate recruitment were addressed)
Recruitment <50%	Trial not expected to progress

**Fig. 1:**
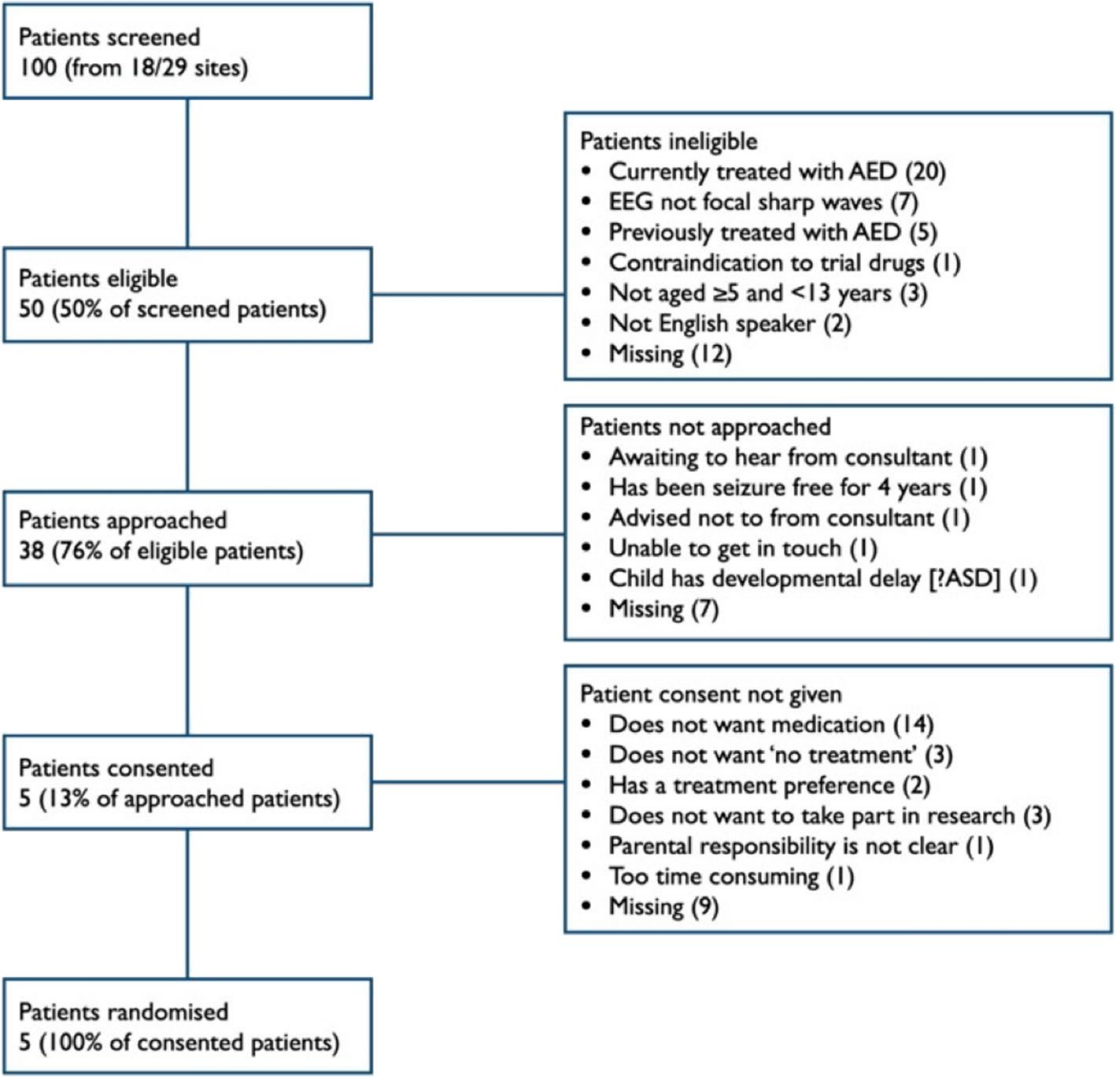
Internal pilot screening and recruitment summary

**Fig. 2:**
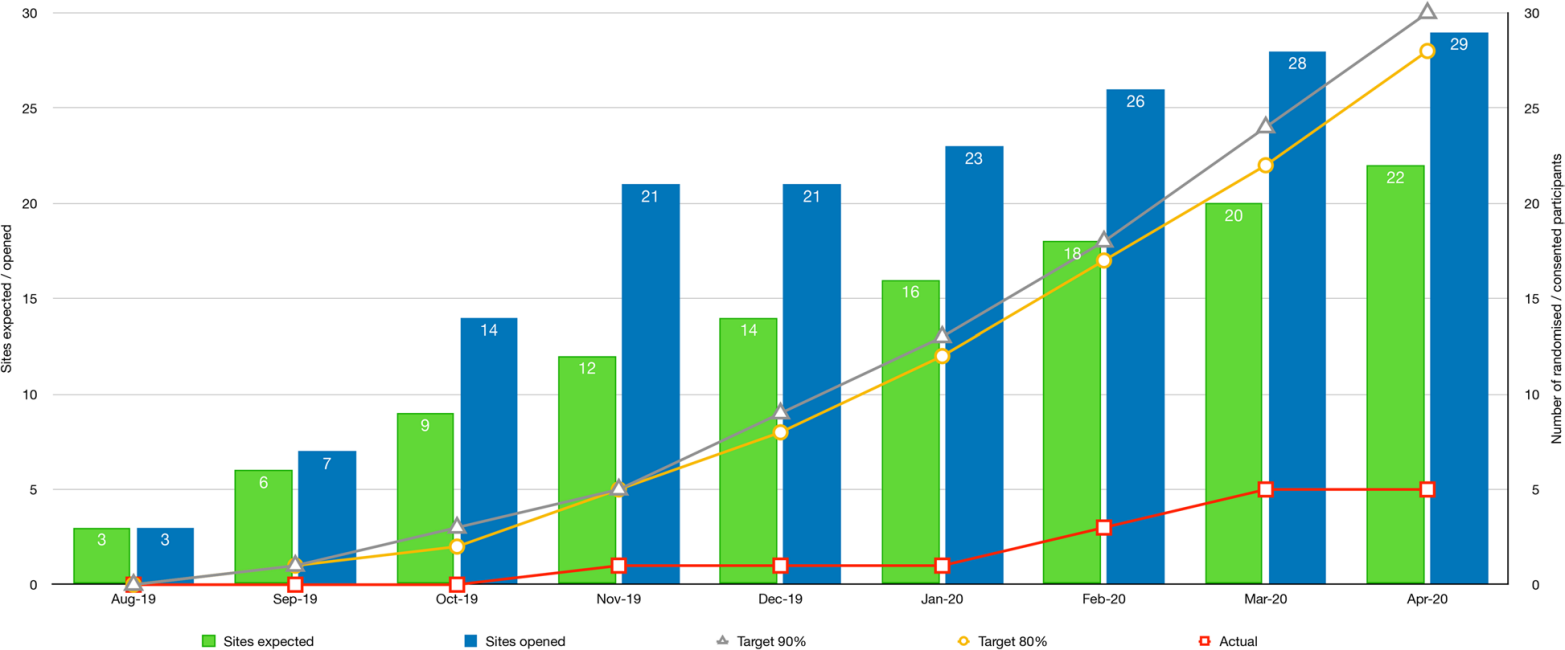
Recruitment against original target in CASTLE pilot

We were successful in site initiation; 31 sites were trained, and 29 trial sites were opened (seven more than our goal), most in secondary care paediatric centres. However, of these 29 sites, only 18 had screened at least one patient by month nine of the internal pilot. In total, 100 patients were screened (see Figs. 1 and 2): of these 100, 50 patients (50%) met the trial eligibility criteria. Thirty-eight of 50 eligible patients (76%) were approached for consent to the trial: only five (13%) consented to be randomised. One of the exclusion criteria — currently or previously treated with ASMs — accounted for 52% (*n*=26) of ineligible patients. Both eligibility and approach rates were within our expectations; however, the consent rate was considerably lower than anticipated.

Non-consent for 19 of the 50 eligible patients (38%) was due to some parental preference about treatment: 14 did not want medication, three did not want ‘no treatment’, and two had a ‘treatment preference’ (one against carbamazepine, another in favour of any medication). One patient was too far along their natural course towards remission (seizure free for 4 years). Of the remaining 14 approached, three did not want to take part in research, one said it would be too time-consuming, parental responsibility was not clear for one family and the remaining nine did not provide a reason.

## Actions taken by the trial team to understand the reasons for low recruitment

As it became clear that the consent rate was poor and not improving, it was apparent we needed to understand the factors impacting on the recruitment rate. To this end, we consulted and engaged with our two key stakeholder groups: health professionals involved with recruitment and parents and children from our AP.

### Consultation with health professionals undertaking recruitment

In recognition of our recruitment challenges, we consulted paediatricians and research nurses responsible for recruitment during the internal pilot to engage in an exercise to understand their perceptions and experiences of recruitment to the trial. Having utilised the HRA decision tool (http://www.hra-decisiontools.org.uk/research/) it was clear we did not need ethics review to conduct the interviews. The aim of the consultation was to ascertain any difficulties regarding eligibility, reasons identified for patients/parents declining participation, and health professionals’ confidence in explaining the trial to families. Three trial team members (BC, AR-S, LB) contributed to the collection of information via telephone interview or email using agreed key questions to ensure core issues were discussed with each of the health professionals. The findings from the consultation were summarised and anonymous quotes are used to illustrate specific points.

Despite the disruption to services resulting from the COVID-19 pandemic, 12 clinicians and two research nurses from 14 of the 29 sites shared their views (17th March–15th April 2020) as part of the consultation; seven clinicians and two research nurses were consulted via telephone and five clinicians responded to questions by email. A further five sites acknowledged our request but were unable to provide responses to our consultation as they had neither approached nor recruited families to participate in the trial. Of the 14 sites who engaged in the consultation, four had recruited a child/family, seven sites had experience of families declining to participate (*n*=23 families), three sites had families they had approached but who had not yet decided, and three sites had not identified any eligible families.

Overall, clinicians were positive about the CASTLE trial considering it to be “*a win-win situation*” for families. Clinicians demonstrated a sense of clinical equipoise acknowledging that both arms of the study (medication and sleep) were important and were willing to “*emphasise that point when discussing with families*”*.* Several clinicians worked “*as a team*” with their research nurses to effectively screen, explain and recruit potential families to the trial. The clinicians felt the trial team had provided adequate guidance and resources to help them explain the trial to families such as our ‘Top Tips for Recruiting Families’ sheet (Supplementary File 2) and a ‘Pictorial Trial Flow Chart’ (Supplementary File 3). All the clinicians were confident about talking about all aspects of randomisation and trial design with families.

However, a significant difficulty for individual sites was identifying potential participants who met the eligibility criteria. Some clinicians explained that fewer patients than expected (pre-trial commencement) met the trial eligibility criteria, typically noting that since the study opened eligible patients were simply “*not there*”. Most commonly, clinicians said that potential participants were not drug naïve and therefore ineligible to take part.

The families were reported as giving the clinicians various reasons for declining to participate in the CASTLE trial. Most frequently, the concept of randomisation in the CASTLE trial was reported as causing some unease amongst families where parents had commented that their child’s seizures were “*not bad enough yet*” to warrant medication. Clinicians also reported some parents had emotional responses to randomisation, which has been identified as complicating factors in recruitment in other trials [22]. One of the clinicians noted that unease over randomisation was sustained despite having had “*extensive conversations*” and further noting that “*the points they made [were] valid*”; perhaps suggesting that, for this clinician conveying equipoise may have been challenging [23], did not exist or at least was in tension as suggested in work which contests the value of equipoise [24]. More unusually, a clinician explained that one family had chosen not to participate due to a strong preference for carbamazepine and did not want to risk randomisation. For one family, the parents’ sense of urgency regarding commencing medication was a key reason for them declining to participate; the clinician explained that “*nothing I said in clinic gave me an opportunity to change that around…. They wanted the treatment ASAP – they didn’t want to wait till the following week when they would get recruited*”*.*

However, despite the above challenges some initially reluctant families changed their minds about participating due to changing circumstances. One family was reported to have changed their mind after their child started “*having more frequent fits*” and felt more accepting of being randomised to medication as this new context mean that “*it would be OK*”*.* Another family when told that their child’s ‘sleep issues’ were most likely due to night-time seizures were reported to have changed their mind and agreed to be randomised “*because they were expecting that the fits would be [only] during the day*”.

The clinicians reported that most of the families were interested in the sleep intervention and noted that “*it would be much easier to recruit if the two interventions [medication and sleep] were evaluated separately*”*.*

In summary, consultation with clinicians highlighted that some parents expressed strong medication related preferences; this meant randomisation to a potentially non-preferred arm discouraged parents. However, clinicians considered that the parent treatment preference issue was less likely to impact on recruitment to a trial that only involved randomisation to either sleep intervention plus usual care or usual care only.

### Engagement with our PPIE advisory panel (AP)

Although we had engaged with stakeholders from the inception of the study, our full AP was not established until after some of the key trial design decisions (e.g. research questions, interventions and design) had been taken.

As recruitment and consent challenges became evident, we dedicated time in February 2020 to discuss this at an AP meeting, with seven parents present. They discussed and expressed a range of opinions about how parent preference for treatment/non-treatment was likely to be an important factor influencing trial participation. One parent proposed that medicine randomisation “*could be off putting as at that point [diagnosis] you can feel quite desperate and out of control and it is important to have that choice*”. Another explained that the need to be certain that their child would be prescribed medication was the overriding factor, explaining “*at the start everything is so uncertain, and you think that the medicine will be a magic cure and so of course you would want that*”. Another parent expressed a reluctance for their child to be prescribed medication and explained that “*we felt that we did not want medicines due to possible side effects*”. These concerns seemed to resonate with ones that the recruiting clinicians were describing. The views of the AP alongside the clinician reports were discussed at length within the Trial Management Group (TMG) and informed the discussions about revising the trial design.

The TMG requested further input from the AP about their perspectives on the potential of an alternative design proposing a Patient Preference Trial (PPT) design focusing solely on the sleep behaviour intervention but without the comparison between carbamazepine and levetiracetam. A remote AP meeting was held with 11 parents and five children to explore their views on the proposed PPT design and ensure their expert feedback informed any proposed design. All the parents unanimously reported that the proposed PPT design was more acceptable to them than the original trial design as it was “*less like taking a lottery with my child’s treatment*”, gave “*families the choice for medication or no medication*” and empowered parents to have “*some control over something*”.

The increased focus on sleep in the proposed PPT trial design was deemed important with parents highlighting how “*the sleep part for us is so important*” and “*sleep is a huge thing for us*”. Some children in the AP had struggled with their sleep for many years and talked about the lack of sleep “*being really hard and affects me in school*”. The proposed trial design was described by children in the AP as “*simpler and makes more sense to us*” and parents thought it would result in “*more recruitment and more sign up from families*”.

## Discussion of the lessons we learned

The discussion that follows addresses the key lessons we learned about parent preference, children’s collaboration in decision-making to participate, alternative trial designs and elicitation of stated preferences pre-trial design. Each of these is critically considered before drawing final conclusions.

### Parent preference

Parent preference, particularly treatment preference, is an important factor across paediatric RCTs, not least because of the proxy role that parents often play in making treatment decisions [11, 25, 26]; this was evident in this pilot trial with parents of children aged ≥5 to <13 years (at time of randomisation).

Treatment preference is a key influence which has been found to lead to declining participation in up to 70% of a variety of 52 trial designs [27]. We had some prior indication of treatment ambivalence in the pre-trial planning stakeholder meetings, but we had failed to anticipate its translation to such strong parent treatment preference. Clinicians reported that infrequent seizures or screening children already close to natural remission (which occurs at a median of two to three years after seizure onset) were possible explanations for non-consent, as parents did not want to be at risk of their child being randomised to start medication. Another explanation may be that some clinicians were unintentionally unable to convey clinical equipoise and influenced parental decision-making against participation. It is important to note that even if clinical equipoise exists and is conveyed this does not necessarily make parents’ decision-making any easier.

Post hoc trial surveys suggest that parents with higher socio-economic status, high decisional uncertainty and low levels of trust and altruism were more likely to decline trial participation [25], although most of these factors are not modifiable, there are opportunities to reduce decisional uncertainty. Qualitative research shows that, when deciding about trial entry, parents consider clinical benefit, child safety, practicalities of participation, research for the common good, access to medication and randomisation [11]. Additionally, specific misunderstandings have the potential to influence parents’ decisions, but parents rarely voice concerns during discussions with practitioners [11]. Other research has shown that parental reasons for strongly held preferences include concerns about adverse effects and negative attitudes towards ‘new’ or ‘experimental’ interventions [27]. We hypothesise that misgivings about ASMs represent some of the unvoiced concerns in the CASTLE trial. Anecdotally, some parents approached for the pilot, and who declined participation, disclosed medicating their children with over-the-counter cannabidiol products, hinting at a distinction between these products and conventional ASMs.

### Children’s collaboration in decision-making to participate

Children’s ability to participate in research is vulnerable to adult proxies. Considering this, we started from the premise that children want to collaborate in decision-making about participating in medical research [28], and that investigators must aim to involve children in discussions about research and obtain their assent to participation [9, 10]. We were aware that child preference is frequently unreported and sometimes differs to their parents’ preferences [15, 26]. Although we did not identify issues with children declining, we propose that earlier and more intense engagement with children exploring their ideas about treatment preferences could, perhaps, have helped us predict some of the recruitment issues. So, although we had developed tailored information (Supplementary Files 4 and 5) and video materials (co-designed by our AP) to explain the trial to children (aged 7–12 years) we do not know whether these materials were viewed in each consent conversation or how much input children had towards parents’ decisions to participate.

Our original site training reflected our awareness that bespoke training of people undertaking trial recruitment of children and parents has been shown to improve clarity and balance of explanations and increase recruitment [29]. Our future training of recruiting clinicians further emphasises the importance of engaging with children about the research, whilst acknowledging parents have the legal authority to determine a child’s participation.

### Consideration of alternative trial designs

With failure to recruit to our randomised controlled factorial trial, we needed to consider how to take our programme of work forward. Potentially core to our failure to recruit is the fact that ASMs do not generally modify the natural history of epilepsy but aim to prevent the occurrence of seizures and consequent harm. There has been only one placebo-controlled paediatric RCT of ASMs in RE [30] and, because of their now-established place in the management of epilepsy, such a comparison would be considered unethical in most countries. However, in the minds of UK clinicians, RE (the focus of our trial) remains an exception to this principle because of its self-limiting nature and perfectly illustrates the uncertainty about the risk-benefit equation of ASMs in childhood. Hence, we believe it is still important to address the ASMs /no-ASMs question in a design more acceptable to parents and children. Whilst changing the randomisation arms is one obvious option to make the trial design more acceptable to potential participant families, incorporating parent and patient preferences is another increasingly used adaptation [27]. PPTs allow patients to choose their preferred intervention arm but at the expense of increased sample size and trial duration; such designs do not necessarily improve informed consent though [27]. Although a PPT design was offered as an option following the pilot phase and was acceptable to parents and children on our AP, a simplified trial design evaluating the behavioural sleep intervention vs standard care, with no ASMs arms was preferred by the trial funder.

### Elicitation of stated preferences pre-trial design

A key lesson was that despite our pre-funding stakeholder engagement, we had failed to anticipate the scale of parental treatment preference against antiseizure medication (ASMs) randomisation. In future work, this should be considered. More detailed and robust work to ascertain patient preferences earlier in the trial design process might have identified likely barriers to recruitment and informed the design of the RCT at an earlier stage.

As it became clear that low recruitment meant that the original trial design could not proceed, the consultation with clinicians and families conducted led us to recognise that trial designs with PPT elements were favoured by the clinicians, parents, and children with epilepsy. We propose that discrete choice experiments (DCEs) have potential in this regard. DCEs can elicit patients’ stated preferences for health technologies, interventions, and services [31, 32]. Discrete choice approaches are rooted in random utility theory [33] and underpinned by a view of utility which contends that goods and services (ASMs in this case) can be described by their characteristics or attributes and that the utility (satisfaction) yielded by ASMs is a function of their various attributes. The total utility a child/parent experiences in the use and non-use of ASMs is a function of the combinations of these attributes. Rational choice theory contends that children/parents would choose scenarios that maximise their utility. Previous DCE approaches have included eliciting parent preferences on behalf of children [34], and whilst some DCEs assessing preferences for drug treatments in the management of epilepsy have been conducted [35–37], to our knowledge none included paediatric populations.

The design of a DCE requires formative work to identify suitable attributes and attribute levels [38]. The DCE would ideally need to be administered in a separate cohort, as there may be a risk that undertaking the experiment in trial participants (or their parents) may influence their decision to consent to the RCT. The results of the DCE would provide an understanding of the willingness of respondents to trade seizure control for fewer side-effects, or choice of treatment for disease severity, and could have signalled that a trial of ASMs versus no treatment would not be feasible based on the strength of respondents’ stated preferences, expressed as their utility [36, 39]. Alternatively, a conventional parallel arm trial could be adapted to accommodate a priori preferences.

### Limitations

The lessons we have learned are contextual and are based on consultation with a small number of health professionals and the parents and children in our AP and not on direct engagement with children (and their parents) who were eligible to participate in the trial. This limits the representativeness of our consultation and the extent to which the reasons for declining participation can be unravelled.

## Conclusions

Paediatric epilepsy poses particular challenges for clinical trial design, planning and execution, frequently resulting from discordance of perception, understanding and sharing of information between parents, children and clinicians regarding both the impact of seizures and potential benefits and harms of ASMs. The importance of careful consultation and planning of novel interventions and intervention trials cannot be overplayed even in the presence of widespread clinician equipoise.

## Supplementary Information

**Additional file 1.** CASTLE Protocol v3. Trial protocol for CASTLE.

**Additional file 2.** Top Tips for Recruiting Families. Top Tips for Recruiting Families-original CASTLE Trial.

**Additional file 3.** Pictorial Trial Flow Chart. Pictorial Trial Flow Chart-original CASTLE trial.

**Additional file 4.** Information Sheet 5-6yrs. Information Sheet for Children 5-6yrs-revised trial.

**Additional file 5.** Information Sheet 7-12yrs. Information Sheet etc for Children 7-12yrs-revised trial.

## Data Availability

The CASTLE trial was terminated after the pilot. The datasets used and/or analysed during the CASTLE pilot as well as select trial materials are available from the corresponding author (DP) on reasonable request (see also pages 53-54 of the attached protocol). The information from the consultation with the health professionals is not available as this is not research data.

## Abbreviations

ASM: Antiseizure medication
AP: Advisory Panel
CASTLE: Changing Agendas in Sleep, Treatment and Learning in Epilepsy
COS: Core outcome set
DCE: Discrete choice experiment
PPIE: Public and patient involvement and engagement
PPT: Patient preference trial
RCT: Randomised controlled trial
RE: Rolandic epilepsy
