# Supplementary material for: The impact of parent treatment preference and other factors on recruitment: lessons learned from a paediatric epilepsy randomised controlled trial
Source: Trials. 2023 Feb 6;24:83. doi: 10.1186/s13063-023-07091-9 (PMC9900533; doi:10.1186/s13063-023-07091-9)
Supplement: Supplementary file 1 — Additional file 1. CASTLE Protocol v3. Trial protocol for CASTLE. [file 13063_2023_7091_MOESM1_ESM.pdf]

Randomised factorial design controlled trial comparing carbamazepine, levetiracetam or active monitoring combined with or without sleep behaviour intervention in treatment naive children with rolandic epilepsy

CASTLE Protocol v3.0 18/08/2020

Trial Co-Sponsors:

King's College Hospital NHS Foundation Trust
Research and Innovation
Denmark Hill
London, SE5 9RS
Tel: 020 3299 1985

King's College London
James Clerk Maxwell Building
57 Waterloo Road
London SE1 8WA
Tel: 0207 848 6391

EudraCT number: 2018-003893-29

IRAS Number: 250324

ISRCTN Number: ISRCTN12839803

Funder reference: RP-PG-0615-20007

PROTOCOL APPROVAL

I, the undersigned, hereby approve this clinical trial protocol:

Authorised by Chief Investigator:

Signature: _____________________________________ **Date:** __________

Professor Deb K Pal
Professor of Paediatric Epilepsy, King's College London

Authorised on behalf of the Lead Statistician:

Signature: _____________________________________ **Date:** ___________

Professor Catrin Tudur Smith
Professor of Biostatistics
CTRC, University of Liverpool

General Information

This document describes the CASTLE trial including detailed information about procedures and recruitment. The protocol should not be used as an aide-memoir or guide for the treatment of other patients; every care was taken in its drafting, but corrections or amendments may be necessary. These will be circulated to the registered investigators in the trial, but centres entering patients for the first time are advised to contact the coordinating centre (CTRC) to confirm they have the most up to date version. Clinical problems relating to this trial should be referred to the Chief Investigator, Professor Deb Pal, via the CTRC.

This protocol defines the participant characteristics required for trial entry and the schedule of treatment and follow-up. Participant recruitment will be undertaken in compliance with this document and applicable regulatory and governance requirements; waivers to authorise non-compliance are not permitted.

Incidence of protocol non-compliance, whether reported prospectively (e.g. where a treatment cannot be administered on a scheduled date as a result of public holidays) or retrospectively noted (e.g. as a result of central monitoring) are recorded as protocol deviations, the incidence of which is monitored and reported to trial oversight committees.

The template content structure is consistent with the SPIRIT (Standard Protocol Item: Recommendations for Interventional Trials 2013) and has regard for the Health Research Authority guidance. Regulatory and ethical compliance information is located in section 12.

Relationship Statements

Roles and responsibilities are fully described in section 15.

The co-sponsors, King's College Hospital NHS Foundation Trust and King's College London, will formally delegate specific sponsoring roles to the King's Health Partners Clinical Trial Office (KHP CTO), Chief Investigator and Clinical Trials Unit, but remain legally responsible for the trial.

The KHP CTO Quality Team was established in 2008 to manage the Sponsor responsibilities for Clinical Trials of Medicinal Products (CTIMPs), as defined in the Regulations, for trials sponsored or co-sponsored by King's Health Partner Organisations. The KHP CTO also assists Chief Investigators with the set up and initiation of their trial and to ensure that these trials are conducted according to GCP and the Regulations, throughout the life of the trial.

Clinical Trials Unit: The CTRC at the University of Liverpool in collaboration with the Chief Investigator, Professor Deb Pal, will have overall management responsibility for the trial and will be responsible for the co-ordination of centres.

CTRC, as part of the Liverpool Clinical Trials Collaborative, has achieved full registration by the UK Clinical Research Collaboration (www.ukcrc.org) as their standards and systems were assessed by an international review panel as reaching the highest quality. CTRC has a diverse trial portfolio underpinned by methodological rigour, a GCP compliant data management system, and core standard operating procedures.

Contact Details: Institutions

**Co-Sponsors:**	**Trial Management, Monitoring and Analysis:**	**Sleep Intervention:**
King's College Hospital NHS Foundation Trust<br><br>Research and Innovation<br>Denmark Hill<br>London<br>SE5 9RS<br>Tel: 020 3299 1985<br><br>King's College London<br><br>James Clerk Maxwell Building<br>57 Waterloo Road<br>London SE1 8WA<br>Tel: 0207 848 6391	Clinical Trials Research Centre<br><br>University of Liverpool<br>Institute in the Park, 2nd Floor<br>Alder Hey Children's Hospital<br>Eaton Road<br>Liverpool<br>L12 2AP<br>Tel: 0151 795 8774<br><br>Email: castle.rct@liverpool.ac.uk	Oxford Brookes University<br><br>Department of Psychology, Health and Professional Development<br>Oxford Brookes University<br>Headington Campus<br>Oxford<br>OX3 0BP
**Qualitative Study:**	**Health Economics:**	
Edge Hill University<br><br>Faculty of Health and Social Care<br>Edge Hill University<br>St Helen's Road<br>Lancashire<br>L39 4QP	Bangor University<br><br>Centre for Health Economics and Medicines Evaluation<br>Bangor University<br>Holyhead Road<br>Bangor<br>Gwynedd<br>North Wales<br>LL57 2PZ	

Contact Details: Individuals

**Management of Co-Sponsor Responsibilities:**	**Chief Investigator (CI)**
**Ms Amy Holton**<br>Quality Manager<br>King's Health Partner's Clinical Trials Office<br>F16, Tower Wing<br>Guy's Hospital<br>Great Maze Pond<br>London<br>SE1 9RT<br>Tel: 0207 188 5732<br>Email: amy.holton@kcl.ac.uk	**Professor Deb Pal**<br>Maurice Wohl Clinical Neuroscience Institute<br>King's College London<br>5 Cutcombe Road<br>London<br>SE5 9RX<br>Tel: 0207 848 5762<br>Email: deb.pal@kcl.ac.uk
**Co-Investigator who will Advise on Protocol Related Clinical Queries in absence of CI**	**Medical Experts who will Advise on Protocol Related Clinical Queries and who will Evaluate SAE Reports (If other than CI/Co-Investigator)**
**Professor Paul Gringras**<br>Consultant in Paediatric Sleep and Neurodisability<br>Evelina London Children's Sleep Medicine Department<br>Children's Neurosciences<br>St Thomas' Hospital<br>Westminster Bridge Road<br>London<br>SE1 7EH<br>Tel: 020 7188 4649<br>Email: Paul.gringras@gstt.nhs.uk	**Dr Frances Gibbon**<br>Noah's Ark Children's Hospital for Wales<br>Cardiff and Vale University Health Board<br>Cardiff<br>CF14 4XW<br>Tel: 029 2184 7310<br>Email: frances.gibbon@nhs.net<br><br>**Dr Colin Dunkley**<br>Sherwood Forest Hospitals NHS Foundation Trust<br>Mansfield Road<br>Sutton in Ashfield<br>NG17 4JL<br>Tel: 01623 672383<br>Email: Colin.Dunkley@sfh-tr.nhs.uk
**Statistics Lead**	**Health Economics Lead**
**Professor Catrin Tudur Smith**<br>Professor of Biostatistics<br>Department of Biostatistics<br>Clinical Trials Research Centre<br>University of Liverpool<br>Block F, Waterhouse Building<br>1-5 Brownlow Street<br>Liverpool<br>L69 3GL<br>Email: cat1@liv.ac.uk	**Professor Dyfrig Hughes**<br>Centre for Health Economics and Medicines Evaluation<br>Bangor University<br>Holyhead Road<br>Bangor<br>Gwynedd<br>North Wales<br>LL57 2PZ<br>Tel: 01248 382950<br>Email: d.a.hughes@bangor.ac.uk

Sleep Intervention Lead	Qualitative Research Lead
**Dr Luci Wiggs**<br>Reader in Psychology<br>Department of Psychology<br>Health and Professional Development<br>Faculty of Health and Life Sciences<br>Oxford Brookes University<br>Gipsy Lane<br>Headington<br>Oxford<br>OX3 0BP<br>Email: lwiggs@brookes.ac.uk	**Professor Bernie Carter**<br>Faculty of Health and Social Care<br>H217a<br>FOHSC Building<br>Edge Hill University<br>St Helens Road<br>Ormskirk<br>Lancashire<br>L39 4QP<br>Tel: 01695 657771<br>Email: bernie.carter@edgehill.ac.uk

Additional Contacts:
The contact details of other individuals involved in the trial are detailed in documents supplementary to the protocol and stored in the Trial Master File:

Contact	Document Title
**Trial Management Group**<br><br>**Trial Steering Committee (TSC)**<br><br>**Independent Data and Safety Monitoring Committee (IDSMC)**	CASTLE Trial Oversight Committee Membership

1 TABLE OF CONTENTS

Protocol Approval 2
1 TABLE OF CONTENTS 7
Glossary 10
2 PROTOCOL SUMMARY 12
2.1 Sub-study Summary: 15
3 INTRODUCTION 17
3.1 Background 17
3.1.1 Rolandic Epilepsy 17
3.1.2 The Clinical Trial 17
3.2 Rationale 17
3.2.1 Risk and Benefits 19
3.2.2 Known Potential Benefits 19
3.3 Objectives and Outcome Measures/Endpoints 19
4 TRIAL DESIGN 22
5 TRIAL SETTING, SELECTION OF CENTRES/CLINICIANS 23
5.1 Selection of Centres/Clinicians 23
6 TRIAL POPULATION 24
6.1 Inclusion Criteria 24
6.2 Exclusion Criteria 24
6.3 Sub-study Inclusion Criteria 24
6.4 Co-enrolment Guidelines 24
7 RECRUITMENT AND RANDOMISATION 25
7.1 Participant Identification and Screening 25
7.2 Informed Consent 25
7.2.1 Consent procedures 25
7.2.2 Parental Responsibility; Who is eligible to provide proxy consent? 26
7.2.3 Assent of minors 26
7.2.4 Consent for the qualitative sub-study 26
7.2.5 Re-consent of minors who reach 16 years during trial participation 27
7.3 Where consent to participate in CASTLE is declined 27
7.4 Enrolment/Baseline 27
7.5 Randomisation Procedures 27
7.5.1 Back up Randomisation Process 28
7.5.2 Contacts Database 28
7.5.3 Qualitative Sub-Study Identifiers 29
7.6 Who is Blinded to Allocations 29
8 PARTICIPANT TIME LINE, ASSESSMENTS AND PROCEDURES 30
8.1 Participant timeline 30
8.1.1 Schedule of Trial Procedures by Visit 30
8.1.2 Schedule for Follow-up 34
8.2 Procedures for assessing Efficacy 34
8.2.1 Seizure outcomes 34
8.2.2 Health Related Quality of Life 35
8.2.3 Strengths and Difficulties Questionnaire 35
8.2.4 Parent self-efficacy measure 35
8.2.5 Sleep 35
8.2.6 Learning 36
8.3 Procedures for Assessing Safety 36

8.4 Other Assessments 36
8.4.1 Qualitative interviews 36
8.4.2 Health Economics 37
8.4.3 End of trial procedures 38
8.5 Participant Transfer and Withdrawal 38
8.5.1 Participant Transfers 38
8.5.2 Stopping the Allocated Trial Intervention 38
8.5.3 Withdrawal from Trial Completely 39
8.6 Loss to Follow-up 39
8.7 Trial Closure 39

9 TRIAL TREATMENT/INTERVENTIONS 40
9.1 Interventions 40
9.1.1 Randomised Drug Interventions or No Drug 40
9.1.2 Randomised Sleep Intervention 40
9.2 Additional Considerations for Drug Interventions 41
9.2.1 Formulation, Packaging, Labelling, Storage and Stability 41
9.2.2 Preparation, Dosage and Administration of Trial Treatment/s 42
9.2.3 Dose Modifications and Management of Toxicity 42
9.2.4 Drug Accountability and Assessment of Compliance 43
9.2.5 Precautions Required for Trial Treatments 43
9.2.6 Concomitant Medications 43
9.2.7 Overdose 43

10 SAFETY REPORTING 44
10.1 Terms and Definitions 44
10.2 Notes on Adverse Event Inclusions and Exclusions 45
10.2.1 Inclusion of Related Adverse Events (Adverse Reactions) 45
10.2.2 Exclusions 45
10.2.3 Notification of Deaths 46
10.2.4 Reporting of Pregnancy 46
10.2.5 Reporting of Overdoses 46
10.3 Notes on Severity / Grading of Adverse Events 46
10.4 Relationship to Trial Treatment 47
10.5 Expectedness 47
10.6 Follow-up After Adverse Reactions 47
10.7 Reporting Procedures 48
10.7.1 Non-serious and unrelated AEs 48
10.7.2 Non-serious ARs 48
10.7.3 Serious safety events (SAEs/SARs/SUSARs) 48
10.8 Responsibilities – Investigator 49
10.9 Responsibilities – CTRC and KHP CTO 50
10.9.1 Safety reports 51
10.9.2 Urgent Safety Measures (USMs) 51
10.10 Contact Details and Out-of-hours Medical Cover 52

11 STATISTICAL CONSIDERATIONS 53
11.1 Introduction 53
11.2 Method of Randomisation 53
11.3 Sample Size calculation 53
11.3.1 Drug Intervention 53
11.3.2 Sleep Intervention 53
11.3.3 Feasibility (attaining recruitment targets) 53
11.4 Interim Monitoring and Analyses 53
11.5 Analysis Plan 54
11.5.1 Primary and Secondary Outcomes 54
11.5.2 Health Economic Evaluation 55

12 REGULATORY AND ETHICAL APPROVALS 57
12.1 Statement of Compliance 57
12.2 UK Policy Framework for Health and Social Care Research Regulatory Approval 57
12.3 Ethical Considerations 57
12.4 Ethical Approval 57
12.5 Protocol Deviation and Serious Breaches 57
12.6 Trial Discontinuation 58
13 DATA MANAGEMENT AND TRIAL MONITORING 59
13.1 Risk Assessment 59
13.2 Source Documents 59
13.3 Data Capture Methods 60
13.4 Monitoring 60
13.4.1 Central Monitoring 60
13.4.2 Clinical Centre Monitoring 61
13.5 Confidentiality 61
13.6 Quality Assurance (QA) and Control (QC) 62
13.7 Records Retention 62
14 INDEMNITY 64
15 ROLES AND RESPONSIBILITIES 65
15.1 Role of Trial Co-Sponsors and Trial Funder 65
15.2 Funding and Support in Kind 65
15.3 Protocol Contributors 65
15.4 Trial Committees 66
15.4.1 Trial Management Group (TMG) 66
15.4.2 Trial Steering Committee (TSC) 66
15.4.3 Independent Data and Safety Monitoring Committee (IDSMC) 66
16 PUBLICATION AND DISSEMINATION 67
16.1 Publication Policy 67
Authorship 67
16.2 Dissemination to Key Stakeholders 67
16.3 Data Sharing 67
17 CHRONOLOGY OF PROTOCOL AMENDMENTS 68
17.1 Version 1.0 (20/02/2019) 68
17.2 Version 2.0 (24/05/2019) 68
17.3 Version 3.0 (18/08/2020) 69
18 REFERENCES 70

Table 1: Summary of Objectives, Outcome Measures and Timepoints 19
Table 2: Schedule of Trial Procedures 33
Table 3: Schedule of Sub-Study Interviews 34
Table 4: Definitions of Causality 47

Figure 1: Schematic of Trial Design 16
Figure 2: Flowchart for Reporting Requirements of Safety Events 49

GLOSSARY

ABPI	Association of the British Pharmaceutical Industry
AE	Adverse Event
AED	Anti-Epileptic Drug
AR	Adverse Reaction
A&E	Accident & Emergency
BECTS	Benign Epilepsy with Centrotemporal Spikes
CBZ	Carbamazepine
CHEQOL	Health Related Quality Of Life In Children With Epilepsy
CI	Chief Investigator
CRF	Case Report Form
CSHQ	Children's Sleep Habits Questionnaire
CTRC	Clinical Trials Research Centre
EEG	Electroencephalography
GDPR	General Data Protection Regulation
GP	General Practitioner
HEAP	Health Economic Analysis Plan
HES	Hospital Episode Statistics
HRA	Health Research Authority
HRQL	Health Related Quality of Life
HTA	Health Technology Assessment
ICO	Information Commissioners Office
IDSMC	Independent Data and Safety Monitoring Committee
IEC	Independent Ethical Committee
ILAE	International League Against Epilepsy
IPD	Individual Participant Data
ISF	Investigator Site File
ITT	Intention To Treat
KHP CTO	King's Health Partners Clinical Trials Office
LEV	Levetiracetam
MHRA	Medicines and Health Care Products Regulatory Agency
NICE	National Institute for Health and Care Excellence
NIHR CRN	National Institute for Health Research Clinical Research Network
NRES	National Research Ethics Service
NRLS	National Reporting and Learning System
PBS	Parent-Based Sleep Intervention
PI	Principal Investigator
PIC	Participant Identification Centre
PISC	Patient Information Sheet and Consent
PLICS	Patient-Level Information and Costing Systems
PPI	Patient and Public Involvement
PSAM	Parenting Self-Agency Measure
PSS	Personal Social Services
QA	Quality Assurance

QALYs	Quality-Adjusted Life-Years
QC	Quality Control
QOL	Quality of Life
RE	Rolandic Epilepsy
R&D	Research & Development
RCT	Randomised Controlled Trial
REC	Research Ethics Committee
RSI	Reference Safety Information
SAE	Serious Adverse Event
SAP	Statistical Analysis Plan
SAR	Serious Adverse Reaction
SDQ	Strengths and Difficulties Questionnaire
SDV	Source Data Verification
SmPC	Summary of Product Characteristics
SOP	Standard Operating Procedure
SUSAR	Suspected Unexpected Serious Adverse Reactions
TMG	Trial Management Group
TSC	Trial Steering Committee
UAR	Unexpected Adverse Reaction
USM	Urgent Safety Measure

2 PROTOCOL SUMMARY

**Full Title:**	Randomised factorial design controlled trial comparing carbamazepine, levetiracetam or active monitoring combined with or without sleep behaviour intervention in treatment naive children with rolandic epilepsy
**Acronym:**	CASTLE
**Phase:**	IV
**Target Condition:**	Treatment naive children with rolandic epilepsy (RE) in the UK.
**Sample size:**	330
**Main Inclusion Criteria :**	a. Children diagnosed with RE<br>b. EEG showing focal sharp waves with normal background<br>c. Aged ≥5 years and <13 years at time of randomisation<br>d. Currently untreated with antiepileptic drugs<br>e. Written informed consent received from person with parental responsibility/legal representative<br>f. Family have a valid email address and regular internet access (for online sleep intervention)<br>g. Parent and child are to have a good understanding of the English language
**Main Exclusion Criteria :**	a. Known contraindication to any trial drugs<br>b. Previously treated for epilepsy with antiepileptic drugs
**Trial Centres and Distribution:**	UK secondary or tertiary centres for paediatrics.
**Participant Trial Duration:**	Minimum/Maximum 12/48 months
**Overall Trial duration:**	60 months
**Agent/ Interventions:**	**Interventions:**<br>(1) Oral carbamazepine or levetiracetam prescribed in a formulation and at a dose deemed suitable by the treating physicians and guided by ranges in summary of product characteristics (SmPC).<br>(2) Sleep behaviour intervention<br><br>**Controls:**<br>(1) No antiepileptic drug treatment (AED) – active monitoring<br>(2) No sleep behaviour intervention – standard care

	Objectives	Outcome Measures
**Primary:**	To determine if carbamazepine or levetiracetam are superior to no AED with respect to time to 6- month seizure remission.	Time to 6-month seizure remission based on seizure report
	To determine if a PBS intervention is superior to standard care with respect to 3-month sleep problem frequency measured by CSHQ	Total sleep problem score as measured by the CSHQ
**Primary (economic)*:**	To estimate the cost-utility of carbamazepine, levetiracetam and PBS	Total costs based on resource use questionnaires, and routine hospital data; and quality-adjusted life years (QALYs) based on health utilities
**Secondary:**	To compare time to treatment failure due to inadequate seizure control or unacceptable adverse reactions	Time taken from randomisation to decision by child, parent or treating physician to be withdrawn from treatment due to inadequate seizure control or unacceptable adverse reactions
	To compare time to treatment failure due to inadequate seizure control	Time taken from randomisation to decision by child, parent or treating physician to be withdrawn from treatment due to inadequate seizure control
	To compare time to treatment failure due to unacceptable adverse reactions	Time taken from recruitment to decision by child, parent or treating physician to be withdrawn from trial due to unacceptable adverse reactions
	To compare time to first seizure	Time to first seizure based on seizure report
	To compare time to 12-month remission from seizures	Time to 12-month seizure remission based on seizure report

* This has no implications for sample size calculation

	To determine if a PBS intervention is superior to standard care	Total sleep problem score as measured by the CSHQ
	To compare measures of cognition across the different treatment groups	Total score in three chosen assessments delivered by CANTAB iPad Neuropsychological battery
	To compare Health Related Quality of Life across the different treatment groups	CHEQOL score change (1)
	To compare measures of children's behaviour across the different treatment groups	Total score on strengths and difficulties questionnaire (SDQ)
	To identify any adverse reactions and their rate	Records of adverse reactions
	To estimate child health utilities and QALYs across the different treatment groups	Score changes in:<br>1. CHU9D (2)<br>2. EQ-5D-Y
	To estimate health utilities and QALYs across parents in the different treatment groups	EQ-5D-5L score change
	To compare parenting self-efficacy across the different treatment groups	Score changes in:<br>1. PSAM (3)
	To compare sickness related school absences across the different treatment groups	Total sickness related school absences (days)
	To determine the costs to the NHS and personal social services (PSS)	1. Resource use questionnaire<br>2. Patient Level Information and Costing System (PLICS) data<br>3. Hospital Episode Statistics (HES)
**Other:**	To determine which sleep parameters change in primary carer and child dyads in different treatment groups	Summary of actigraphy variables (total sleep time/sleep latency/sleep efficiency) averaged over a 1 week period

2.1 Sub-study Summary:

**Title:**	Qualitative Interview Study	
**Sample size:**	Trial participants:<br>50 parent-child dyads or until saturation, whichever comes first<br>Trial decliners:<br>10 parent-child dyads	
**Sub-study Inclusion Criteria :**	Parent-child dyads eligible to take part in the main trial will also require:<br>a. Ability of parent and child to speak conversational English.<br>b. Additional consent to main trial: Consent of parent to participate and for their child to participate	
**Patient Sub-study Duration:**	12 months	
**Overall Sub-study Duration:**	60 months	
	**Objective**	**Outcome Measures**
**Primary:**	To explore and capture qualitative information relating to children's and their parents' experiences of the decisions and choices they make regarding the treatment and management of RE	Face-to-face/telephone or skype interviews (audio recorded and transcribed)

Figure 1: Schematic of Trial Design

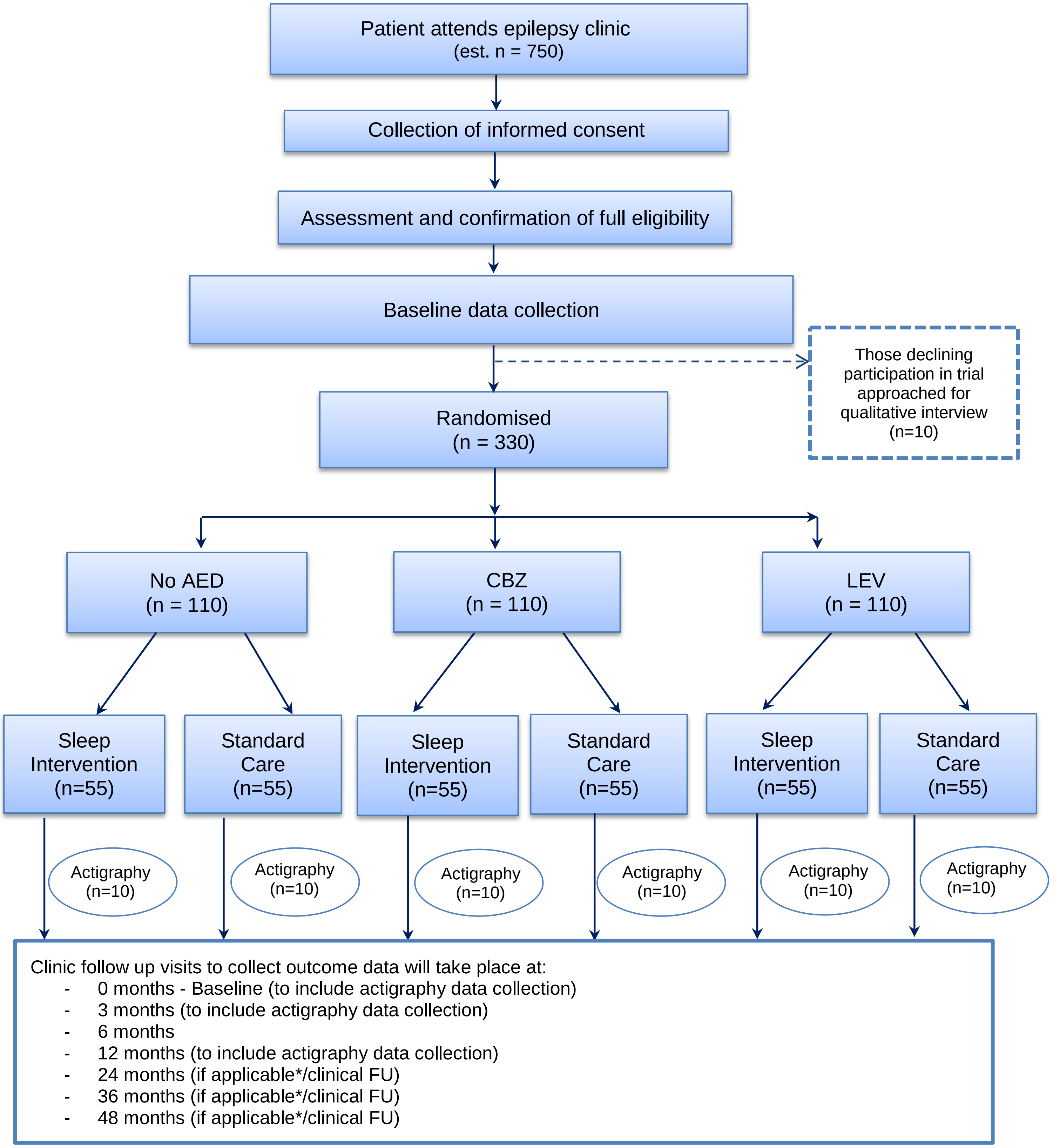

** The trial will be open to recruitment for 36 months. Follow up will be carried out on those recruited during the recruitment period and for 12 months after recruitment is closed. Therefore, if a patient joins the trial in the first month of opening to recruitment, the follow-up will be up to 48 months. In contrast, if the patient joins the trial just before closing recruitment, the follow-up will only last a maximum of 12 months.*

3 INTRODUCTION

3.1 Background

3.1.1 Rolandic Epilepsy

RE, also known as benign epilepsy of childhood with centro-temporal spikes (BECTS), is the most common epilepsy in childhood, with an incidence of up to 21 per 100.000 children aged 15 years and under. The onset of seizures is between 3 and 12 years, and remission almost always occurs by adolescence. Seizures typically occur during sleep or drowsiness, are brief and involve unilateral sensorimotor symptoms (e.g. numbness, tingling, drooling) of the pharynx, tongue, face, lips and sometimes hand. The affected side may alternate and seizures may infrequently become secondarily generalised. Neurodevelopmental disorders are very common (40%) and include speech sound disorder, language impairment and reading disability, all of which usually precede seizures and aggregate among relatives. Attention deficit hyperactivity disorder (ADHD) and migraine without aura are also strong but less frequent (10%) associations.

3.1.2 The Clinical Trial

This clinical trial is the major component of a programme of work on childhood epilepsy interventions and outcomes. Complementary work packages include the development of a core outcome measure set (Work Package 1); qualitative methods (Work Package 2); and the generation of an online parent-based sleep (PBS) intervention (also referred to as sleep behaviour intervention) for use in the trial (Work Package 3). We will conduct a pragmatic, unblinded, randomised 3x2 factorial trial of anti-epileptic drug (AED) (carbamazepine or levetiracetam or no AED) and sleep behaviour intervention or standard care, to evaluate their comparative effect on seizures, sleep problems, learning and patient-centred outcomes. In addition, engagement via patient and public involvement (PPI) advisory panel(s) will ensure that each stage of the programme is informed by the perspectives of children with rolandic epilepsy (RE) and their parents. An embedded economic evaluation will assess the cost-effectiveness of each intervention, from the perspective of the NHS and PSS.

3.2 Rationale

Choice of Anti-Epileptic Drug (AED)

The evidence base for treatment in childhood epilepsies in general, and for RE in particular, is scant (4, 5) and the quality of available evidence is judged in an Health Technology Assessment (HTA) monograph by the International League Against Epilepsy, and by National Institute for Health and Care Excellence (NICE) as “poor” (4, 6, 7). A Cochrane review in RE identified four randomised controlled trials (RCTs) of AED treatment in 262 children with RE. One good quality placebo-controlled RCT showed that sulthiame (not widely available in UK) is effective for seizure remission in the short-term (3-6 months), although the precision of the effect estimate was uncertain due to small sample size (5). Three low quality open-label AED comparative studies did not show differences in effectiveness or side-effects (5) amongst different AEDs (levetiracetam *vs* oxcarbazepine; clobazam *vs* carbamazepine; topiramate *vs* carbamazepine). The review concludes “there is insufficient evidence about the medium to longer effects on seizure control, the optimum AED and the effects of AED on cognition […] there is a need for good quality RCTs to address these questions [...]”.

Practice

Carbamazepine, a first-generation AED, is the current NICE standard treatment for RE (7). Our national survey confirmed that UK paediatricians prescribed carbamazepine as first-line choice (80%),

although there was great variability of choice amongst the remainder (8). Importantly, the survey revealed that in 40% of cases paediatricians treat RE patients conservatively, i.e. without drugs, as advocated in older textbooks (8). Carbamazepine has never been compared against no-treatment in RE, and in view of the AED's known cognitive adverse effects, it is important to know whether any benefit in terms of seizure control offsets its impact on children's learning. Second-generation AEDs (levetiracetam, lamotrigine, gabapentin, but not topiramate) have fewer cognitive side-effects in healthy adult volunteers than first-generation (carbamazepine, oxcarbazepine, valproate, phenobarbital, phenytoin) (9-15); and the International League Against Epilepsy judges only levetiracetam, of these second-generation AEDs, to have potential efficacy in RE (4). A generic version of levetiracetam was released in 2014 with comparable treatment cost to carbamazepine. Although not licensed for monotherapy in children, it is in widespread off-label use and is judged to be efficacious and well-tolerated (16). Thus levetiracetam would make an ideal and potentially superior comparator and has not been assessed head-to-head with carbamazepine in childhood epilepsy. NICE also made a specific research recommendation ([CG137:4.1]) for "levetiracetam to be evaluated as a first-line monotherapy in a prospective RCT [...] including *quality-of-life and cognitive outcomes*". The trial would complement the ongoing HTA-funded Standard and New Antiepileptic Drugs (SANAD-II) trial of levetiracetam, zonisamide and lamotrigine (Arm A: focal seizures), which has principally recruited adults (http://www.nets.nihr.ac.uk/projects/hta/0914409). The economic evaluation will address uncertainties and gaps in the evidence with respect to determining which interventions are cost-effective for use in practice and for broader adoption by the NHS.

Design considerations

We have assessed the feasibility of the proposed RCT among 132 UK paediatricians who are local leads for epilepsy management, and they expressed both equipoise over the benefits of treatment *vs* no-treatment in RE and acceptance to randomise to a no-treatment arm; they also indicated carbamazepine and levetiracetam as their preferred active comparators. A pragmatic design is chosen to provide guidance on decisions for clinical care contexts. Although we will capture seizure data, we will also give prominence to patient-relevant and learning outcomes as these are likely to relate to quality-of-life. In-depth interviews from the embedded qualitative study will explore and capture experiential data.

Sleep Intervention

Despite the frequency of sleep problems in children with epilepsy (and other neurodevelopmental disorders), clinicians in the UK receive little training on the subject (17, 18). This lack of training, and lack of epilepsy-specific evidence-based interventions, combined with a focus on seizure control means that sleep problems are rarely addressed in routine clinical practice. Even if sleep problems were a management target, the lack of any evidence-based resources for intervention is a barrier to such implementation. The PBS intervention offers parents education about normal sleep, advice about sleep-promoting practices and targeted strategies parents can employ to help their children to ''learn'' an appropriate set of sleep behaviours/habits and/or to unlearn inappropriate sleep behaviours.

The PBS intervention will include several evidence-based behaviour-change techniques including: extinction (unmodified or graduated with parental presence); scheduled wakings; positive routines with or without response cost; bedtime fading and positive reinforcement.

3.2.1 Risk and Benefits

The recruiting clinician will discuss the potential risks and benefits with patients and their parents/primary carers prior to trial entry and they will be outlined in the patient and parent/guardian information leaflets. Young women of child-bearing age will not be excluded from the trial because equipoise similarly exists about the merits of treatment and non-treatment of seizures in RE in this group. Monitoring of this group will be as in standard clinical practice with no mandatory pregnancy testing. Participants should receive individual counselling, guidance on contraception, interactions with contraceptive medications and the risks of teratogenicity as in routine medical practice.

The trial randomisation arms represent clinical pathways in current practice and as such the risks of participating in the trial are no greater than those encountered in standard care. The main risk of the CASTLE trial is that patients may be allocated to a treatment that on final analysis is found to be less effective than or have a higher adverse reaction (AR) rate than another. However, participants will be switched from their allocated treatment if a decision is made with the treating physician that seizure control is inadequate or adverse reactions (ARs) are unacceptable. For participants involved in the qualitative component, any concerns raised within the interviews will be reported as appropriate.

3.2.2 Known Potential Benefits

Patients recruited into the trial will receive standard NHS care during the conduct of the trial. The main potential benefit is that patients might receive treatment with a drug which is more effective than no AED. Patients might also receive benefit from the PBS intervention in terms of improved sleep compared to standard treatment. Patients may also feel benefit from a regular and rigorous follow-up schedule.

3.3 Objectives and Outcome Measures/Endpoints

Table 1: Summary of Objectives, Outcome Measures and Timepoints

**Primary Objectives**	**Outcome Measures**	**Timepoint(s) of evaluation of this outcome measure**
To determine if carbamazepine or levetiracetam are superior to no AED with respect to time to 6- month seizure remission.	Time to 6-month seizure remission based on seizure report	3, 6,12 months and annually thereafter - ‘when was last seizure’ question in clinic
To determine if a PBS intervention is superior to standard care with respect to 3-month sleep problem frequency measured by CSHQ	Total sleep problem score as measured by the CSHQ	At baseline and 3 months
**Primary Economic Objective†**	**Outcome Measures**	**Timepoint(s) of evaluation of this outcome measure**
To estimate the cost-utility of carbamazepine, levetiracetam and PBS	Total costs; and quality-adjusted life years (QALYs)	Resource use and utility questionnaires at baseline, 3, 12 months and annually

† This has no implications for sample size calculation

		thereafter; routine hospital data at the end of the trial
**Secondary Objectives**	**Outcome Measures**	**Timepoint(s) of evaluation of this outcome measure**
To compare time to treatment failure due to inadequate seizure control or unacceptable adverse reactions	Time taken from randomisation to decision by child, parent or treating physician to be withdrawn from treatment due to inadequate seizure control or unacceptable adverse reactions	3, 6,12 months and annually thereafter
To compare time to treatment failure due to inadequate seizure control	Time taken from randomisation to decision by child, parent or treating physician to be withdrawn from treatment due to inadequate seizure control	3,6,12 months and annually thereafter
To compare time to treatment failure due to unacceptable adverse reactions	Time taken from recruitment to decision by child, parent or treating physician to be withdrawn from trial due to unacceptable adverse reactions	3,6,12 months and annually thereafter
To compare time to first seizure	Time to first seizure based on seizure report	3,6,12 months and annually thereafter 'when was last seizure' question in clinic
To compare time to 12-month remission from seizures	Time to 12-month seizure remission based on seizure report	3,6,12 months and annually thereafter 'when was last seizure' question in clinic
To determine if a PBS intervention is superior to standard care	Total sleep problem score as measured by the CSHQ	At 12 months and annually thereafter
To compare measures of cognition across the different treatment groups	Total score in three chosen assessments delivered by CANTAB iPad Neuropsychological battery	At baseline, 3 and 12 months and annually thereafter
To compare Health Related Quality of Life across the different treatment groups	CHEQOL score change (1)	At baseline and 12 months and annually thereafter
To compare measures of children's behaviour across the different treatment groups	Total score on strengths and difficulties questionnaire (SDQ)	At baseline and 12 months and annually thereafter
To identify any adverse reactions and their rate	Records of adverse reactions	Adverse reactions at 3,6,12,24 months and annually thereafter in clinic

To estimate child health utilities and QALYs across the different treatment groups	Score changes in:<br>1. CHU9D (2)<br>2. EQ-5D-Y	Questionnaire at baseline, 3, 12 months and annually thereafter
To estimate health utilities and QALYs across parents in the different treatment groups	EQ-5D-5L score change	Questionnaire at baseline, 3, 12 months and annually thereafter
To compare parenting self-efficacy across the different treatment groups	Score changes in:<br>1. PSAM (3)	Questionnaire at baseline, 3, 12 months and annually thereafter
To compare sickness related school absences across the different treatment groups	Total sickness related school absences (days)	3,6,12 months and annually thereafter 'sickness related school absences' question in clinic
To determine the costs to the NHS	1. Resource use questionnaire<br>2. Patient Level Information and Costing System (PLICS) data<br>3. Hospital Episode Statistics (HES)	Resource use questionnaire at 3, 12 months and annually thereafter<br><br>PLICS and HES at end of trial
**Other Objectives**	**Outcome Measures**	**Timepoint(s) of evaluation of this outcome measure**
To determine which sleep parameters change in primary carer and child dyads in different treatment groups	Summary of actigraphy variables (total sleep time/sleep latency/sleep efficiency) averaged over a 1-week period	1 week actigraphy (arranged centrally via Oxford unit) at baseline, 3 and 12 months

**Sub-study Objective**	**Outcome Measures**	**Timepoint(s) of evaluation of this outcome measure**
To explore and capture qualitative information relating to children's and their parents' experiences of the decisions and choices they make regarding the treatment and management of RE	Face-to-face/telephone or skype interviews (audio recorded and transcribed)	2-4 weeks, 6 months and 12 months after randomisation

4 TRIAL DESIGN

The trial is a phase IV randomised factorial design controlled trial comparing carbamazepine, levetiracetam or active monitoring combined with or without sleep behaviour intervention. We have used a factorial trial design as this approach enables the efficient simultaneous investigation of AED (carbamazepine; levetiracetam; no AED) and sleep behaviour intervention (*vs* standard care) by including all participants in both analyses. In a factorial trial it is also possible to consider both the separate effects of each intervention and the benefits of receiving both interventions together (for example levetiracetam and sleep intervention).

5 TRIAL SETTING, SELECTION OF CENTRES/CLINICIANS

The CASTLE trial will take place in NHS out-patient paediatric epilepsy and general paediatric clinics in the UK.

5.1 Selection of Centres/Clinicians

Criteria for the selection of centres will be determined by the Trial Management Group (TMG) and will be described in the supplementary document 'Site Suitability Assessment'.

Initiation of centres will be undertaken in compliance with CTRC Standard Operating Procedures (SOPs); centres fulfilling the criteria will be selected to be recruitment centres for the CASTLE trial and will be opened to recruitment upon successful completion of all global (e.g. governance, ethical, and regulatory approvals) and study-specific conditions (e.g. site personnel training requirements) and once all necessary documents have been returned to CTRC as detailed in the trial 'greenlight' checklist.

Participant identification centres (PICs) might also be used to assist with patient recruitment. A centre will be defined as PIC when the hospital does not have enough resources to carry out the trial. PICs will refer patients to participating centres, where consent will be taken.

6 TRIAL POPULATION

6.1 Inclusion Criteria

Patients with the following characteristics will be eligible for inclusion in the trial:

a. Children diagnosed with RE (see International League Against Epilepsy Diagnostic Manual at https://www.epilepsydiagnosis.org/syndrome/ects-overview.html)
b. EEG showing focal sharp waves with normal background (see International League Against Epilepsy Diagnostic Manual at https://www.epilepsydiagnosis.org/syndrome/ects-eeg.html)
c. Aged ≥5 years and <13 years at the time of randomisation
d. Currently untreated with antiepileptic drugs
e. Written informed consent received from person with parental responsibility/legal representative.
f. Family have an email address and regular internet access (for online sleep intervention)
g. Parent and child are to have a good understanding of the English language

6.2 Exclusion Criteria

Patients with the following characteristics will be excluded from the trial:

a. Known contraindication to any of the trial drugs
b. Previously treated for epilepsy with antiepileptic drugs

6.3 Sub-study Inclusion Criteria

Parent-child dyads meeting the trial criteria and the following characteristics will be eligible for inclusion in the qualitative sub-study:

a. Ability of parent and child to speak conversational English.
b. Additional consent to main trial: Consent of parent to participate and for their child to participate.

6.4 Co-enrolment Guidelines

To avoid potentially confounding issues and to minimise the burden on participants, ideally participants should not be recruited into other clinical trials during their participation in CASTLE. Where recruitment into another trial is considered to be appropriate and without having any detrimental effect on the CASTLE trial this must first be discussed with the CTRC who will contact the Chief Investigator (Prof Deb Pal).

7 RECRUITMENT AND RANDOMISATION

7.1 Participant Identification and Screening

All patients aged ≥5 and <13 years with diagnosis of RE previously untreated with AED will be screened at the trial centres to identify potentially eligible participants for the trial. A 'Screening log' will be maintained of all the patients who undergo screening regardless of whether they are assessed as eligible or decide to participate in the trial as this will provide important information for monitoring purposes. Reasons for not being eligible will be recorded. Reasons for declining to participate will be asked routinely but it will be made clear that patients, parents or carers do not have to provide a reason unless they wish to do so.

Potentially eligible patients (i.e. those that meet the eligibility criteria) and those providing consent (person with parental responsibility/legal representative) will be invited to participate in the trial and provided with a patient information sheet and consent (PISC) form. The patient and the person providing consent will be allowed sufficient time to discuss the trial and decide whether to assent/consent to trial entry (see section 7.2 for assent and consent procedures).

7.2 Informed Consent

Informed consent is a process initiated prior to an individual agreeing to participate in a trial and continues throughout the individual's participation. Informed consent is required for all patients participating in CTRC coordinated trials. In obtaining and documenting informed consent, the investigator should comply with applicable regulatory requirements and should adhere to Good Clinical Practice (GCP) and to the ethical principles that have their origin in the Declaration of Helsinki.

7.2.1 Consent procedures

Proxy consent from an appropriate adult (a person with parental responsibility for the child or a person acting as the child's Legal Representative) is required to be obtained in CASTLE due to eligible patients being minors in accordance with the Medicines for Human Use (Clinical Trial) Regulations 2004 and amendments.

Proxy consent should be obtained prior to each patient participating in the trial, after a full explanation has been given of the treatment options, including the conventional and generally accepted methods of treatment.

Discussion of objectives, risks and inconveniences of the trial and the conditions under which it is to be conducted are to be provided to minors (to an extent appropriate for each minor's capacity of understanding) and the appropriate adult providing consent on their behalf by a medically qualified doctor with experience in obtaining informed consent in this population. Patient information and consent forms, describing in detail the trial interventions/products, trial procedures and risks will be approved by CASTLE's Research Ethic Committee (REC) and the appropriate adult will be asked to read and review the document.

Upon reviewing the document, a delegated member of the research team will explain the research trial to the minor (to an extent appropriate for each minor's capacity of understanding) and the appropriate adult. This information will emphasise that participation in the trial is voluntary and that the participant may withdraw from the trial at any time and for any reason. All minors and appropriate adults will be given opportunity to ask any questions that may arise and should have the opportunity

to discuss the trial and time to consider the information prior to agreeing to participate. A contact point where further information about the trial may be obtained will be provided.

Minors and the appropriate adults providing consent on their behalf will have as long as they require to consider their decision to join the trial or not and they may join the trial on the same day if that is their wish. The right of the appropriate adult approached for consent (person with parental responsibility / Legal Representative) to refuse consent for the minor to participate in the trial without giving reasons must be respected.

If willing to provide consent, the appropriate adult will then sign and date the informed consent document. Both the person taking consent and the appropriate adult must personally sign and date the form. A copy of the informed consent document will be given to the appropriate adult for their records. The original will be filed in the Investigator Site File (ISF) and a further copy will be retained in the participant's notes. One final copy of the consent form should be sent to the CTRC.

After the patient has entered the trial, the clinician will remain free to give alternative treatment to that specified in the protocol, at any stage, if s/he feels it to be in the best interest of the patient. However, the reason for doing so should be recorded and the patient will remain within the trial for the purpose of follow-up and data analysis. Similarly, the appropriate adult who has provided consent on behalf of the patient remains free to withdraw the patient at any time from the protocol treatment and trial follow-up without giving reasons and without prejudicing the further treatment of the minor.

7.2.2 Parental Responsibility; Who is eligible to provide proxy consent?

Parental consent may be obtained from those with parental responsibility for the child in accordance with the Clinical Trials Regulations (SI 2004/1031 as amended).

7.2.3 Assent of minors

If capable, and under appropriate circumstances, minors of ≥7 years should be approached to provide assent by a medically qualified doctor with experience with minors. Age-and-stage-of-development-appropriate REC-approved patient information sheet and assent forms, describing (in simplified terms) the details of the trial interventions, trial procedures and risks, are available. The minor should **personally** write their name and date the assent form (or otherwise personally mark the form, as they are able), which is then signed by the appropriate adult providing consent in the minor's behalf and the researcher taking assent.

Assent forms do not substitute the consent form signed by the appropriate adult providing consent on the minor's behalf (see section 7.2.1). Assent should be obtained where appropriate and documented in the patient notes. Minors who are approached for assent must provide this in order to be entered into the trial; the original completed assent form must be retained in the ISF, a copy given to the minor and another copy filed in the patient medical notes and a final copy sent to CTRC. However, the absence of assent does not exclude the patient, provided it has been deemed inappropriate to approach the minor for assent and consent has been obtained from the appropriate adult. Reasons for not approaching a minor for assent must be recorded in the patient medical notes and notified to CTRC.

7.2.4 Consent for the qualitative sub-study

As part of the CASTLE consent process, a delegated member of the centre research team will ask whether appropriate adults providing consent are happy for their contact details to be communicated

to the qualitative study team in order for them to be provided with further information so they may consider participating in the qualitative sub-study. The sub-study will require participation of both the child and the appropriate adult.

Additional consent and assent will be sought for the qualitative sub-study by a delegated member of the qualitative research team. Children with RE will only be invited to interview once a person with parental responsibility for the minor (see section 7.2.2) has consented to participation in the sub-study and has given permission for the minor to be approached for assent. Only minors ≥7 years that are capable will be asked to assent. Persons with parental responsibility for the minor will consent for theirs and the minor's participation. This component acknowledges that children and parents are experts of their own experience. Consequently, inherent power differences (research-participant, adult-child, parent-child) will be sensitively handled. Materials and documentation will be tailored according to specific developmental abilities and produced in collaboration with the PPI panel.

7.2.5 Re-consent of minors who reach 16 years during trial participation

For the purposes of the trial, a minor is defined as under 16 years. Should a trial participant reach age 16 years during the course of their trial participation, they will be re-consented as an adult.

7.3 Where consent to participate in CASTLE is declined

A delegated member of the centre research team may approach those who have declined to participate in CASTLE to request whether they are happy for their contact details to be communicated to the qualitative sub-study team in order for them to be provided with further information so they may consider participating in the qualitative sub-study.

7.4 Enrolment/Baseline

Once consent has been obtained from the appropriate adult, and assent from the child if appropriate, by the delegated member of the research team the eligibility assessments will be completed, full eligibility confirmed (confirmation must be by a medically qualified doctor) and baseline data will be collected prior to randomisation.

7.5 Randomisation Procedures

Patients **should not** be randomised until:

a) Written informed consent has been obtained from an appropriate adult and assent if appropriate obtained from the patient;
b) All eligibility criteria have been fulfilled and full eligibility has been confirmed by a medically qualified doctor and this has been recorded in the patient's medical notes and on the Eligibility case report form (CRF);
c) The Baseline CRF has been completed.

All participants will be randomised firstly between carbamazepine or levetiracetam or active monitoring in a 1:1:1 ratio, and secondly between PBS or standard care in a 1:1 ratio (see flow diagram, Figure 1). A subset of participants will be purposively selected to wear an actigraphy watch and participate in the actigraphy sleep monitoring component.

If allocated to carbamazepine or levetiracetam, the randomised treatment should ideally begin on the day of randomisation, or within 14 days of randomisation at the latest. If the participant has been selected to wear an actigraph, the treatment should not commence until the actigraph has been worn for the first week (however treatment should still commence within the 14 day window after randomisation). Carbamazepine or levetiracetam treatment should be sourced via usual NHS arrangements (see section 9).

Participants will be randomised using a secure (24-hour) web-based randomisation programme. Allocation sequences will be computer-generated, and concealed from staff recruiting participants to the trial using a secure internet-based system created and maintained by the CTRC. A personal login username and password, provided by the CTRC, will be required to access the randomisation system; designated research staff will be issued with their personal login and password upon completion of training in the use of the system.

When the system requirements (consent and eligibility) are confirmed, the participant intervention allocations and a unique trial number (randomisation number) will be displayed on a secure webpage and an automated email confirmation will be sent to the designated centre personnel and to the trial coordinator (only those recipients who are required to do so will receive information of intervention allocations in the notification email). It is the responsibility of the Principal Investigator (PI) or delegated research staff to inform the pharmacy department at their centre prior to randomisation to ensure there is sufficient supply of the trial treatments.

Randomisation: web access https://ctrc.liv.ac.uk/Randomisation/CASTLE/
If there are any problems with the randomisation systems contact the CTRC on 0151 795 8774 or via email on castle.rct@liv.ac.uk

(Note that the coordinating CTRC is open from 09:00 – 17:00, Monday – Friday, excluding public holidays)

7.5.1 Back up Randomisation Process

In the event of a randomisation system failure, the centre should contact the coordinating team in CTRC (Monday to Friday between 9:00 to 17:00 excluding bank holidays) to try to resolve the problem. If the problem cannot be resolved, the CTRC will perform central randomisation and randomise the participant using the back-up randomisation system. The back-up randomisation system is an exact replica of the live system but is based on a standalone PC at CTRC.

7.5.2 Contacts Database

The ‘contacts database’, also called CASTLE Contact Management System, is a secure system hosted by the University of Liverpool designed to be used immediately after randomisation. The system sits between the randomisation system and the online PBS intervention and will collect the required personal and contact details from participants if they have:

- Been allocated to receive the PBS intervention

and/or

- Been selected for actigraphy (and consented to actigraphy)

The required details will be entered in the contacts database by the research team at site. A personal login username and password, provided by the CTRC, will be required to access the contacts database; designated research staff will be issued with their personal login and password upon completion of training in the use of the system. When all fields in the contacts database have been completed and submitted by the research staff at site, an email will be automatically generated and securely sent to:

- the primary carer of the participant, which will include a link to the PBS intervention online and log in details (if the participant is randomised to the PBS arm).

and/or

- the sleep research team at Oxford Brookes University to inform them that a participant has been added so they can view the address and contact the parent/primary carer to arrange the shipment of the actigraphs (if the participant is selected for actigraphy and has consented to this).

The system is password protected and accounts will only be generated for specific members of the sleep research team at Oxford Brookes University and CTRC members that need access to participant's details.

7.5.3 Qualitative Sub-Study Identifiers

As an exception, the screening number will be used to identify qualitative sub-study participants (instead of their randomisation number) by the qualitative team. This will allow the sub-study to have an identifier for both main trial accepters and decliners that is unique.

7.6 Who is Blinded to Allocations

Data analysts will be blinded after assignment to interventions by assignment of randomisation numbers to participants.

8 PARTICIPANT TIME LINE, ASSESSMENTS AND PROCEDURES

8.1 Participant timeline

8.1.1 Schedule of Trial Procedures by Visit

Trial procedures are listed below organised per visit and order of priority.

0 Months (Baseline) – visit 1:

- Written Informed Consent (and assent where appropriate)
- Review of Medical History and EEG results
- Assessment of Eligibility Criteria and confirmation of full eligibility

- Measurement of weight and height
- Review of seizure occurrence & hospital admissions
- Children's Sleep Habits Questionnaire (CSHQ) – *primary carer*
- CANTAB test (iPad) – *child*
- Health Related Quality of Life in Children with Epilepsy (CHEQOL) – *primary carer & child (≥8 years)*
- Strengths and Difficulties Questionnaire (SDQ) – primary carer
- CHU9D Questionnaire – *child*
- EQ-5D-Y Questionnaire – *child ≥ 8 years*
- EQ-5D-5L Questionnaire – *primary carer*
- Parenting Self Agency Measure (PSAM) 5 item version Questionnaire – *primary carer*

0 Months (Randomisation) – visit 1:

- Randomisation to Trial Interventions
- Completion of Contact Details Database
- Confirmation of selection for the actigraphy subset

(At home: actigraphs are sent to the participant's address for those in the subset. Dyads will wear the actigraphs for 1 week)

3 Months – visit 2:

- Confirm continuing trial intervention

- CSHQ Questionnaire – *primary carer*
- CANTAB test (iPad) – *child*
- CHU9D Questionnaire – *child*
- EQ-5D-Y Questionnaire – *child ≥ 8 years*
- Resource Use Questionnaire – *primary carer*
- EQ-5D-5L Questionnaire – *primary carer*
- PSAM Questionnaire – *primary carer*

- Review of seizure occurrence & hospital admissions
- Assessment of Adverse Events
- Completion of Follow-up CRF
- Completion of AED treatment CRF

- Completion of concomitant medications related to an adverse reaction CRF

(At home: actigraphs are sent to the participant's address for those in the subset. Dyads will wear the actigraphs for 1 week)

6 Months – visit 3:

- Confirm continuing trial intervention
- Review of seizure occurrence & hospital admissions
- Assessment of Adverse Events
- Completion of Follow-up CRF
- Completion of AED treatment CRF
- Completion of concomitant medications related to an adverse reaction CRF

12 Months – visit 4:

- Confirm continuing trial intervention
- CSHQ Questionnaire – *primary carer*
- CANTAB test (iPad) – *child*
- CHEQOL Questionnaire - *primary carer & child (≥8 years)*
- SDQ Questionnaire – *primary carer*
- CHU9D Questionnaire – *child*
- EQ-5D-Y Questionnaire – *child ≥ 8 years*
- Resource Use Questionnaire – *primary carer*
- EQ-5D-5L Questionnaire – *primary carer*
- PSAM Questionnaire – *primary carer*
- Review of seizure occurrence & hospital admissions
- Assessment of Adverse Events
- Completion of Follow-up CRF
- Completion of AED treatment CRF
- Completion of concomitant medications related to an adverse reaction CRF

(At home: actigraphs are sent to the participant's address for those in the subset. Dyads will wear the actigraphs for 1 week)

24, 36, 48 Months – visits 5, 6, 7:

- Confirm continuing trial intervention
- CSHQ Questionnaire – *primary carer*
- CANTAB test (iPad) – *child*
-
- CHEQOL Questionnaire - *primary carer & child (≥8 years)*
- SDQ Questionnaire – *primary carer*
- CHU9D Questionnaire – *child*
- EQ-5D-Y Questionnaire – *child ≥ 8 years*
- Resource Use Questionnaire – *primary carer*
- EQ-5D-5L Questionnaire – *primary carer*
- PSAM Questionnaire – *primary carer*

- Review of seizure occurrence & hospital admissions
- Assessment of Adverse Events
- Completion of Follow-up CRF
- Completion of AED treatment CRF
- Completion of concomitant medications related to an adverse reaction CRF

Table 2: Schedule of Trial Procedures

Procedures	T0 Baseline[1]	T0 Randomi-sation	T0 +3 months (+/-2 weeks)	T0 +6 months	T0 +12 months	T0 +24 months[2]	T0 +36 months[2]	T0 +48 months[2]	T0 +X months Unscheduled visits
Written Informed Consent (and assent where appropriate)	X								
Review of Medical History and EEG results	X								
Weight and height	X								
Assessment of Eligibility Criteria and confirmation of full eligibility	X								
CSHQ Questionnaire – primary carer	X		X		X	X	X	X	
CANTAB test (iPad)- child	X		X		X	X	X	X	
CHEQOL Questionnaire–primary carer & child (≥8 years)	X				X	X	X	X	
SDQ Questionnaire – primary carer	X				X	X	X	X	
CHU9D Questionnaire – child	X		X		X	X	X	X	
EQ-5D-Y Questionnaire – child ≥8 years	X		X		X	X	X	X	
EQ-5D-5L Questionnaire – primary carer	X		X		X	X	X	X	
PSAM Questionnaire – primary carer	X		X		X	X	X	X	
Resource use Questionnaire – primary carer			X		X	X	X	X	
Randomisation to Trial Interventions		X							
Completion of Contact Details Database		X							
Actigraphy		(X)	(X)		(X)				
Confirm continuing trial intervention (as per randomisation or alternative anti-epileptic drug)			X	X	X	X	X	X	X
Review of seizure occurrence & hospital admissions	X		X	X	X	X	X	X	X
Assessment of Adverse Events			X	X	X	X	X	X	X
Completion of Follow-up CRF			X	X	X	X	X	X	X
Review of AED treatments and concomitant medications related to adverse reactions			X	X	X	X	X	X	X

[1] At baseline, written informed consent must be taken before performing any study procedures.
[2] Follow up will be a minimum of 12 months but can go beyond this, up to a maximum of 48 months, as trial timelines allow
(X) Measures only taken from the selected subset of patients that have consented to this activity

Table 3: Schedule of Sub-Study Interviews

Trial accepters:

Procedures	Trial Randomisation (T0)	Interview 1 (T 2 - 4 weeks)	Interview 2 (T6.5-7 months)	Interview 3 (T12.5-13 months)
Participants' contact details are sent to Edge Hill University (via CTRC)	X			
Sub-study written informed consent (and assent where appropriate)		X		
Qualitative interviews (for those who consent)		X	X	X

Trial decliners:

Procedures	Decision to not participate in the trial (T0)	Interview 1 (T 2 - 4 weeks)	Interview 2 (T6.5-7 months)	Interview 3 (T12.5-13 months)
Participants' contact details are sent to Edge Hill University (via CTRC)	X			
Sub-study written informed consent (and assent where appropriate)		X		
Qualitative interview (for those who consent)		X	X	X

8.1.2 Schedule for Follow-up

The expected duration of follow-up for each participant is between a minimum of 12 months and a maximum of 48 months, depending on the time of recruitment. All participants will be followed up whether they are still taking their allocated treatment or not. Where participants default from hospital follow-up, additional information will be sought at the end of the trial from GPs who will be the main prescribers of AEDs in this trial, and where necessary participants will be contacted directly for follow up information.

Participants will be followed up as per routine clinical practice and typically at 3, 6, and 12 months and annually thereafter, where applicable. Participants will be seen at other times as clinically indicated.

A visit window for the 3 month visit of +/- 2 weeks is allowed. Visit windows are not prescribed for the 6, 12 months or annual visits after that. A delegated member of the research team should complete the 'follow-up CRF' for the 3, 6, 12 month and annual visits. The visit which occurs at the nearest timepoint will be taken to be the record of the 3, 6, 12 months or other annual visits from randomisation.

In the event of an early trial termination, the schedule for follow up activities (including actigraphy and qualitative interviews) will be shortened accordingly to 6 months post-randomisation This will occur following agreement by the TMG, IDSMC and Sponsor.

8.2 Procedures for assessing Efficacy

8.2.1 Seizure outcomes

At each clinic visit scheduled per routine practice, and any unscheduled clinic visit, the number of seizures, date of first seizure, and date of most recent seizure experienced since previous follow-up visit (or baseline visit if attending the first clinic visit) will be recorded in the CRF. These data will be

used to calculate the primary outcome measure (time to 6-month remission from seizures) and seizure related secondary outcome measures (time to 12-month remission from seizures, time to first seizure).

At each clinic visit scheduled per routine practice and at any unscheduled clinic visit, the date of failure of randomised AED treatment and reason for failure will be recorded in the CRF if relevant. This data will be used to calculate the secondary outcome measure (time to treatment failure).

8.2.2 Health Related Quality of Life

The scores obtained for the CHEQOL questionnaire (1) throughout the trial can be used as a subjective measure of efficacy. The parent/primary carer will complete a proxy version of the questionnaire while the child (if ≥ 8 years) completes the CHEQOL child version. Both questionnaires will be delivered on paper at the specified timepoints (see Table 2: schedule of trial procedures).

8.2.3 Strengths and Difficulties Questionnaire

SDQ is a brief emotional and behavioural screening questionnaire that will be used to compare children across the different treatment groups. The scores obtained from this questionnaire throughout the trial can be used as a subjective measure of efficacy. SDQ will be completed by the parent/primary carer at the specified timepoints (see Table 2: Schedule of trial procedures).

8.2.4 Parent self-efficacy measure

The scores obtained for the Parenting Self-Agency Measure (PSAM) questionnaire (3) throughout the trial will be used as a subjective measure of parental efficacy. The parent/primary carer will complete the questionnaire at the specified timepoints (see Table 2: schedule of trial procedures).

A recent review showed that PSAM was a reliable domain for specific measures of parental efficacy. Although it came second to another measure called CAPES-SE in overall ‘quality’, the CAPES-ES was 20 items long (compared to PSAM which is 5) and also covered things that are already being tapped by other measures used in this trial (e.g. child behaviour challenges). Additionally, CAPES-SE focuses on how confident parents feel in managing these 'problems' rather than about parenting more broadly, which is what PSAM will be delivering and what we aim to capture (19, 20).

8.2.5 Sleep

8.2.5.1 Subjective Sleep

The primary sleep outcome uses the CSHQ (21). This is a standardised, widely used questionnaire to assess sleep problems in school-age children referenced in over 600 publications. Parents/primary carers will complete a paper-based questionnaire when they attend clinic. Parents/primary carers will be asked to recall events over the past typical week indicating the frequency of occurrence of a number of sleep related behaviours. In addition to information about sleep timing, the questionnaire gives both an overall sleep disturbance score and eight subscale scores: bedtime resistance, sleep onset delay, sleep duration, sleep anxiety, night-wakings, parasomnias, sleep-disordered breathing and daytime sleepiness, reflecting key areas of clinical concern.

8.2.5.2 Actigraphy

Although previous studies have demonstrated impaired sleep quantity and quality of parents of children with epilepsy (22), none have employed objective measures to demonstrate whether the impairment is related to a perceived or a measurable deficit in sleep parameters. Actigraphy is a wrist-worn, accelerometer-based method of objective sleep assessment widely used in research and clinical practice and has been used successfully with children, including those with a range of developmental disorders (23, 24).

Up to 15 dyads (child and primary carer) from each of the 6 final trial subgroups will both wear the watches (actigraphs) for 1 week at baseline, 3 and 12 months during the trial. However, only the data from a purposeful sample of 10 dyads from each group will be analysed; this is to account for any drop outs or incomplete data. Actigraphy data will let us look at baseline sleep patterns, the relationship between the primary carer and the child's sleep, and the impact of different treatments during the trial. Summary variables of sleep latency, total sleep time and sleep efficiency will be used for analysis. Along with the actigraphs, parents/primary carers will be asked to complete a sleep log that will capture 'lights out', subjective sleep times, when the watch was put on/off, exceptions circumstances, etc. Participants might also contribute to log completion if they have capacity to do so but parents are intended to be the key recorder. The information recorded in the sleep logs will be used to facilitate subsequent analysis of actigraphy data.

When a new participant is selected for actigraphy, the sleep research team at Oxford Brookes University will be notified via email so they can proceed to:

1. Log in to the contacts database and view the participant's name, address, phone number and email
2. Contact the parent/primary carer to arrange a convenient time for the shipping of the actigraph and answer any questions they might have
3. Ship the actigraphy equipment together with instructions in the use of the watches and its return, as well as the sleep log (paper document) and contact details for troubleshooting any problems. The shipment will ideally occur within 1-2 days after randomisation but it is acknowledged that it might not always be the case and it will depend on the timing of randomisation and the availability of the parent/primary carer to receive the package.

8.2.6 Learning

We will use the industry standard, tablet-based CANTAB (www.cantab.com), a set of cognitive tasks referenced in over 1,600 peer-reviewed papers. It has a number of advantages compared to other measures, including: extensive validation of test properties; normative values for children; easy administration that does not require trained psychologist; proven utility in adults and children with epilepsy including RE (25); responsive to interventions in childhood neurodevelopmental disorders (26) and sleep (27); and correlation with subjective 'paper-based measures' and childhood brain imaging datasets (28). In an RE trial, patients were compared pre- and post- AED treatment to controls using several CANTAB tasks; CANTAB revealed deficits in visual memory tests despite global cognitive development scores in the normal range (25). Participants will complete the chosen assessments delivered by CANTAB iPad Neuropsychological battery at baseline, 3, 12 months and annually thereafter (see Table 2: Schedule of trial procedures). CANTAB will be delivered on an iPad.

8.3 Procedures for Assessing Safety

An assessment of safety events will be undertaken at each trial visit. These reviews should be carried out by the PI or delegated research staff. Safety reporting is detailed fully in Section 10.

8.4 Other Assessments

8.4.1 Qualitative interviews

As part of the qualitative sub-study, additional consent will be gained from appropriate adults and assent from children (aged 7 or older) with RE to participate in interviews. Interviews will be conducted by the research team at Edge Hill University and they will either be face-to-face or remotely (via

telephone or Skype). The interviews will take place at three timepoints: the first interview will be scheduled at 2-4 weeks post randomisation and the other two at 6 months and 12 months respectively after interview 1). Interviews will be audio-recorded and transcribed. Appropriate adults and children with RE who have declined to participate in the trial, will additionally be approached and asked for consent to be interviewed 2-4 weeks after being approached to participate in the trial. Three interviews will also be scheduled for trial decliners: interview 1 will take place at 2-4 weeks after declining trial participation, and the other two interviews will take place at 6 months and 12 months respectively after interview 1.

8.4.2 Health Economics

The factorial trial design will allow for a comprehensive comparison for the determination of the cost-effectiveness of: levetiracetam, carbamazepine or active monitoring, separately and in combination with/without sleep intervention. We will adopt the perspective of the NHS and personal social services (PSS) in the primary analysis, and consider a broader perspective, approximating to a societal perspective in a secondary analysis. This latter analysis will include impacts on indirect costs such as educational attainment and loss of productivity.

Major direct costs of health care resources used by participants in the trial will be collected using a resource use questionnaire and administered during follow-up visits. The questionnaire will include items related to use of primary care services (including out-of-hours services), Accident & Emergency (A&E), healthcare advice lines such as NHS Direct or NHS 24 in Scotland, walk-in treatment centres, and home visits, additional tests/investigations performed, parental time off work etc. We will directly record participants' use of secondary care services directly using the trial CRFs, by assessing patient-level information and costing systems (PLICS) data from the finance departments of each recruiting hospital, and submitting requests for bespoke hospital episode statistics (HES) to national information, data and IT system providers (such as NHS Digital in England) at the end of the trial. This will ensure missing data is reduced to a minimum. The cost of hospital stays will be taken from the NHS reference costs database; other unit costs will be sought from appropriate sources.

PLICS data include Health Resource Groups which detail costs for patient stays and treatments. Responsibility for the data collection and anonymization will rest with the site research nurse who will supply their site Finance Departments with the necessary details to ensure only information on consented participating patients are provided. It is the responsibility of the site Finance Departments to provide the site research nurses with the data in a timely fashion and should the site research nurse so request, to ensure all patient identifying data have been replaced with the patient trial number. Anonymised PLICS data will be transferred securely from each site to the CTRC, and then on to the Centre for Health Economics and Medicines Evaluation (CHEME), Bangor University for analysis.

HES data will include information on outpatient, inpatient (including critical care) and A&E attendances by each participant from the beginning of the financial year immediately prior to the date of the first participant being randomised, to the date of last participant completing follow-up. The data will be collected centrally from NHS Digital (or national appropriate) who operate under a General Data Protection Regulation (GDPR) framework. Participants will be fully and unambiguously informed as to the transfer of any personal data associated with obtaining and processing their HES data and will agree to these processes via an opt-in method. Participants will explicitly be made aware that they have the right to withdraw consent, and how to go about withdrawing their consent, for use and disclosure of HES data.

Participant information (postcode, date of birth, NHS number (or equivalent) and randomisation number) will be collected by the CTRC to generate a secure database which will enable CTRC to request the HES data from NHS Digital (or national appropriate). The database will only be accessible by authorised personnel working on the trial. At the time of the data request, the database will be sent to NHS Digital (or national appropriate) via a secure link and the HES data with the trial number (and no other identifier) will be securely transferred to Bangor University CHEME. NHS Digital (or national appropriate) will be asked to remove participant personal identifiers such as NHS number, date of birth and pseudoHESID at source. Access to data will only be possible by username and password and restricted to health economists working on the trial. The HES data will be securely disposed in accordance with the CHEME and NHS Digital (or national appropriate) DSFCs.

Children will be asked to complete the CHU9D (2) and/or EQ-5D-Y questionnaires (29), where appropriate for age (see Table 2: Schedule of Trial Procedures), at intervals coinciding with other clinical and outcome measurements. In secondary analysis, parents/primary carers will complete the EQ-5D-5L questionnaire to estimate spill-over (dis)utilities in parents/primary carers. Quality-adjusted life-years (QALYs) will be estimated using UK tariff scores, where available, and calculated as the area under the utility curve for the duration of follow-up.

8.4.3 End of trial procedures

A panel will review all the clinical summaries and electroencephalography (EEG) reports of enrolled participants throughout the trial to assess diagnostic validity (centres will be asked to send the reports with personal identifiers redacted).

In the event of an early trial termination, a review of EEG reports will only commence following explicit agreement from the TMG and IDSMC.

8.5 Participant Transfer and Withdrawal

After consent to the trial is provided by the appropriate adult, trial treatment will be administered to the participants, along with follow-up processes and data collection.

8.5.1 Participant Transfers

For participants moving from the area, every effort should be made for the participant to be followed-up at another participating trial centre and for this trial centre to take over responsibility for the participant or for follow-up via GP. A transfer CRF will be completed by the old centre and returned to CTRC. CTRC will forward on to the new centre and the new PI will be asked to confirm that they have taken on responsibility for the patient in the trial. The old centre should provide a copy of the participant's CRFs to the new centre.

8.5.2 Stopping the Allocated Trial Intervention

Participants may be withdrawn from treatment for any of the following reasons:

a. Appropriate adult who provided consent withdraws consent (or, where applicable, the minor participant withdraws assent).
b. Unacceptable toxicity.
c. Intercurrent illness preventing further treatment.
d. Any change in the participant's condition that justifies the discontinuation of treatment in the clinician's opinion.

If a participant or the adult who provided consent on their behalf wishes to stop trial treatment, follow-up will continue unless the participant or appropriate adult explicitly also wishes to stop this. Centres should explain the importance of remaining on trial follow-up, or failing this, of allowing routine follow-up data to be used for trial purposes.

Note that if the allocated treatment is stopped due to a safety event, the participant will be given appropriate care under medical supervision until the symptoms of any safety event resolves or the participant's condition becomes stable.

8.5.3 Withdrawal from Trial Completely

Participants or their appropriate adults are free to withdraw assent/consent to continue in the trial at any time without providing a reason (but wherever possible information on the reason for withdrawal will be recorded). Those who wish to withdraw assent/consent to continue in the trial will have anonymised data collected up to the point that assent/consent is noted to be withdrawn included in the analyses. The participant will not contribute further data to the trial and the CTRC should be informed by completion of a Withdrawal CRF. Data up to the time of consent withdrawal will be included in the analyses in accordance with GDPR and DPA 2018.

Participants withdrawing from the qualitative sub-study will have their withdrawal documented by the qualitative study team.

8.6 Loss to Follow-up

If any of the trial participants are lost to follow-up, contact will initially be attempted through the research team at each centre. If the lead investigator at the trial centre is not the participant's usual clinician responsible for their speciality care, then follow-up will also be attempted through this latter clinician. Where these attempts are unsuccessful, the child's GP will be asked to provide follow-up information to the recruiting centre. Wherever possible, information on the reason for loss to follow-up will be recorded.

8.7 Trial Closure

The end of the trial is defined to be the date on which data for all participants is locked and data entry privileges are withdrawn from the trial database. However, the trial may be closed prematurely by the Trial Steering Committee (TSC), on the recommendation of the Independent Data and Safety Monitoring Committee (IDSMC).

9 TRIAL TREATMENT/INTERVENTIONS

9.1 Interventions

9.1.1 Randomised Drug Interventions or No Drug

Participants will be randomised to treatment with carbamazepine, levetiracetam or active monitoring. Where randomised to drug treatment, the randomised treatment should ideally begin on the day of randomisation or within 14 days of randomisation at the latest. The research team should ensure that the duration between obtaining consent, performing baseline assessments, randomisation and the start of trial treatment does not negatively impact on the well-being of the participant.

All treatments will be procured, prescribed and issued as per routine NHS practice. Following randomisation, the initial IMP dosing (for carbamazepine and levetiracetam arms) will be determined by an authorised medically qualified member of the trial site's research team, who will ideally issue the first prescription to allow trial treatment to commence within 14 days of randomisation. First prescription can also be issued by the GP if this follows routine practice for the site. Subsequently, repeat prescriptions will be issued by an authorised medical member of the trial site's research team or by the participant's local GP in accordance with the standard care pathway for this population. GPs will be informed of the participant's inclusion in the CASTLE trial, the trial treatment they have been allocated to, the dose to be prescribed and any modifications (where relevant). They will be instructed to issue repeat IMP prescriptions in the CASTLE GP Letter (where designated to do so by the PI), which will be submitted for ethical approval.

Randomised treatment will continue for a minimum of 12 months and a maximum of 48 months.

9.1.1.1 Actigraphy cohort

Baseline actigraphy is defined as the data collected from sleep patterns before the participant starts trial medication. The participants selected to wear an actigraph watch will be asked to wear the watch for one week and start medication and/or sleep intervention (if relevant) immediately after the actigraph has been removed. Actigraphs will be sent directly to the participants' address as soon as possible after randomisation (should be sent ideally within 1-2 days following randomisation). If actigraphs are not received before night 7 post-randomisation then the participants will not be able to wear the actigraphs as it will not allow treatment to commence within the 14 day window.

9.1.2 Randomised Sleep Intervention

At the same time as randomisation to carbamazepine, levetiracetam or active monitoring, participants will be randomised to a PBS intervention or no PBS intervention (standard care).

The PBS intervention is an e-learning package for parents/primary carers and children with epilepsy. The e-learning package will be referred to as COSI (CASTLE Online Sleep Intervention) in patient facing documents. Those randomised to receive the PBS intervention will receive an email directly from the contacts database that will contain a website link, access instructions and unique login details to access the sleep intervention program online (COSI). An email reminder will be sent to the parent/primary carer 3 days after randomisation if they have not accessed COSI.

The contacts database will also email the sleep research team at Oxford Brookes University to let them know that a new participant has been allocated to receive the PBS intervention.. If a parent/primary carer has not accessed COSI and therefore is not using the PBS intervention within 6

days of randomisation, the sleep team will get notified via email and will then give the parent/primary carer a reminder call.

When participants have been selected for actigraphy, the initial email with the COSI account details (username, password and link) will not be sent until day 12 after randomisation to allow enough time for parent/primary carer and participant to wear the actigraph before using the online sleep intervention. Due to this delayed start, the email reminder will be sent to the parent/primary carer 15 days after randomisation instead if they have not accessed COSI. Again, if the PBS intervention has not been used by day 18 after randomisation, the sleep team will get notified via email and will then give a reminder call to parents/primary carers.

After 3 months of account creation, parents/primary carers will receive an email informing them that an evaluation module has become available. If they do not complete the evaluation module within 6 days, the sleep team at Oxford Brookes will be notified and will then proceed to give parents/primary carers a reminder call.

Telephone numbers and email addresses are collected for all participants in the consent form and will be saved in the contacts database if they were allocated to the PBS arm or selected for actigraphy.

9.2 Additional Considerations for Drug Interventions

9.2.1 Formulation, Packaging, Labelling, Storage and Stability

CASTLE is a pragmatic trial using Investigational Medicinal Products (IMPs) which are market authorised products. These will be dispensed in accordance with a prescription given by a healthcare professional and a NHS dispensing label will be applied in accordance with standard practice (complying with Schedule 5 of the Medicines for Human Use (Marketing Authorisations Etc) Regulations 1994 (SI 1994/3144)). IMPs will be sourced using routine NHS stock and no modifications will be made to the products or their packaging. As such, a trial-specific label is not required for CASTLE IMPs in accordance with the Medicines for Human Use (Clinical Trials) Regulations (SI 2004/1031 as amended).

The IMPs will be dispensed by hospital and community pharmacies as they would be normally in clinical practice. It is the responsibility of the PI to ensure that the GP is prepared to prescribe the remainder of any treatment according to the randomised allocation that is not dispensed by the hospital pharmacy.

Although carbamazepine and levetiracetam generics can be prescribed, the reference safety information (RSI) to be used for the CASTLE IMPs is described in the following SmPCs:

Tegretol 200mg Prolonged Release Tablets – (Carbamazepine)

Marketing authorisation holder: Novartis Pharmaceuticals UK Ltd.
Carbamazepine Pharmacotherapeutic group: nervous system antiepileptics, antiepileptics, ATC code: N03AF01

**Active ingredient**	Carbamazepine
**Excipients**	Refer to current SmPC
**Pack Size(s)**	Refer to local supply chain (local pharmacy)

**Route of Administration**	Oral
**Storage temperature / time**	Refer to current SmPC
**Supplier's name**	Local supply chain (local pharmacy)

Each participating centre is advised to use the current version of the SmPC available from the eMC website (or equivalent) for treatment purposes.

Carbamazepine RSI: 4.8 section of the above SmPC will be used.

Keppra 250 mg Film-Coated Tablets – (Levetiracetam)

Marketing authorisation holder: UCB Pharma Limited
Levetiracetam Pharmacotherapeutic group: nervous system antiepileptics, antiepileptics, other antiepileptics, ATC code: N03AX14

**Active ingredient**	Levetiracetam
**Excipients**	Refer to current SmPC
**Pack Size(s)**	Refer to local supply chain (local pharmacy)
**Route of Administration**	Oral
**Storage temperature / time**	Refer to current SmPC
**Supplier's name**	Local supply chain (local pharmacy)

Each participating centre is advised to use the current version of the SmPC available from the eMC website (or equivalent) for treatment purposes.

Levetiracetam RSI: 4.8 section of the above SmPC will be used.

In addition to tablets, there are also liquid/syrup formulations of each IMP which can be used if appropriate. The below SmPCs will be considered RSIs for these liquid/syrup formulations:
- Tegretol (Carbamazepine) 100mg/5ml liquid
- Keppra (Levetiracetam) 100mg/ml oral solution

9.2.2 Preparation, Dosage and Administration of Trial Treatment/s

All participants should be titrated to an initial maintenance dose, with dose adjustments made at subsequent appointments according to clinical response and adverse effects. Only clinicians will choose the titration rate and initial maintenance they think is most appropriate for individual participants according to their usual practice.

9.2.3 Dose Modifications and Management of Toxicity

Dose modifications and management of toxicity will be undertaken by the PI or delegated medically qualified physician according to usual practice. The aim of treatment will be to control seizures with a minimum effective dose of drug. This will necessitate dosage modification if further seizures or ARs occur as is usual clinical practice. The decision to discontinue allocated trial treatment is at the discretion of the PI or delegated medically qualified physician and participant/their parent/primary carer. Treatment may be discontinued at any point during the trial period for reasons such as

inadequate seizure control, unacceptable ARs, or any change in the participant's condition that the physician believes warrants a change in medication. Any changes in medication must be documented on the appropriate follow up CRF along with the justification for those changes. If a participant's treatment stops prematurely, the reason for discontinuation should be recorded on the premature discontinuation on the trial treatment CRF and the participant should still be encouraged to attend follow up visits for the remainder of the trial. Any changes in trial treatment dosage must be documented on the trial treatment CRF along with the justification for those changes.

At the end of trial participation the participants may continue their treatment as per local policy.

9.2.4 Drug Accountability and Assessment of Compliance

There are no formal accountability measures required for the CASTLE trial, as treatments will be prescribed according to the local medical practices and dispensed by hospital and community pharmacies as they would be normally in clinical practice.

It is accepted that, for a variety of reasons including perceived or actual efficacy and tolerability, not all participants will take their medicines as prescribed. Participants will not be asked about adherence and no formal measurements of plasma drug levels are planned.

9.2.5 Precautions Required for Trial Treatments

Participants recruited into CASTLE will receive standard NHS care during the conduct of the trial and usual precautions will be applied when considering randomisation and making treatment decisions. Refer to the relevant SmPC for detailed guidance.

9.2.6 Concomitant Medications

Treating clinicians should monitor potential drug interactions from concomitant medications as detailed in the SmPC.

9.2.6.1 Data on Concomitant Medications

Details of AED treatment and concomitant medications related to the treatment of adverse reactions are to be collected from trial entry until trial completion .

The PI or delegated research team member should reassess AED treatment and concomitant medications related to the treatment of adverse reactions at each trial visit and record any changes on the applicable CRF.

9.2.7 Overdose

Suspected overdose with resulting signs and symptoms should be treated in accordance with the relevant SmPC and should be reported in accordance with section 10.

10 SAFETY REPORTING

For the purpose of safety (pharmacovigilance) reporting in CASTLE, an adverse event whose causal relationship to a participants treatment with one of the CASTLE IMPs (carbamazepine or levetiracetam) assessed by the investigator as "possibly", "probably", or "almost certainly" is classed as an Adverse Reaction (AR) and is reportable for CASTLE.

10.1 Terms and Definitions

The Medicines for Human Use (Clinical Trials) Regulations 2004 (SI 2004/1031) definitions apply:

Adverse Event (AE)
An "Adverse Event" (AE) is any untoward medical occurrence in a participant to whom a medicinal product has been administered, including occurrences that are not necessarily caused by or related to that product. Therefore an AE is any unfavourable or unintended change in the function (symptoms), structure (signs) or chemistry (laboratory data) in a participant to whom an IMP has been administered, including occurrences which are not necessarily caused by or related to that product.

Adverse Reaction (AR)
An "Adverse Reaction" (AR) is any untoward and unintended response in a participant to an IMP which is related to any dose administered to that participant.

Therefore an AR is any unfavourable or unintended change in the function (symptoms), structure (signs) or chemistry (laboratory data) in a subject that is related to any dose of an IMP administered to that participant.

Unexpected Adverse Reaction (UAR)
An "Unexpected Adverse Reaction" (UAR) is an adverse reaction the nature and severity of which is not consistent with the information about the medicinal product in question set out in:
- the case of a product with a marketing authorization, in the summary of product characteristics for that product;
- the case of any other IMP, in the investigator's brochure relating to the trial in question.

Serious Adverse Event (SAE), Serious Adverse Reaction (SAR) or Suspected Unexpected Serious Adverse Reaction (SUSAR)
A "Serious Adverse Event" (SAE), "Serious Adverse Reaction" (SAR) or "Suspected Unexpected Serious Adverse Reaction" (SUSAR) is any AE, AR or UAR, respectively, that:
- results in death
- is life-threatening* (participant at immediate risk of death)
- requires in-patient hospitalisation or prolongation of existing hospitalisation**
- results in persistent or significant disability or incapacity, or
- consists of a congenital anomaly or birth defect
- Other important medical events***

*'life-threatening' in the definition of 'serious' refers to an event in which the participant was at risk of death at the time of the event; it does not refer to an event that hypothetically might have caused death if it were more severe.

**Hospitalisation is defined as an inpatient admission, regardless of length of stay, even if the hospitalisation is a precautionary measure for continued observation. Hospitalisations for a pre-existing condition, including elective procedures that have not worsened, do not constitute an SAE.

***Other important medical events that may not result in death, be life-threatening, or require hospitalisation may be considered a serious adverse event/experience when, based upon appropriate medical judgment, they may jeopardise the participant and may require medical or surgical intervention to prevent one of the outcomes listed in this definition.

"Suspected Unexpected Serious Adverse Reaction (SUSAR)"
A "Suspected Unexpected Serious Adverse Reaction" (SUSAR) is an adverse reaction that is classed in nature as serious and which is not consistent with the information about the IMP in question, which in the case of a licensed product is set out in the SPC for that product, and in the case of any other IMP is set out in the IB relating to the trial in question.

"Reference Safety Information (RSI)"
The RSI in a trial is the information used for assessing whether an AR is expected. This is contained in the SmPC.

10.2 Notes on Adverse Event Inclusions and Exclusions

10.2.1 Inclusion of Related Adverse Events (Adverse Reactions)

Include the following AEs if causal relationship is "possibly", "probably", or "almost certainly" related to a participants treatment with one of the CASTLE IMPs (and therefore considered ARs):

- An exacerbation of a pre-existing illness
- An increase in frequency or intensity of a pre-existing episodic event/condition
- A condition (even though it may have been present prior to the start of the trial) detected after trial drug administration
- Continuous persistent disease or symptoms present at baseline that worsens following the administration of the trial treatment
- Laboratory abnormalities that require clinical intervention or further investigation (unless they are associated with an already reported clinical event).
- Abnormalities in physiological testing or physical examination that require further investigation or clinical intervention
- Injury or accidents

10.2.2 Exclusions

Do not include the following:

- AEs considered by the reporting investigator to be "unlikely" or "unrelated" to a participants treatment with one of the CASTLE IMPs
- Medical or surgical procedures - the condition which leads to the procedure is the AE
- Pre-existing disease or conditions present before treatment that do not worsen
- Situations where an untoward medical occurrence has occurred e.g. cosmetic elective surgery
- The disease being treated or associated symptoms/signs unless more severe than expected for the patient's condition

10.2.3 Notification of Deaths

All deaths that occur during the protocol specified safety event reporting period regardless of relationship to the CASTLE IMPs, must be recorded. All deaths should be reported on a SAE CRF securely sent via email to CTRC within 24 hours.

Death should be considered an outcome and not a distinct event. The event or condition that caused or contributed to the fatal outcome should be recorded as the single medical concept on the SAE CRF. Generally only one such event should be reported. The term “sudden death” should only be used for the occurrence of an abrupt and unexpected death due to presumed cardiac causes in a participant with or without pre-existing heart disease, within 1 hour of the onset of acute symptoms or, in the case of an unwitnessed death, within 24 hours after the participant was last seen alive and stable. If the cause of death is unknown and cannot be ascertained at the time of reporting, “unexplained death” should be recorded on the SAE CRF. If the cause of death subsequently becomes available (e.g., after autopsy), “unexplained death” should be replaced by the established cause of death.

10.2.4 Reporting of Pregnancy

Any pregnancy which occurs during the trial should be reported to the CTRC using a Pregnancy CRF within 24 hours of the centre becoming aware of its occurrence. All pregnancies that occur during treatment need to be followed up until after the outcome using the Pregnancy CRF.

Any serious safety event (SAE, SAR or SUSAR) experienced during pregnancy (or at birth, e.g. birth defect) must be reported on the SAE CRF as appropriate (see section 10.7). The investigator should contact the participant to discuss the risks of continuing with the pregnancy and the possible effect to the fetus. Appropriate obstetric care should be arranged.

CTRC will inform KHP CTO of any pregnancies that occur in the trial within 24 hours of CTRC becoming aware. The outcome of pregnancies will be monitored by the IDSMC and CTRC.

10.2.5 Reporting of Overdoses

All overdoses regardless of causality or seriousness should be reported using an SAE form to the CTRC immediately (within 24 hours) for assessment under the advice of the CI.

10.3 Notes on Severity / Grading of Adverse Events

The assignment of the severity/grading should be made by the investigator responsible for the care of the participant (or any medically qualified person delegated the duty on the delegation log) using the definitions below.

Regardless of the classification of a reaction as serious or not, its severity must be assessed according to medical criteria alone using the following categories:

Mild: does not interfere with routine activities
Moderate: interferes with routine activities
Severe: impossible to perform routine activities

A distinction is drawn between serious and severe ARs. Severity is a measure of intensity (see above) whereas seriousness is defined using the criteria in section 10.1, hence, a severe AR need not necessarily be a SAR.

10.4 Relationship to Trial Treatment

The assignment of the causality should be made by the investigator responsible for the care of the participant (or any medically qualified person) delegated the duty on the delegation log) using the definitions in Table 4: Definitions of Causality.

If any doubt about the causality exists the local investigator should inform the CTRC who will notify the Chief Investigator (CI). In the case of discrepant views on causality between the investigator and others, the MHRA will be informed of both points of view.

Table 4: Definitions of Causality

Relationship	Description
**Unrelated**	There is no evidence of any causal relationship. N.B. An alternative cause for the AE should be given.
**Unlikely**	There is little evidence to suggest there is a causal relationship (e.g. the event did not occur within a reasonable time after administration of the trial medication). There is another reasonable explanation for the event (e.g. the participant's clinical condition, other concomitant treatment).
**Possibly**	There is some evidence to suggest a causal relationship (e.g. because the event occurs within a reasonable time after administration of the trial medication). However, the influence of other factors may have contributed to the event (e.g. the participant's clinical condition, other concomitant treatments).
**Probably**	There is evidence to suggest a causal relationship and the influence of other factors is unlikely.
**Almost certainly**	There is clear evidence to suggest a causal relationship and other possible contributing factors can be ruled out.

10.5 Expectedness

It is not a regulatory requirement for a reporting physician to provide their opinion of expectedness. Therefore, the reporting physician at the research centres will not be asked to make the assessment of expectedness. The assessment of expectedness will be made by the CI (or designated other) using the current approved RSI in CASTLE (see section 9.2)

10.6 Follow-up After Adverse Reactions

All safety events should be followed until satisfactory resolution or until the investigator responsible for the care of the participant deems the event to be chronic or the participant to be stable.

When reporting SAEs, SARs and SUSARs the investigator responsible for the care of the participant must provide information relating to event outcomes in accordance with the SAE CRF.

10.7 Reporting Procedures

Depending on the nature of the event the reporting procedures below should be followed. Any questions concerning adverse event reporting should be directed to the CTRC in the first instance. A flowchart is given (Figure 2: Flowchart for Reporting Requirements of Safety Events) to aid in determining reporting requirements.

10.7.1 Non-serious and unrelated AEs

Non-serious and unrelated AEs (causality relationship to a CASTLE IMP of “unrelated” or “unlikely”) will not be reported as part of the CASTLE trial.

10.7.2 Non-serious ARs

All non-serious safety events which are assessed as “possibly”, probably” or “almost certainly” related to a CASTLE IMP (ARs) will be reported as part of the CASTLE trial. Non-serious ARs should be recorded by centre research teams on an AR CRF, which should be transmitted to the CTRC within 7 days of the research team becoming aware of the AR.

10.7.3 Serious safety events (SAEs/SARs/SUSARs)

All serious safety events regardless of causality (i.e. SAEs, SARs and SUSARs) must be reported to the CTRC on a SAE CRF within 24 hours of the local centre research team becoming aware of the event. The SAE CRF asks for the nature of event, date of onset, severity, corrective therapies given, outcome and causality. The responsible investigator should sign the causality of the event. Additional information should be sent within 5 days if the reaction has not resolved at the time of reporting.

Upon receipt of a SAE CRF, the CTRC will forward it to the CI (or an agreed delegate) to provide an assessment of expectedness and provide medical review of the event.

The CTRC will pass on any SUSARs to KHP CTO who will notify the MHRA of all SUSARs occurring during the study. The CTRC will notify REC of all SUSARs occurring during the study. The MHRA and REC will be notified according to the following timelines; fatal and life-threatening within 7 days of notification and non-life threatening within 15 days. All centre PIs will be informed of all SUSARs occurring throughout the trial. Local investigators should report any SUSARs and /or SAEs as required locally.

Active monitoring for serious safety events (SAEs/SARs/SUSARs) will be performed for each participant from the time of randomisation until the final follow up visit; any serious safety events (SAEs/SARs/SUSARs) which the centre research teams become aware of after the final follow-up visits must be reported to CTRC within 24 hours.

Figure 2: Flowchart for Reporting Requirements of Safety Events

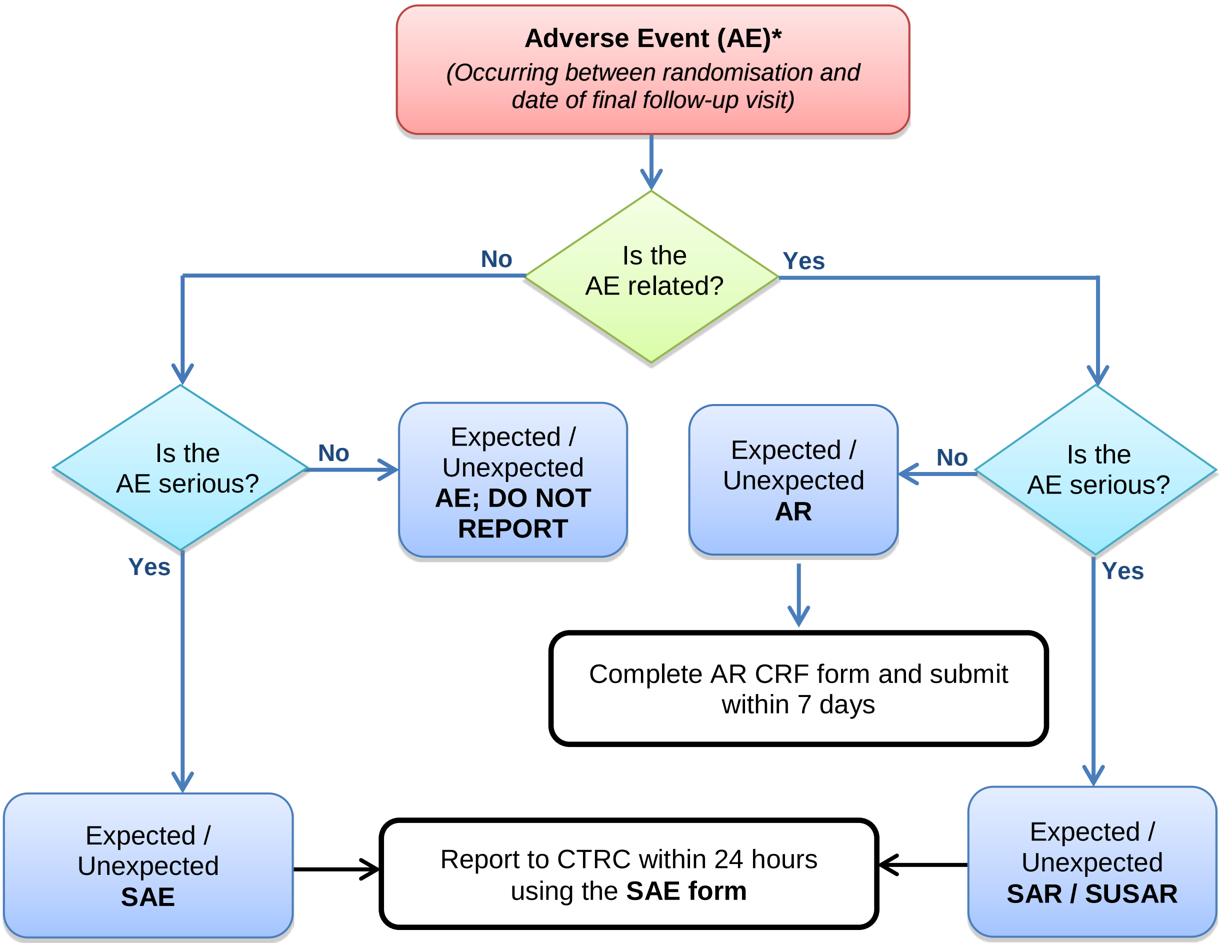

**If an adverse reaction occurs outside of this time window and the local investigator feels that the event is related to the trial treatment administered, the above process should still be followed*

10.8 Responsibilities – Investigator

The Investigator is responsible for active monitoring and reporting of all non-serious related safety events (i.e. ARs) that are observed or reported for participants from randomisation until their final follow-up visit. The Investigator is also responsible for active monitoring and reporting of all serious safety events and SAEs, regardless of their relationship to trial product (i.e. SAEs, SARs, SUSARs) for participants from randomisation until their final follow-up visit; after the final visit, the Investigator no longer needs to actively monitor for serious safety events, but is responsible for ensuring any which the research team become aware of are reported for the trial. Reporting timelines are described in section 10.7.

Minimum information required for reporting serious adverse events (SAEs/SARs/SUSARs):

- Valid EudraCT number
- Sponsor trial number
- One identifiable coded subject (randomisation number)
- One identifiable reporter (name of PI or appropriate delegated individual)
- One safety event
- One suspect IMP (including active substance name-code)
- A seriousness assessment
- A causality assessment

Process for reporting SAEs:

i. The SAE CRF should be completed by a designated investigator, a physician named on the 'Delegation of Authority and Signature Log' as responsible for reporting SAEs and making trial related medical decisions. The investigator should assess the SAE for the likelihood that it is a response to a CASTLE IMP. In the absence of the designated investigator the form should be completed and signed by an alternative member of the research centre trial team and submitted to the CTRC. As soon as possible thereafter the responsible investigator should check the SAE CRF, make amendments as appropriate, sign and re-send to the CTRC. The initial report shall be followed by detailed follow-up reports as appropriate.

ii. The SAE CRF must be submitted within 24 hours of the local research team becoming aware of the event to the CTRC by email.

iii. When submitting an SAE CRF to the CTRC, research centres should also telephone the appropriate trial co-ordinator/data manager on telephone number **0151 795 8774** to advise that an SAE report has been submitted.

iv. The participant **must** be identified by randomisation number, date of birth and initials only. The participant's name **should not** be used on any correspondence.

v. The responsible investigator must **notify** their R&D department of the event (as per standard local governance procedures).

vi. The participant must be followed-up until clinical recovery is complete and laboratory results have returned to normal, or until the event has stabilised. Follow-up may continue after completion of protocol treatment if necessary.

vii. Follow-up information is noted on another SAE CRF by ticking the box marked 'follow-up' and securely sending via email to the CTRC as information becomes available. Extra, annotated information and/or copies of test results may be provided separately.

10.9 Responsibilities – CTRC and KHP CTO

The CTRC is undertaking duties delegated by the trial co-sponsors (King's College Hospital NHS Foundation Trust and King's College London) and will forward completed SAE reports of SUSARs to KHP CTO. KHP CTO is responsible for the reporting of SUSARs and other SARs to the MHRA and the CTRC is responsible for the reporting to REC as follows:

- SUSARs which are fatal or life-threatening must be reported not later than 7 days after the CTRC is first aware of the reaction. Any additional relevant information must be reported within a further 8 days.
- SUSARs that are not fatal or life-threatening must be reported within 15 days of the CTRC first becoming aware of the reaction.
- A list of all SARs (expected and unexpected) must be reported annually.

It is recommended that the following safety issues should also be reported in an expedited fashion:

- An increase in the rate of occurrence or a qualitative change of an expected SAR, which is judged to be clinically important;
- New events related to the conduct of the trial or the development of the IMPs and likely to affect the safety of the participants, such as:
    a. An SAE which could be associated with the trial procedures and which could modify the conduct of the trial;

   b. A significant hazard to the participant population, such as lack of efficacy of an IMP used for the treatment of a life-threatening disease;
   c. A major safety finding from a newly completed animal trial (such as carcinogenicity);
   d. Any anticipated end or temporary halt of a trial for safety reasons and conducted with the same IMP in another country by the same sponsor;
- Recommendations of the IDSMC, if any, where relevant for the safety of the participants.

Staff at the CTRC will liaise with the CI (or designated other specified in the protocol) who will review all SAEs received for seriousness and causality, and provide expectedness assessments. Safety events that are SUSARs will be identified and reported to MHRA and REC. The causality assessment given by the local investigator at the hospital cannot be overruled and in the case of disagreement, both opinions will be provided with the report.

The PIs at all institutions participating in the trial will be notified of any SUSARs.

Patient safety incidents that take place in the course of research should be reported to the national reporting and learning system (NRLS) by each participating NHS Trust in accordance with local reporting procedures.

10.9.1 Safety reports

Safety reports will be generated during the course of the trial which allows for monitoring of SAE and AR reporting rates across centres. The CTRC will send annual developmental safety update reports (DSURs) containing a list of all SARs to the REC and KHP CTO will be responsible for sending this report to the MHRA. Any concerns raised by the IDSMC or inconsistencies noted at a given centre may prompt additional training at centres, with the potential for the CTRC/KHP CTO to carry out centre visits if there is suspicion of unreported ARs or SAEs in participant case notes. Additional training will also be provided if unacceptable delay in safety reporting timelines. If any safety reports identify issues that have implications for the safety of trial participants, the PIs at all institutions participating in the trial will be notified.

10.9.2 Urgent Safety Measures (USMs)

An USM is a procedure not defined by the protocol, which is put in place prior to authorisation by the MHRA and REC in order to protect clinical trial participants from any immediate hazard to their health and safety.

The KHP CTO will notify the MHRA and CTRC will notify REC immediately and, in any event, within 3 days that such a measure has been taken and the reasons why it has been taken. The initial notification to the MHRA will be by telephone (ideally within 24 hours) and a notice in writing will be sent within 3 days, setting out the reasons for the USM and the plan for further action. After discussion with the MHRA and REC, further action will be agreed, which may include submission of a substantial amendment, a temporary halt, or permanent termination of the trial.

If the trial is temporarily halted it may not recommence until authorised to do so by the MHRA and REC. If the trial is permanently terminated before the date specified for its conclusion (in the original applications to MHRA and REC), the co-sponsors should notify the MHRA and REC within 15 days of the date of termination by submitting the formal End of Trial Notification.

10.10 Contact Details and Out-of-hours Medical Cover

CASTLE participants are being recruited within secondary or tertiary centres for paediatrics in the UK and the IMPs allocated in CASTLE are used either within their licence or are established practice for the indication of RE. Following randomisation, a participant's care pathway and treatment is in line with usual care in the NHS and ongoing treatment decisions are at the discretion of the treating physician.

As such, emergency and out-of-hours medical care will be in line with usual NHS arrangements and local standard practice and no special provision is required for CASTLE participants.

All participants will be issued with a copy of the signed information sheet and consent/assent forms, which includes information about the patient's participation in the CASTLE trial and details of the research team locally who may be contacted if needed. During office hours, the CI or delegate are able to provide medical advice in relation to children recruited to CASTLE should this be necessary. They can be contacted via the CASTLE trial coordinator at the CTRC.

Tel: 00 44 (0) 151 795 8774
E-mail: castle.rct@liverpool.ac.uk

11 STATISTICAL CONSIDERATIONS

11.1 Introduction

A separate and full Statistical Analysis Plan (SAP) will be developed prior to the final analysis of the trial. The SAP will be agreed by the TSC before being sent to the IDSMC for comment and approval.

11.2 Method of Randomisation

Randomisation will be performed via a web based tool accessed by research team at site. This system is generated centrally by the CTRC using a computer algorithm concealed from the investigators and research teams/trial management group (see section 7.5). In order to balance the groups, minimisation for variables believed to influence disease outcome and end points will be built into the randomisation algorithm.

11.3 Sample Size calculation

11.3.1 Drug Intervention

The primary outcome is time to 6-month seizure remission. A 5% two-sided significance level is used for each of two primary comparisons of interest: carbamazepine versus active monitoring and levetiracetam versus active monitoring. We have obtained individual patient data from the trial sponsor of the German HEAD study (30) to provide an estimated probability of being without remission on placebo at 6 months of 0.57. With 97 per group we would have 80% power to detect a reduction from 0.57 to 0.37 (Hazard Ratio=0.57). Inflating sample size to allow for 12% attrition requires total n=330.

11.3.2 Sleep Intervention

The primary outcome is CSHQ score at 3 months. Assuming that the mean CSHQ score in the 'no sleep intervention' group is 48.25 (31), a reduction to 41.1 (difference of 7.15) to be in line with normal controls (31) would require 38 per group with 90% power (assuming SD=8.9 (31). Recognising that we might not reduce the score to be completely in line with healthy controls 50 per group would be required to have 90% power to detect a reduction to 42 (assuming SD=8.9) (31).

11.3.3 Feasibility (attaining recruitment targets)

We will recruit 330 participants in total. This will give 80% power to address the aims of the drug intervention component and over 90% power to address the aims of the sleep intervention component.

11.4 Interim Monitoring and Analyses

We plan to undertake an internal "pilot" trial to assess recruitment and consent during the first 9 months of recruitment. The IDSMC will review the internal pilot data and make recommendations to the TSC in respect of trial continuation and possible adjusting methods for recruitment and follow-up optimisation for the remaining timeline. By the end of month 9 we expect to have opened all 22 centres and randomised 28 participants (accounting for a staggered opening of centres using a similar pattern to SANAD-II (EudraCT number 2012-001884-64)). The following criteria will be considered:

- If recruitment is 80-100% of the planned recruitment figure (28 participants), the trial will progress.

- If recruitment is 50-79% of the planned figure, the trial will progress following review of screening logs and protocol and once barriers to achieve adequate recruitment are addressed.
- Should recruitment be <50%, it is not expected the trial will progress; the decision will be made by the TSC in conjunction with the co-sponsors.

We will also assess follow-up rate, treatment failure rate, and completeness of data for primary outcomes. Although the primary aim of the trial is to compare treatment policies as applied in clinical practice (and hence an intention to treat (ITT) analysis), we will be particularly interested in monitoring the percentage of participants switching from no AED to AED treatment to determine the likely impact on power for the primary analysis.

Formal interim analyses of the accumulating data will be performed at regular intervals (at least annually) for review by an IDSMC. These analyses will be performed at the CTRC. The IDSMC will be asked to give advice on whether the accumulated data from the trial, together with results from other relevant trials, justifies continuing recruitment of further participants or further follow-up. A decision to discontinue recruitment, in all participants or in selected subgroups will be made only if the result is likely to convince a broad range of clinicians including participants in the trial and the general clinical community.

11.5 Analysis Plan

All primary analyses will be on an ITT basis including all randomised participants retained in their randomised treatment groups. Secondary compliance adjusted causal effect analyses will also be undertaken to assess the robustness of ITT analyses.

11.5.1 Primary and Secondary Outcomes

The primary outcomes are time to 6-month remission from seizures (for the hypothesis that carbamazepine or levetiracetam are superior to no AED) and change in CSHQ at 3 months (for the hypothesis that sleep behaviour intervention will be superior to standard care).

Following the principles discussed by Montgomery *et al.* (32), two separate regression analyses, one for each primary outcome, will be undertaken.

The interval (in days) from randomisation to occurrence of a 6-month remission will be summarised by Kaplan-Meier curves for each treatment group. A Cox proportional hazards model will be fitted with factors included for AED (CBZ, LEV *vs* no AED); sleep (intervention *vs* no intervention); baseline variables. The adjusted Hazard Ratio main effects of CBZ or LEV compared to no AED will be estimated and presented with a p-value and 95% confidence interval.

For the second primary outcome CSHQ, a linear regression model will be fitted with factors included for AED (CBZ, LEV *vs* no AED); sleep (intervention *vs* no intervention); baseline variables, including baseline CSHQ. The adjusted mean difference main effect of sleep intervention compared to no sleep intervention will be estimated and presented with a 95% confidence interval.

The impact of centre effect on the treatment comparison will be investigated by considering both fixed and random effects models. When analysing the data using the fixed effect approach, the centre effect is to be modelled by a series of dummy variables. However, due to concern regarding the number of dummy variables required, a random effects analysis will also be considered.

The primary analysis will address the principal research question focusing on treatment main effects. In secondary analyses the interaction between AED and sleep intervention will be investigated, recognizing that the precision of the estimates of interaction is very likely to be too poor for large effects to be ruled out.

All analyses will be reported in accordance with the CONSORT checklist (33) and regardless of statistical significance. There will be no correction for multiple comparisons (34).

A similar strategy will be used for the analysis of secondary time to event outcomes. For time to treatment failure, further analysis will be undertaken to assess the two main reasons for treatment failure - inadequate seizure control and unacceptable adverse effects. To allow for possible dependence between the different withdrawal risks, cumulative incidence analyses will be presented (34).

The Haybittle-Peto approach will be employed for each interim analysis, with 99.9% confidence intervals calculated for interim analysis effect estimates. The final analysis will be undertaken at the end of the trial when all participants have a minimum 12 months follow-up data and 95% confidence intervals will be calculated.

QOL data will be analysed longitudinally to explore between treatments changes in scale scores over time, taking account of baseline QOL.

Qualitative interview data will be analysed using an interpretive, reflexive and conceptual analytical approach. Data will be thematically analysed in discrete sets (e.g., the trial group and the non-trial groups, different treatments/management, different responses/experiences), and parents/primary carers' and children's transcripts will be analysed separately before considering each dyad and prior to synthesis of all the data.

For the analysis of safety events, all participants who received any amount of each trial drug will be included in the safety analysis dataset in the treatment group they actually received. All safety events reported by the clinical investigators will be presented, identified by treatment group. Safety events will be grouped according to a pre-specified coding system and tabulated. The number (and percentage) of participants experiencing each safety event, and the number (and percentage) of occurrences of each safety event will be presented. No formal statistical testing will be undertaken.

11.5.2 Health Economic Evaluation

The primary (cost utility) analysis will consider (patient) QALYs and costs from the perspective of the NHS and PSS. The incremental analysis will be based on the mean costs and QALYs for each treatment group, taking into consideration the factorial trial design (35). The Health Economic Analysis Plan (HEAP) (agreed prior to the analysis and produced by Bangor University), will define the analytic steps to be undertaken. Bangor University will impute data assumed missing at random using multiple imputation by chained equations as will be specified in the HEAP.

Sensitivity analyses will be conducted to test the robustness of findings. Bangor University will use such analyses based on the observed distributions of outcome and costs to test whether, and to what extent, the incremental cost-effectiveness ratios are sensitive to key assumptions in the analysis (e.g. unit prices, different utility estimates CHU-9D vs. EQ-5D-Y).

Bangor University will present alternative scenarios broadening the cost perspective, and considering a range of outcomes, including parental QALYs. These will be presented in a cost consequence analysis, and include indirect costs, such as educational attainment and loss of productivity, which will be valued by reference to published sources. Inclusion of spill-over disutility (impact on parents/primary carers' utility) will be based on the NICE reference case specification that all QALYs being of equal weight, and calculated assuming additive effects.

The joint uncertainty in costs and QALYs will be addressed through application of bootstrapping and estimation of cost-effectiveness acceptability curves (36). The cost-effectiveness frontier will allow for graphical representation of the interventions most likely to be cost-effective according to different thresholds of cost-effectiveness. The economic findings will be reported according to the CHEERS guideline (37).

12 REGULATORY AND ETHICAL APPROVALS

12.1 Statement of Compliance

The trial will be carried out in accordance with Good Clinical Practice (ICH GCP) and the Declaration of Helsinki (1996).

12.2 UK Policy Framework for Health and Social Care Research Regulatory Approval

This trial falls within the remit of the EU Directive 2001/20/EC, transposed into UK law as the UK Statutory Instrument 2004 No 1031: Medicines for Human Use (Clinical Trials) Regulations 2004 as amended. This trial has been registered with the MHRA and has been granted a Clinical Trial Authorisation (CTA). The EudraCT reference is 2018-003893-29.

12.3 Ethical Considerations

The specific ethical issues relating to participation in this trial are considered to be:

Informed consent in a paediatric population: The appropriate adult providing consent on behalf of the minor participant will have an interview with the investigator, or a delegated member of the investigating team, during which opportunity will be given to understand the objectives, risks and inconveniences of the trial and the conditions under which it is to be conducted. They will be provided with written information and contact details of a member of the research team at the centre, from whom further information about the trial may be obtained, and will be made aware of their right to withdraw the child from the trial at any time without the child or family being subject to any detriment in the child's treatment. Children will receive information, according to their capacity of understanding, about the trial and its risks and benefits and their assent will be sought and obtained, where appropriate (≥7 years old). Given the participatory approach of the qualitative sub-study, particular attention will be paid to ensuring that the children and parents/primary carers fully understand their involvement in the qualitative component. The approach and materials involved will be tailored, and wording used in the interview contexts will be developed, tailored and adjusted, in consultation with the PPI panel.

12.4 Ethical Approval

Prior to the trial being initiated at CTRC, a favourable opinion will be obtained from a REC and global governance approval from the Health Research Authority (HRA). Prior to opening a centre to recruitment, CTRC will ensure that local governance approval has been obtained: this will be "Capacity & Capability" Confirmation or R&D approval depending on the country of issue. A copy of localised versions of the participant/parent/appropriate adult information and consent/assent form should be forwarded to CTRC before the centre is initiated and patients recruited.

12.5 Protocol Deviation and Serious Breaches

A breach of the protocol or GCP is 'serious' if it meets the regulatory definition of being "likely to affect to a significant degree the safety or physical or mental integrity of the trial participants, or the scientific value of the trial". All serious breaches of GCP or protocol will be reported to the MHRA by KHP CTO and to REC by CTRC in an expedited manner.

If any persons involved in the conduct of the trial become aware of a potential serious breach, they must immediately report this to the CTRC who will in turn notify the co-sponsors. The co-sponsors will assess the breach and determine if it meets the criteria of a ‘serious’ breach of GCP or protocol and therefore requires expedited reporting to the MHRA and REC.

In determining whether or not the breach is likely to affect to a significant degree the safety, physical or mental integrity of participants, the co-sponsors may seek advice from medical expert members of the TMG and/or of the independent oversight committees (IDSMC and TSC). In determining whether or not the breach is likely to significantly affect the scientific value of the trial, the co-sponsors may seek advice from the trial statistician. However, the co-sponsors retain responsibility for the assessment of whether or not a breach meets the definition of ‘serious’ and is subject to expedited reporting to MHRA and REC.

Breaches confirmed as ‘serious’ will be reported to the MHRA by KHP CTO and to REC by CTRC within 7 days and notified to the TMG, IDSMC and TSC at their next meeting.

Any requests for additional information from the co-sponsors, TMG, TSC, IDSMC, REC or MHRA, will be promptly actioned by the relevant member(s) of the research team and open communication will be maintained to ensure appropriate corrective actions are taken and documented.

Incidence of protocol non-compliance are recorded as protocol deviations, the incidence of which is monitored and reported to trial oversight committees.

12.6 Trial Discontinuation

In the event that the trial is discontinued, participants will be treated according to usual standard clinical care. The process for participants who withdraw early from trial treatment or from the trial completely is described in section 8.5.

13 DATA MANAGEMENT AND TRIAL MONITORING

Details of the monitoring to be carried out for the CASTLE trial are included in the CASTLE trial monitoring plan.

Trial oversight committees related to the monitoring of the trial are detailed in section 15.4.

13.1 Risk Assessment

A risk assessment is performed for each trial coordinated by the CTRC to determine the level and type of monitoring required for specific hazards. The type of trial monitoring should be specific to the individual trial and can take the form of routine on-site visits or central monitoring (with triggered on-site visits only).

In accordance with the CTRC processes, CASTLE will undergo a risk assessment, completed in partnership between:

- Representatives of the trial co-sponsors
- Chief Investigator
- Trial coordinator and supervising senior trial manager
- Trial statistician and supervising statistician
- CTRC Director

13.2 Source Documents

***Source data**: All information in original records and certified copies of original records of clinical findings, observations, or other activities in a clinical trial necessary for the reconstruction and evaluation of the trial. Source data are contained in source documents (original records or certified copies; audio recordings; interview transcripts; drawings and diary entries). (ICH E6, 1.51).*

***Source documents**: Original documents, data, and records (e.g., hospital records, clinical and office charts, laboratory notes, memoranda, participants diaries or evaluation checklists, pharmacy dispensing records, recorded data from automated instruments, copies or transcriptions certified after verification as being accurate copies, microfiches, photographic negatives, microfilm or magnetic media, x-rays, participant files, and records kept at the pharmacy, at the laboratories and at medico-technical departments involved in the clinical trial). (ICH E6, 1.52).*

In order to resolve possible discrepancies between information appearing in the CRF and any other patient related documents, it is important to know what constitutes the source document and therefore the source data for all information in the CRF.

The CRF will be considered the source document for data where no prior record exists and which is recorded directly in the CRF. A CASTLE source document list will be completed by each centre to confirm the source documents.

Date(s) of conducting informed consent (plus assent where appropriate and if sought/reason if not sought) process including date of provision of patient information, type and version of information sheets used, confirmation of full eligibility, randomisation number and the fact that the patient is participating in a clinical trial (including possible treatment arms) should be added to the patient’s medical record chronologically.

13.3 Data Capture Methods

Clinical data capture will be in the form of paper copies of CRFs that will be returned as an on-going process from each centre to the CTRC. Patient/parent reported data will be collected directly on paper at each outpatient visit with the exception of CANTAB, which will be collected on iPads at the centre.

All data transferred from centres to CTRC will be pseudonymised and must be sent separately to documents containing identifiers (e.g. consent/assent forms containing names) in order to preserve pseudonymisation and maintain confidentiality.

Questionnaires – Patient and primary carer questionnaires completed on paper are a source document and **centres should securely** store them and retain a copy before mailing originals to CTRC at their earliest convenience after completion.

The CANTAB application will be clearly set up to ensure correct data entry and both the iPad and the application will be password protected. Subject data is only stored on the tablet until the testing session has finished and the iPad is connected to the internet. CANTAB data is then securely uploaded and stored securely online. A summary of data will be securely downloaded by authorised CTRC users, who will perform the analysis.

The contacts database will securely store personal and contact details for participants randomised to PBS and/or selected for actigraphy. This system will be linked to the randomisation system, detect which participants have been randomised to PBS and/or selected for actigraphy and alert the sleep team at Oxford Brookes University. For participants randomised to the PBS arm, the contacts database will also use the participant's details to create their personal online PBS account and email them their account details.

Actigraphy data will be obtained from the actigraphy watches by the sleep research team at Oxford Brookes University, who will then use the information recorded in the sleep logs collected by the parent/primary carer (with or without participant) to identify the appropriate periods for sleep analysis. Data will finally be transferred by secure electronic means to the CTRC.

The e-learning PBS user data directly collected online from parents will be securely stored at CTRC.

The qualitative sub-study will take place across three time points, and will use in-depth, semi-structured, face-to-face or remote interviews, and prioritization activities, with participants. Across all time points participants' (parents/primary carers and children) experiences of dialogue with clinicians about their concerns, and their decisions and choices will be explored. Interview questions and materials will be developed in consultation with PPI panel. All interviews will be audio recorded and transcribed. Audio recordings will be retained in a secure environment until transcripts have been finalised.

13.4 Monitoring

13.4.1 Central Monitoring

Data received on CRFs at CTRC will be checked for missing or unusual values (range checks) and checked for consistency within participants over time. Any suspect data will be returned to the centre in the form of data queries. Data query forms will be produced at the CTRC from the trial database and sent either electronically or through the post to a named individual (as listed on the centre

delegation log). Centres will respond to the queries providing an explanation/resolution to the discrepancies and return the data query forms to CTRC. The forms will then be filed along with the appropriate CRFs and the appropriate corrections made on the database. There are a number of monitoring features in place at the CTRC to ensure reliability and validity of the trial data, to be detailed in the trial monitoring plan.

13.4.2 Clinical Centre Monitoring

In order to perform their role effectively, CTRC personnel (e.g. trial coordinator or monitor or data manager) and persons involved in quality assurance and inspection may need direct access to source data, e.g. patient medical records, laboratory reports, appointment books, etc. Since this affects the participants' confidentiality, this fact is included on the PISC form.

13.5 Confidentiality

All persons involved in the trial have a duty to preserve the confidentiality of participants taking part in it.

Individual participant medical information obtained as a result of this trial is considered confidential and disclosure to third parties is prohibited with the exceptions noted below:

- CRFs will be pseudo-anonymised (labelled with the participant's initials and unique trial randomisation number). Medical information may be given to the participant's medical team and all appropriate medical personnel responsible for the participant's welfare.
- Actigraphy data will be pseudo-anonymised and collected by the sleep intervention team at Oxford Brooks University.
- Online PBS data will be pseudo-anonymised and collected by CTRC.
- CANTAB data will be pseudo-anonymised and collected from a centralised online server that can only be accessed by authorised users.
- HES and PLICS data obtained from NHS Digital (or national equivalent) and hospital finance departments will be pseudo-anonymised using the trial randomisation number and securely sent to Bangor University for analysis.

The CTRC will be undertaking activities requiring the transfer of identifiable data:

- Verification that appropriate informed consent/assent is obtained will be enabled by the provision of copies of participant's signed informed consent/assent forms to the CTRC by recruiting centres, which requires that name data will be transferred to the CTRC.
- Identifiable data will also be transferred to CTRC through the contacts database when recruiting centres fill out the participant and parent/primary carer's details.
- CTRC will securely send to the qualitative sub-study team identifiable data of those participants or trial decliners that have agreed to it using DatAnywhere.

This transfer of identifiable data is disclosed in the PISC. The CTRC will preserve the confidentiality of participants and appropriate adults providing consent on their behalf and the University of Liverpool is registered with the Information Commissioners Office (ICO).

Any identifiable data collected during the course of the qualitative sub-study will be managed according to appropriate data protection and research governance measures.

13.6 Quality Assurance (QA) and Control (QC)

Quality Assurance (QA) includes all the planned and systematic actions established to ensure the trial is performed and data generated, documented/recorded and reported in compliance with applicable regulatory requirements. Quality Control (QC) includes the operational techniques and activities done within the QA system to verify that the requirements for quality of the trial-related activities are fulfilled e.g. state what clinical centre monitoring (and audit) is planned, if any. In accordance with the monitoring plan centre visits will or will not be conducted and source verification performed if indicated to be require as a result of central monitoring processes. To this end:

- The trial coordinator at the CTRC will verify appropriate approvals are in place prior to initiation of a centre and the relevant personnel have attended trial specific training. A greenlight checklist will verify all approvals are in place prior to trial initiation at CTRC and the individual centre.
- The TMG will determine the minimum key staff required to be recorded on the delegation log in order for the centre to be eligible to be initiated.
- Data will be evaluated for compliance with protocol.
- The trial will be conducted in accordance with procedures identified in the protocol.
- Independent oversight of the trial will be provided by the IDSMC and independent members of the TSC.
- The types of materials to be reviewed, who is responsible, and the schedule for reviews may be specified or referenced in other documents
- Types and mechanisms of training of staff for the trial should be specified.
- The PI and other key staff from each centre will attend centre initiation training, coordinated by the CTRC, which will incorporate elements of trial-specific training necessary to fulfil the requirements of the protocol.
- The TMG is to monitor screening, randomisation and consent rates between centres.
- The process for consent, recruitment and randomisation will be evaluated for compliance with the protocol.
- Data quality checks and monitoring procedures will be undertaken in line with the trial data management plan and data entry and cleaning manual.

13.7 Records Retention

All trial documents (except raw HES data from NHS digital that will only be retained for 1 year) will be retained for 25 years from the End of Trial.

The PI at each investigational centre must make arrangements to store the essential trial documents, (as defined in Essential Documents for the Conduct of a Clinical Trial (ICH E6, Guideline for Good Clinical Practice)) including the ISF, until the CTRC informs the investigator that the documents are no longer to be retained.

The PI is responsible for archiving of all relevant source documents so that the trial data can be compared against source data after completion of the trial (e.g. in case of inspection from authorities).

The PI is required to ensure the continued storage of the documents, even if the investigator, for example, leaves the clinic/practice or retires before the end of required storage period. Delegation must be documented in writing.

KHP CTO undertakes to store originally completed CRFs for the same period, except for source documents pertaining to the individual investigational centre, which are kept by the investigator only. KHP CTO will archive the documents in compliance with the principles of GCP. All electronic CRFs and trial data will be archived onto an appropriate media for long-term accessible storage.

All other persons and organisations involved in the trial will be responsible for storing the parts of the TMF during the trial relevant to their delegated duties (e.g., qualitative sub-study team, health economics, etc. At the end of the trial, the co-sponsors will have the responsibility to archive the entire TMF.

14 INDEMNITY

CASTLE is co-sponsored by King’s College Hospital NHS Foundation Trust and King’s College London and co-ordinated by the CTRC in the University of Liverpool.

The lead sponsor, King's College London, will take primary responsibility for ensuring that the design of the study meets appropriate standards and that arrangements are in place to ensure appropriate conduct and reporting. King's College London also provides cover under it's No Fault Compensation Insurance, which provides for payment of damages or compensation in respect of any claim made by a research subject for bodily injury arising out of participation in a clinical trial or healthy volunteer study (with certain restrictions). The co-sponsor, King's College Hospital, takes ultimate responsibility for arranging the initiation and management of this research, and will take responsibility for ensuring that appropriate standards, conduct and reporting are adhered to regarding its facilities and staff involved with the project. King’s College Hospital will also undertake the governance review for the project and provide cover for clinical negligence by any of its staff in undertaking the research, under the CNST scheme managed by the NHS LA.

Clinical negligence is defined as:
“A breach of duty of care by members of the health care professions employed by NHS bodies or by others consequent on decisions or judgments made by members of those professions acting in their professional capacity in the course of their employment, and which are admitted as negligent by the employer or are determined as such through the legal process”.

15 ROLES AND RESPONSIBILITIES

15.1 Role of Trial Co-Sponsors and Trial Funder

King's College Hospital NHS Foundation Trust and King's College London are the co-sponsoring organisations and are legally responsible for the trial.

The co-sponsors delegate specific roles to the CI, KHP CTO, co-applicants and CTRC with regards to trial design, conduct, data analysis and interpretation, manuscript writing, and dissemination of results. Delegations are described in contracts.

15.2 Funding and Support in Kind

Funder(s)	Financial and Non-financial Support Given	Role
NIHR Programme Grant	Financial Support:<br>NIHR provides on-going help and support to the CI to ensure that the programme progresses smoothly. Trial design, conduct, data analysis and interpretation, manuscript writing and dissemination of results are the responsibility of co-sponsors and their delegates, though the funder will monitor progress against key milestones via the submission of regular progress reports.	Funder

15.3 Protocol Contributors

Name	Affiliations	Contribution to protocol
Prof Deb Pal	King's College London	Co-author
Prof Paul Gringras	Evelina London Children's Hospital	Co-author
Amber Collingwood	King's College London	Co-author
Prof Catrin Tudur Smith	University of Liverpool	Co-author
Helen Hickey	University of Liverpool	Co-author
Catherine Spowart	University of Liverpool	Co-author
Dr Agnes Tort Piella	University of Liverpool	Co-author
Dr Chris Morris	University of Exeter	Co-author
Prof Bernie Carter	Edgehill University	Co-author
Prof Lucy Bray	Edgehill University	Co-author
Dr Luci Wiggs	Oxford Brookes University	Co-author
Prof Dyfrig Hughes	Bangor University	Co-author
Janet Currier	Lay	Co-author
Deborah Roberts	Lay	Co-author

15.4 Trial Committees

15.4.1 Trial Management Group (TMG)

A TMG will be formed comprising the CI, other lead investigators (clinical and non-clinical) and members of the CTRC. The TMG will be responsible for the day-to-day running and management of the trial. The frequency of the TMG meetings will be agreed by the TMG and will depend on the demands of the different stages of the trial, for example the TMG may meet monthly in the first instance reducing to three monthly for the duration of the trial.

Refer to the TMG terms of reference and trial oversight committee membership document for further details.

15.4.2 Trial Steering Committee (TSC)

The TSC will consist of an independent chairperson with expertise in epilepsy treatment in the NHS, an independent expert in the field of paediatric clinical trials and a biostatistician. The role of the TSC is to provide overall supervision for the trial and provide advice through its independent Chairman. The ultimate decision for the continuation of the trial lies with the TSC. The TSC will meet at least annually.

Refer to the TSC terms of reference and trial oversight committee membership document for further details.

15.4.3 Independent Data and Safety Monitoring Committee (IDSMC)

The IDSMC consists of an independent chairperson, plus two independent members: one who is an expert in the field of epilepsy, and one who is an expert in medical statistics.

The IDSMC will be responsible for interim monitoring of safety and effectiveness, trial conduct and external data. The IDSMC will first convene prior to the start of recruitment and will then define frequency of subsequent meetings (at least annually). Refer to the IDSMC charter and trial oversight committee membership document for further detail. Details of the interim analysis and monitoring are provided in section 11.

The IDSMC will provide a recommendation to the TSC concerning the continuation of the trial. Refer to the IDSMC charter and trial oversight committee membership document for further details.

16 PUBLICATION AND DISSEMINATION

16.1 Publication Policy

The results from different centres will be analysed together and published as soon as possible. Individual clinicians must undertake not to submit any part of their individual data for publication or presentation. The TMG will form the basis of the publication committee and advise on all proposals to disseminate results such as in peer-reviewed publications, conference abstracts etc, as well as deal with external requests to share data or collaborate. The Publication Committee will provide feedback on such requests within 2 weeks. The Uniform Requirements for Manuscripts Submitted to Biomedical Journals (http://www.icmje.org/) will be respected. All publications shall include a list of participants, and if there are named authors, these should include the trial's Chief Investigators, Statistician(s), Health Economist(s) and Trial Manager(s) involved at least. If there are no named authors (i.e. group authorship) then a writing committee will be identified that would usually include these people, at least. The ISRCTN allocated to this trial should be attached to any publications resulting from this trial. The members of the TSC and IDSMC should be listed with their affiliations in the Acknowledgements/Appendix of the main publication. The outcomes of the qualitative component will be co-presented alongside quantitative data from the trial and separate qualitative papers may be produced. Further dissemination will also be achieved through webinars and other web-based outputs.

Authorship

Authors carry full accountability for the content of the manuscript and their reputations are reflected in the accuracy and integrity of the data. Contributors to all four of (i) the design, conduct, data analysis and interpretation, (ii) writing, (iii) manuscript approval and (iv) accountability for the integrity of the work, will be named as authors. Site PIs will be listed alphabetically in resulting publications as members of the CASTLE Consortium in the Acknowledgements section. There will be no use of hired writers.

16.2 Dissemination to Key Stakeholders

In addition to scientific outputs, we will work with charities Epilepsy Action, Epilepsy Society and Cerebra, and relevant professional groups (Royal College of Paediatrics and Child Health, Epilepsy Specialist Nurses Association) to *disseminate* the key messages in plain language summaries to families, professional groups, managers, commissioners and policy-makers.

16.3 Data Sharing

At the end of the trial, after the primary results have been published, the anonymised individual participant data (IPD) and associated documentation (e.g. protocol, SAP, annotated blank CRF) will be prepared in order to be shared with external researchers.

17 CHRONOLOGY OF PROTOCOL AMENDMENTS

17.1 Version 1.0 (20/02/2019)

Original approved protocol

17.2 Version 2.0 (24/05/2019)

Page Number	Section	Change Made
4, 5	Contact Details	Contact details for the institutions and individuals updated
13	2	Addition of a primary (economic) objective
14	2	Addition of the following secondary objective: ‘To compare parenting self-efficacy across the different treatment groups’
14	2	Rewording secondary objectives
17	3.1.2	Text added to state the embedded economic evaluation purpose
18	3.2	Text amended to clarify aspects of the economic evaluation.
19	3.3	Addition of a primary (economic) objective, outcome measure and timepoints
21	3.3	Addition of a secondary objective, outcome measure and timepoints
21	3.3	Rewording secondary objectives
25	7.2.1	Text amended to clarify that consent can only be taken by a medically qualified doctor
26	7.2.3	Text amended to clarify that consent can only be taken by a medically qualified doctor
28	7.5	Text added to clarify treatment start when participant has been selected for actigraphy.
29	7.5.2	Clarification of shipment arrangements for actigraphs
30	8.1.1	Review of seizure occurrence and hospitalisation, height and weight added to the baseline visit procedures.
30, 31	8.1.1	Order of questionnaires amended and addition of new PSAM questionnaire in the assessments
30, 31	8.1.1	The word ‘watches’ has been changed to ‘actigraphs’
30, 31	8.1.1	‘Concomitant medication CRF’ has been split into ‘AED treatment CRF’ and ‘concomitant medications related to an adverse reaction CRF’
33	Table 2	Table updated to reflect the changes in the questionnaires and their order. Height and weight added.
34	8.1.2	Text amended to clarify the completion timepoints of the ‘follow-up CRF’
35	8.2.4	Section added to include the rationale of the parent self-efficacy measure
36	8.2.5.2	Number of total dyads sent per subgroup modified to 15 but only 10 of these will be analysed.<br>Text added to explain the inclusion of sleep logs and actigraphy instructions.

		Text added to detail the steps taken from Oxford Brookes in order to send the watches and what information they will receive to do so. Shipment should occur within 1-2 days but it can vary depending on the parent/primary carer circumstances.
37	8.4.2	Text added to clarify what is PLICS data and how it will be collected.
38	8.4.3	Number of reviewed EEG's modified from 10% to all of them. They will be reviewed throughout the trial instead of at the very end.
40	9.1.1.1	Text added to state there is a window to start wearing the actigraphs
40, 41	9.1.2	Text amended to reflect the new timeline reminders for COSI (accounting for patients selected for actigraphy) and the notification methods.
43	9.2.6.1	Details regarding the collection of concomitant medications updated. These will be collected using the applicable CRF: 'AED treatment CRF' and 'concomitant medications related to the treatment of adverse reactions CRF'
46	10.2.3	Notifications of deaths will not be faxed, they will be securely emailed.
49	10.8	Fax number removed, SAE CRFs will be sent securely via email.<br>Name of the 'Delegation of Authority and Signature Log' updated.
53	11.2	Text amended to reflect the method of randomisation has been changed to minimisation.
60	13.3	Details added on the actigraphy and sleep logs data handling processes.
61	13.5	Text added to detail how identifiable data will be sent to the qualitative sub-study team.
62	13.7	Text added to clarify that raw HES data from NHS digital that will only be retained for 1 year.
63	13.7	Storage responsibilities updated to reflect it will be KHP CTO and co-sponsors (instead of CTRC) who will archive the CASTLE documentation
67	16.1	Health Economist(s) added to the list of authors.

17.3 Version 3.0 (18/08/2020)

Page Number	Section	Change Made
5	Contact Details	Contact details for the co-sponsor individual updated
34	8.1.2	Text added explaining the changes to follow up in the event of an early trial termination
38	8.4.3	Text added explaining the changes to end of trial procedures in the event of an early trial termination
41	9.2.1	Change of Carbamazepine SmPC. Removal of "Carbagen 200mg prolonged release tablets". Addition of "Tegretol 200mg prolonged release tablets".

18 REFERENCES

1. Ronen GM, Streiner DL, Rosenbaum P, Canadian Pediatric Epilepsy N. Health-related quality of life in children with epilepsy: development and validation of self-report and parent proxy measures. Epilepsia. 2003;44(4):598-612.
2. Stevens K. Valuation of the Child Health Utility 9D Index. PharmacoEconomics. 2012;30(8):729-47.
3. Dumka LE, Stoerzinger HD, Jackson KM, Roosa MW. Examination of the cross-cultural and cross-language equivalence of the parenting self-agency measure. Fam Relat. 1996;45(2):216-22.
4. Glauser T, Ben-Menachem E, Bourgeois B, Cnaan A, Guerreiro C, Kalviainen R, et al. Updated ILAE evidence review of antiepileptic drug efficacy and effectiveness as initial monotherapy for epileptic seizures and syndromes. Epilepsia. 2013;54(3):551-63.
5. Tan HJ, Singh J, Gupta R, de Goede C. Comparison of antiepileptic drugs, no treatment, or placebo for children with benign epilepsy with centro temporal spikes. The Cochrane database of systematic reviews. 2014;9:CD006779.
6. Connock M, Frew E, Evans BW, Bryan S, Cummins C, Fry-Smith A, et al. The clinical effectiveness and cost-effectiveness of newer drugs for children with epilepsy. A systematic review. Health technology assessment. 2006;10(7):iii, ix-118.
7. National Institute for Health and Care Excellence. The Diagnosis and Management of the epilepsies in adults and children in primary and secondary care/cg137. National, Institute for Health and Care Excellence; 2012 January 2012. Contract No.: cg137.
8. Mellish LC, Dunkley C, Ferrie CD, Pal DK. Antiepileptic drug treatment of Rolandic Epilepsy and Panayiotopoulos syndrome: Clinical Practice Survey and Clinical Trial Feasibility. Arch Dis Child. 2015;100:62-7.
9. Salinsky MC, Storzbach D, Spencer DC, Oken BS, Landry T, Dodrill CB. Effects of topiramate and gabapentin on cognitive abilities in healthy volunteers. Neurology. 2005;64(5):792-8.
10. Salinsky MC, Spencer DC, Oken BS, Storzbach D. Effects of oxcarbazepine and phenytoin on the EEG and cognition in healthy volunteers. Epilepsy & behavior : E&B. 2004;5(6):894-902.
11. Meador KJ, Loring DW, Vahle VJ, Ray PG, Werz MA, Fessler AJ, et al. Cognitive and behavioral effects of lamotrigine and topiramate in healthy volunteers. Neurology. 2005;64(12):2108-14.
12. Meador KJ, Loring DW, Ray PG, Murro AM, King DW, Nichols ME, et al. Differential cognitive effects of carbamazepine and gabapentin. Epilepsia. 1999;40(9):1279-85.
13. Meador KJ, Loring DW, Moore EE, Thompson WO, Nichols ME, Oberzan RE, et al. Comparative cognitive effects of phenobarbital, phenytoin, and valproate in healthy adults. Neurology. 1995;45(8):1494-9.
14. Meador KJ, Loring DW, Abney OL, Allen ME, Moore EE, Zamrini EY, et al. Effects of carbamazepine and phenytoin on EEG and memory in healthy adults. Epilepsia. 1993;34(1):153-7.
15. Meador KJ, Gevins A, Loring DW, McEvoy LK, Ray PG, Smith ME, et al. Neuropsychological and neurophysiologic effects of carbamazepine and levetiracetam. Neurology. 2007;69(22):2076-84.
16. Weijenberg A, Brouwer OF, Callenbach PM. Levetiracetam Monotherapy in Children with Epilepsy: A Systematic Review. CNS Drugs. 2015;29(5):371-82.
17. Stores G, Crawford C. Medical student education in sleep and its disorders. Journal of the Royal College of Physicians of London. 1998;32(2):149-53.
18. Stores G, Wiggs L. Sleep education in clinical psychology courses in the UK. Clinical Psychology Forum. 1998;119:14-8.
19. Wittkowski A, Garrett C, Calam R, Weisberg D. Self-Report Measures of Parental Self-Efficacy: A Systematic Review of the Current Literature. J Child Fam Stud. 2017;26(11):2960-78.
20. Whittaker KA, Cowley S. Evaluating health visitor parenting support: validating outcome measures for parental self-efficacy. J Child Health Care. 2006;10(4):296-308.
21. Owens JA, Spirito A, McGuinn M. The Children's Sleep Habits Questionnaire (CSHQ): psychometric properties of a survey instrument for school-aged children. Sleep. 2000;23(8):1043-51.
22. Cottrell L, Khan A. Impact of childhood epilepsy on maternal sleep and socioemotional functioning. Clinical pediatrics. 2005;44(7):613-6.

23. Gringras P, Gamble C, Jones AP, Wiggs L, Williamson PR, Sutcliffe A, et al. Melatonin for sleep problems in children with neurodevelopmental disorders: randomised double masked placebo controlled trial. Bmj. 2012;345:e6664.
24. Gringras P, Green D, Wright B, Rush C, Sparrowhawk M, Pratt K, et al. Weighted blankets and sleep in autistic children--a randomized controlled trial. Pediatrics. 2014;134(2):298-306.
25. Vintan MA, Palade S, Cristea A, Benga I, Muresanu DF. A neuropsychological assessment, using computerized battery tests (CANTAB), in children with benign rolandic epilepsy before AED therapy. Journal of medicine and life. 2012;5(1):114-9.
26. Bedard AC, Martinussen R, Ickowicz A, Tannock R. Methylphenidate improves visual-spatial memory in children with attention-deficit/hyperactivity disorder. Journal of the American Academy of Child and Adolescent Psychiatry. 2004;43(3):260-8.
27. Sugden C, Housden CR, Aggarwal R, Sahakian BJ, Darzi A. Effect of pharmacological enhancement on the cognitive and clinical psychomotor performance of sleep-deprived doctors: a randomized controlled trial. Annals of surgery. 2012;255(2):222-7.
28. Faridi N, Karama S, Burgaleta M, White MT, Evans AC, Fonov V, et al. Neuroanatomical correlates of behavioral rating versus performance measures of working memory in typically developing children and adolescents. Neuropsychology. 2015;29(1):82-91.
29. Wille N, Badia X, Bonsel G, Burstrom K, Cavrini G, Devlin N, et al. Development of the EQ-5D-Y: a child-friendly version of the EQ-5D. Quality of life research : an international journal of quality of life aspects of treatment, care and rehabilitation. 2010;19(6):875-86.
30. Borggraefe I, Bonfert M, Bast T, Neubauer BA, Schotten KJ, Massmann K, et al. Levetiracetam vs. sulthiame in benign epilepsy with centrotemporal spikes in childhood: a double-blinded, randomized, controlled trial (German HEAD Study). European journal of paediatric neurology : EJPN : official journal of the European Paediatric Neurology Society. 2013;17(5):507-14.
31. Larson AM, Ryther RC, Jennesson M, Geffrey AL, Bruno PL, Anagnos CJ, et al. Impact of pediatric epilepsy on sleep patterns and behaviors in children and parents. Epilepsia. 2012;53(7):1162-9.
32. Montgomery AA, Peters TJ, Little P. Design, analysis and presentation of factorial randomised controlled trials. BMC medical research methodology. 2003;3:26.
33. Schulz KF, Altman DG, Moher D. CONSORT 2010 statement: Updated guidelines for reporting parallel group randomised trials. Journal of pharmacology & pharmacotherapeutics. 2010;1(2):100-7.
34. Schulz KF, Grimes DA. Multiplicity in randomised trials I: endpoints and treatments. Lancet. 2005;365(9470):1591-5.
35. Dakin H, Gray A. Economic evaluation of factorial randomised controlled trials: challenges, methods and recommendations. Stat Med. 2017;36(18):2814-30.
36. Fenwick E, Claxton K, Sculpher M. Representing uncertainty: the role of cost-effectiveness acceptability curves. Health economics. 2001;10(8):779-87.
37. Husereau D, Drummond M, Petrou S, Carswell C, Moher D, Greenberg D, et al. Consolidated Health Economic Evaluation Reporting Standards (CHEERS) statement. Bmj. 2013;346:f1049.

**From:**	Pal, Deb
**To:**	Castle, RCT; Tudur Smith, Catrin
**Cc:**	Spowart, Catherine
**Subject:**	Re: CASTLE Protocol (V3.0, 18/08/2020) - CI/Stats Approval
**Date:**	18 August 2020 10:41:47
**Attachments:**	image001.png

Hi Nadia

In my capacity as Chief Investigator, I approve the changes to the protocol version 3.0 here. I have included my electronic signature.

Professor Deb Pal

Deb K Pal PhD MRCP
Professor of Paediatric Epilepsy
Department of Basic and Clinical Neurosciences
Institute of Psychiatry, Psychology & Neuroscience
King's College, London

mailing address:
Maurice Wohl Clinical Neuroscience Institute
5 Cutcombe Road
London SE5 9RX

Tel: +44 (020) 7848 5762
Email: deb.pal@kcl.ac.uk
URL: www.childhood-epilepsy.org

From: Castle, RCT <castle.rct@liverpool.ac.uk>
Sent: 18 August 2020 10:03
To: Pal, Deb <deb.pal@kcl.ac.uk>; Tudur Smith, Catrin <cat1@liverpool.ac.uk>
Cc: Spowart, Catherine <Catherine.Spowart@liverpool.ac.uk>
Subject: CASTLE Protocol (V3.0, 18/08/2020) - CI/Stats Approval

Hi Deb and Catrin,

Please find attached a finalised copy of the latest CASTLE Protocol (V3.0, 18/08/2020).

If you are happy, could I please ask you to provide email approval. As per our Quality Assurance processes during COVID-19, could I please ask you confirm the following in your email reply:

- In what capacity you are responding
- The document you are reviewing
- Whether you are happy with the content or any comments you have, etc

Thanks,
Nadia

Nadia Al-Najjar
CASTLE Trial Co-ordinator¦ Liverpool Clinical Trials Centre ¦ The University of

Liverpool

☎ 0151 795 8774 ¦ ✉ castle.rct@liverpool.ac.uk

Liverpool Clinical Trials Centre, University of Liverpool, 2nd Floor, Institute in the Park, Alder Hey Children's NHS Foundation Trust, Eaton Road, Liverpool, L12 2AP

The information in this email and any attachments is confidential. Unless you are the intended recipient, you must not read, copy, distribute, use or retain this message or any part of it. If you are not the intended recipient, please notify the sender immediately

**From:**	Tudur Smith, Catrin
**To:**	Castle, RCT
**Cc:**	Spowart, Catherine
**Subject:**	RE: CASTLE Protocol (V3.0, 18/08/2020) - CI/Stats Approval
**Date:**	01 September 2020 13:10:10
**Attachments:**	image002.png

Hi Nadia

I approve this

Thanks
Catrin

From: Castle, RCT <castle.rct@liverpool.ac.uk>
Sent: 21 August 2020 12:05
To: Tudur Smith, Catrin <cat1@liverpool.ac.uk>
Cc: Spowart, Catherine <cfell@liverpool.ac.uk>
Subject: FW: CASTLE Protocol (V3.0, 18/08/2020) - CI/Stats Approval

Hi Catrin,

When you get a chance, could you please review and provide approval for the attached CASTLE protocol (V3.0, 18/08/2020)?

Thanks,
Nadia

From: Castle, RCT
Sent: 18 August 2020 10:03
To: deb.pal@kcl.ac.uk <deb.pal@kcl.ac.uk>; Tudur Smith, Catrin <cat1@liverpool.ac.uk>
Cc: Spowart, Catherine <cfell@liverpool.ac.uk>
Subject: CASTLE Protocol (V3.0, 18/08/2020) - CI/Stats Approval

Hi Deb and Catrin,

Please find attached a finalised copy of the latest CASTLE Protocol (V3.0, 18/08/2020).

If you are happy, could I please ask you to provide email approval. As per our Quality Assurance processes during COVID-19, could I please ask you confirm the following in your email reply:

- In what capacity you are responding
- The document you are reviewing
- Whether you are happy with the content or any comments you have, etc

Thanks,
Nadia

Nadia Al-Najjar
CASTLE Trial Co-ordinator ¦ Liverpool Clinical Trials Centre ¦ The University of Liverpool
☎ 0151 795 8774 ¦ ✉ castle.rct@liverpool.ac.uk

Liverpool Clinical Trials Centre, University of Liverpool, 2nd Floor, Institute in the Park, Alder Hey Children's NHS Foundation Trust, Eaton Road, Liverpool, L12 2AP

The information in this email and any attachments is confidential. Unless you are the intended recipient, you must not read, copy, distribute, use or retain this message or any part of it. If you are not the intended recipient, please notify the sender immediately
