# Supplementary material for: The impact of parent treatment preference and other factors on recruitment: lessons learned from a paediatric epilepsy randomised controlled trial
Source: Trials. 2023 Feb 6;24:83. doi: 10.1186/s13063-023-07091-9 (PMC9900533; doi:10.1186/s13063-023-07091-9)
Supplement: Supplementary file 2 — Additional file 2. Top Tips for Recruiting Families. Top Tips for Recruiting Families-original CASTLE Trial. [file 13063_2023_7091_MOESM2_ESM.pdf]

Tips for clinicians recruiting children to the CASTLE trial

We have spoken to lots of families of children with epilepsy about the CASTLE trial. They gave us some tips on important things to say about how the trial is introduced and explained to families. For each of the questions they raised, we have created responses that they think will be helpful when you discuss the trial with parents/families.

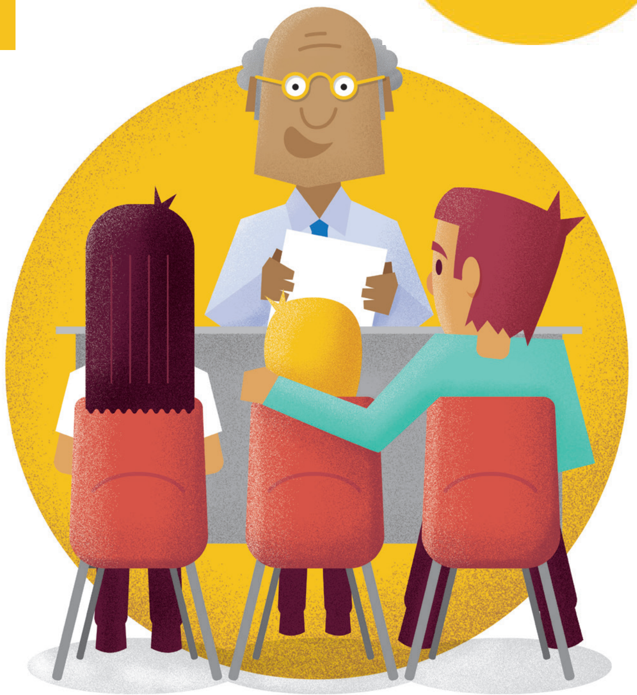

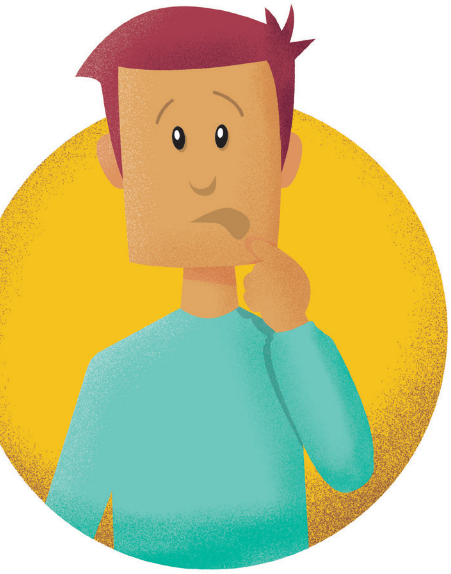

"There is a lot of information to take on board, why is the trial happening?"

"I know you've just found out that your child has epilepsy and you've been given lots of information which can feel really overwhelming. However, other parents of children with epilepsy have told us that they want to understand uncertainties about treatments and hear about new trials. I'd like to give you the opportunity to get involved in an important research study which is trying to work out which of the current treatment options delivered within the NHS is best for the type of epilepsy your son/daughter has been diagnosed with. Lots of different hospitals all over the country are involved with the CASTLE study to help us work out the best treatments for rolandic epilepsy".

"What treatment would my child be offered if we didn't take part and how would the support differ to what we would get taking part in the trial?"

"If you choose **not to take part** in the trial then your paediatrician will discuss treatment options with you and see you in outpatients in their usual way, this will differ from hospital to hospital".

"If you decided **to take part** in the trial then we would actively monitor your child's seizures, sleep and learning over 12 months and we would have regular contact with you. If you decided to take part in the trial you may also be allocated to receive a sleep intervention to help establish a good sleep routine for your child. Your child is more important than the trial, so if you changed your mind about taking part or your child's needs changed then you can opt out at any time".

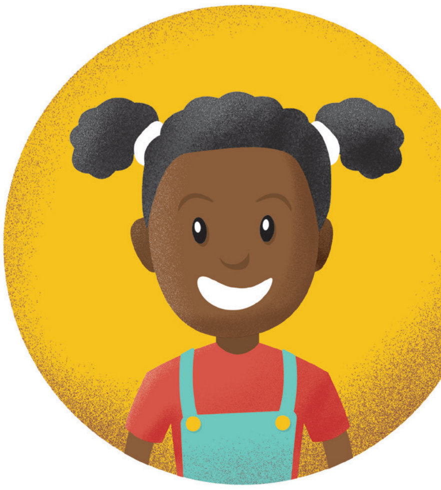

"The trial sounds really complicated, what are the different parts?"

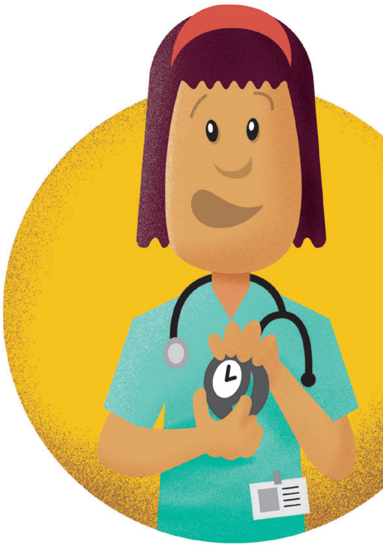

"I have a flowchart to help explain the trial and its different parts".

"We are so worried about his/her seizures, can't we just have some medicine to help stop them?"

"I totally understand that you feel there is a need to do something, but we know that anti-epileptic medicines don't always help the type of seizures your child has and medicines can have side-effects and may in some cases worsen your child's learning or sleep. There is a lot of careful extra attention being given to all the children who get involved in the CASTLE study, as we offer something called active monitoring".

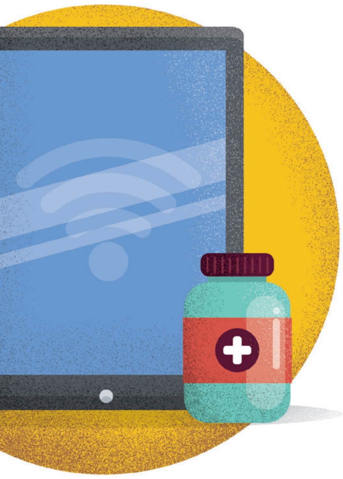

"What is active monitoring?"

"Active monitoring means we are carefully looking at every child's learning, overall wellbeing, numbers of seizures, and sleep more than we would usually do in clinic. We are doing this whether they are receiving a medicine or not - We will also test a new online sleep tool for some children".

"Why can't we decide which medicine they get on the trial?"

"In order for us to work out which treatment is best for children with this type of epilepsy, we have to assign children to the different treatments into equal groups - and not based on my decision or your decision. We do not know which treatment is better and this is the best way to find out. We need to make sure that if one group of children in the trial does better or worse than another group, then we can be sure that the reason for that difference is because of the different treatment that group received".

"What would you recommend?"

"I see many children with different types of epilepsy. Parents often ask me what treatment to choose, and I try and steer them in the right direction, but, for rolandic epilepsy, I have to stop and say, 'Actually, there isn't really any evidence that anti-epileptic medicines are better than careful follow-up without any medicines, because there have never been any proper trials. We don't know what treatment is best. My assessment of your child is that they would be suitable for any of the trial options".

"Can we decide now whether to take part or not... because we do not want to delay treatment?"

"It is important that you take time to think about what is best for you and your child. This is just as important whether you decide to take part in the trial or not. Deciding to take part in the trial will not delay starting any treatment. Of course, if you do not want to take part then that is absolutely fine".

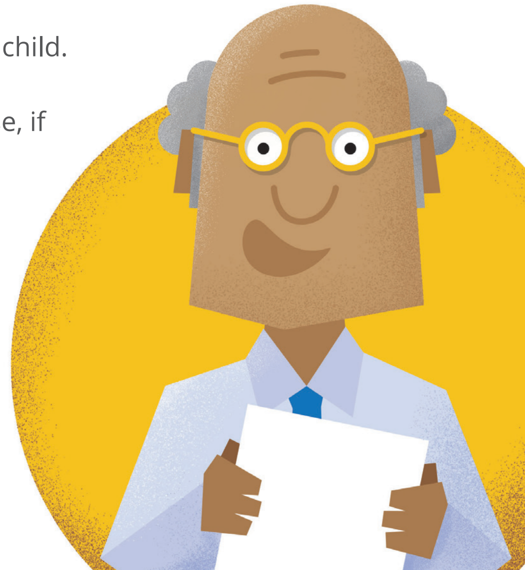

"What if we do not want to take part in the CASTLE trial?"

"It is fine if you do not want to take part in the trial, it is your choice. However, it is also important for us to understand why some families don't want to take part, would you be happy for us to pass your details to talk to a researcher about this?".
