# Supplementary material for: The impact of parent treatment preference and other factors on recruitment: lessons learned from a paediatric epilepsy randomised controlled trial
Source: Trials. 2023 Feb 6;24:83. doi: 10.1186/s13063-023-07091-9 (PMC9900533; doi:10.1186/s13063-023-07091-9)

Children with rolandic epilepsy

Aged 5–12 years old

CHANGING AGENDAS ON SLEEP, TREATMENT & LEARNING IN EPILEPSY
CASTLE

330 CHILDREN

Interview with 10 parents and children who chose not to take part

Randomly allocated to receive

110 CHILDREN CARBAMAZEPINE

Each group will will do learning ability games

THEN

Randomly allocated to receive

55 CHILDREN SLEEP INTERVENTION

55 CHILDREN NO INTERVENTION

08:35

08:35

Interview with 10 parents and children

110 CHILDREN LEVETIRACETAM

Each group will will do learning ability games

THEN

Randomly allocated to receive

55 CHILDREN SLEEP INTERVENTION

55 CHILDREN NO INTERVENTION

08:35

08:35

Interview with 10 parents and children

110 CHILDREN NO MEDICINE

Each group will will do learning ability games

THEN

Randomly allocated to receive

55 CHILDREN SLEEP INTERVENTION

55 CHILDREN NO INTERVENTION

08:35

08:35

Interview with 10 parents and children

Children not on the CASTLE trial

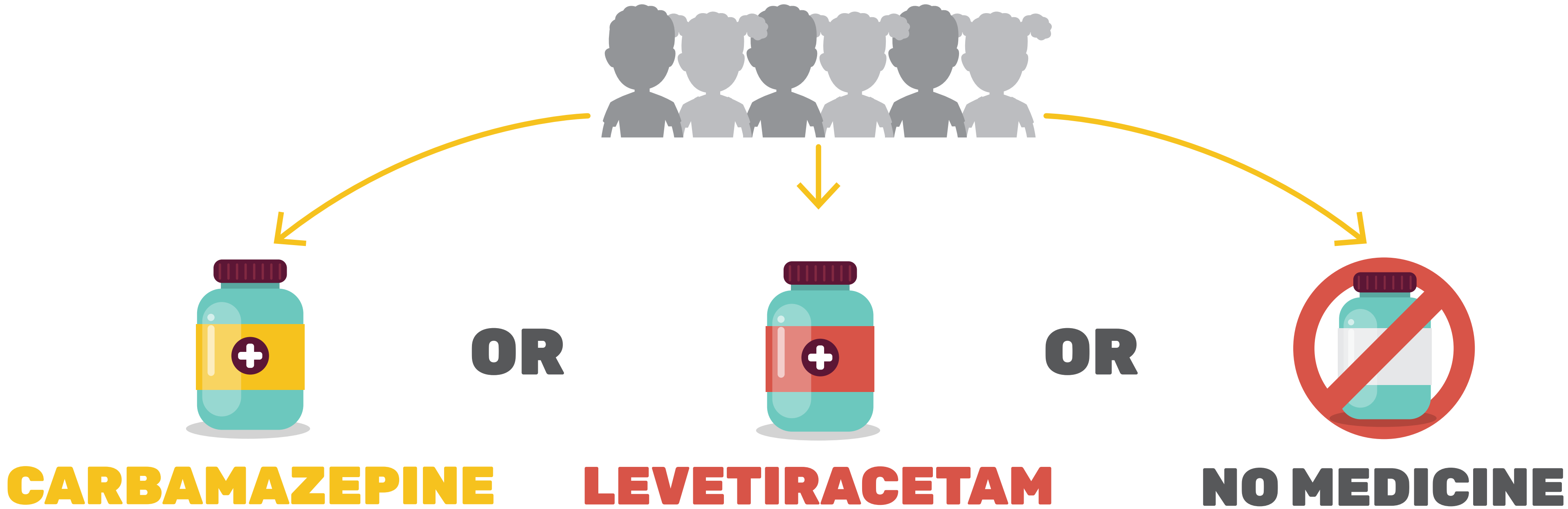

Supplement: Supplementary file 3 — Additional file 3. Pictorial Trial Flow Chart. Pictorial Trial Flow Chart-original CASTLE trial. [file 13063_2023_7091_MOESM3_ESM.pdf]
