# Supplementary material for: The impact of parent treatment preference and other factors on recruitment: lessons learned from a paediatric epilepsy randomised controlled trial
Source: Trials. 2023 Feb 6;24:83. doi: 10.1186/s13063-023-07091-9 (PMC9900533; doi:10.1186/s13063-023-07091-9)
Supplement: Supplementary file 4 — Additional file 4. Information Sheet 5-6yrs. Information Sheet for Children 5-6yrs-revised trial. [file 13063_2023_7091_MOESM4_ESM.pdf]

CASTLE SLEEP-E

King's College London

King's College Hospital NHS Foundation Trust

NHS
Trust/Site Address 1
Trust/Site Address 1 | Postcode
Telephone Number: 00000 000 000

Information Sheet for CASTLE Sleep-E Study (Child: 5 or 6 years old)

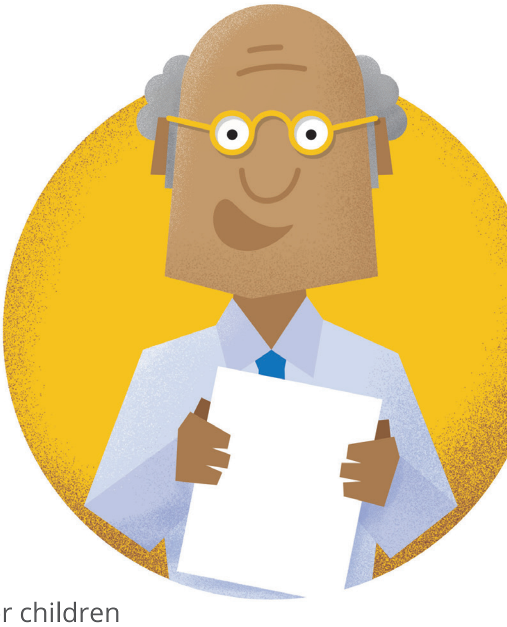

What is a study?

A study is what you do when you want to learn about something or find out something new. This study is called CASTLE Sleep-E.

Why is this study being done?

We are doing this study to find out if getting more sleep is a useful treatment for children with epilepsy. We hope our study will help children who have epilepsy.

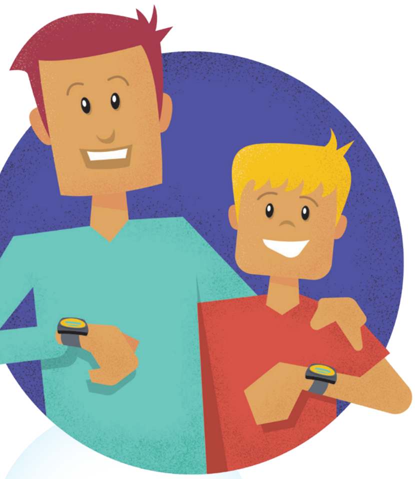

What will happen to me if I join in?

If you join in the study, your mum or dad or carer might be asked to try some things to help you sleep better.

The study lasts 6 months and you can help us three times.

You will be asked to wear a special watch that checks how well you are sleeping. Your mum or dad or carer will wear a special watch as well.

We will ask you to play a game and answer some questions on a phone or an iPad or a computer.

If you want to take part, tell your mum or dad or carer.

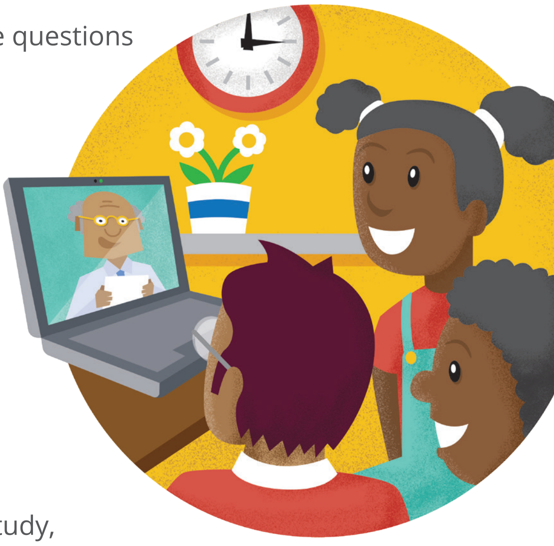

Do I have to join in the study?

No – you don't.

If you don't want to join in nobody will mind.

If you change your mind, that's okay as well.

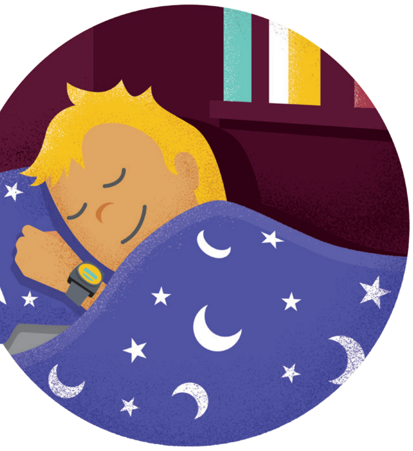

What if I feel upset about the study?

If you feel upset about anything to do with the study, tell your mum or dad or carer.

What if I have got some questions about the study?

Your mum or dad or carer have been given lots of information and they can probably help.

If you still have some questions, they can talk to one of the nurses or doctors doing the study.

Thank you for reading about this study.
If you have any questions, please do ask.

CASTLE Sleep-E Child (5-6yrs) Information Sheet, V2.0 01/10/2021 IRAS: 289580
