# Supplementary material for: The impact of parent treatment preference and other factors on recruitment: lessons learned from a paediatric epilepsy randomised controlled trial
Source: Trials. 2023 Feb 6;24:83. doi: 10.1186/s13063-023-07091-9 (PMC9900533; doi:10.1186/s13063-023-07091-9)
Supplement: Supplementary file 5 — Additional file 5. Information Sheet 7-12yrs. Information Sheet etc for Children 7-12yrs-revised trial. [file 13063_2023_7091_MOESM5_ESM.pdf]

Information Sheet for CASTLE Sleep-E Study
(Child: 7–12 years old)

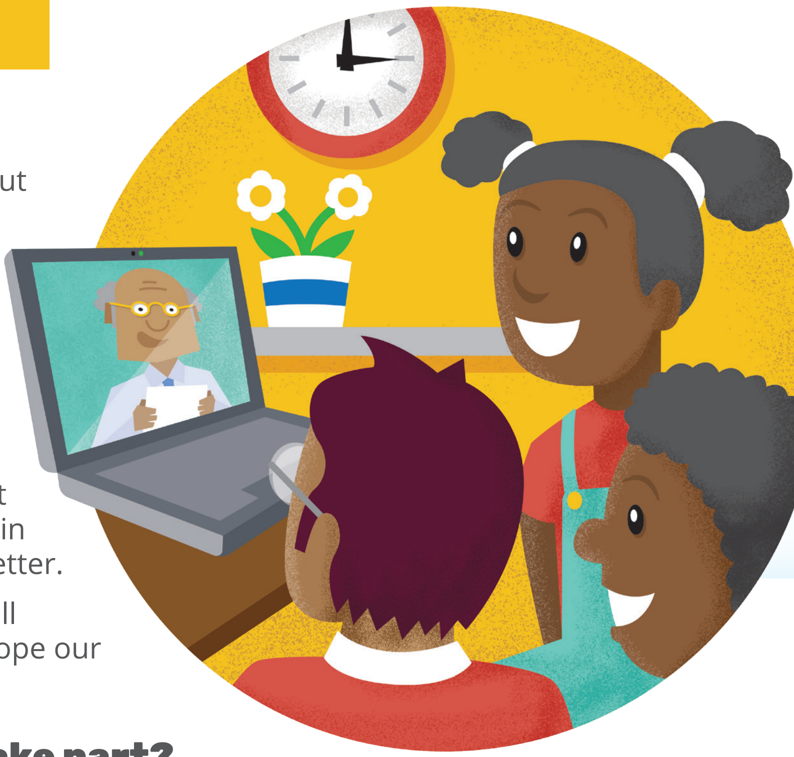

What is a study?

A study is what you do when you want to learn about something or find out something new. This study is called CASTLE Sleep-E.

What is this study all about?

We are doing this study to find out if getting more sleep is a useful treatment for children with epilepsy.

We think that helping children to sleep better might help their epilepsy. Half the children who take part in the study will try some things to help them sleep better.

We hope that 110 children (aged 5-12 years) from all across the country will take part in our study. We hope our study will help children who have epilepsy.

What will happen to me if I want to take part?

If you take part in the study, your mum or dad or carer might be asked to try some things to help you to sleep better.

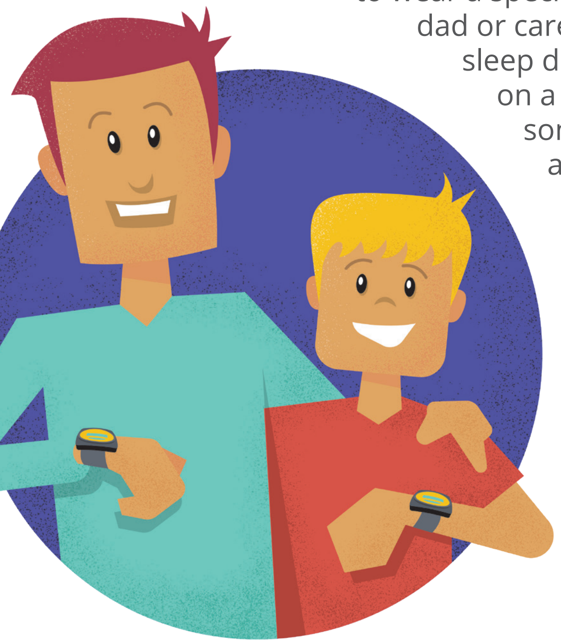

The study lasts 6 months and we would like you to help us three times. You will be asked to wear a special watch that checks how well you are sleeping. Your mum or dad or carer will wear a special watch as well. We hope you will help fill in a sleep diary. We will ask you to play a game and answer some questions on a phone or an iPad or a computer. Your parents will also be asked some questions. Some questions will be about your epilepsy and your seizures and some will be about how your epilepsy affects you. We will also check to see if you've been in hospital and what this was for.

We will also ask some children to fill in a booklet and take part in an interview (a conversation) with one of the people doing the study.

If you want to take part, tell your mum or dad or carer. We will ask you to write your name on a phone or an iPad or a computer, this tells us you understand the study and what will happen. Your parents will also have to sign a form to say they are happy for you to take part.

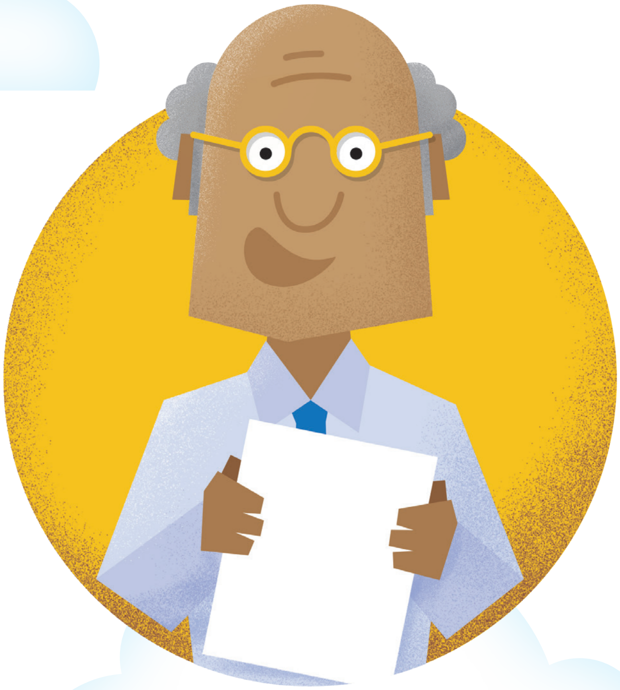

Do I have to take part in the study?

No – you don't. If you don't want to join in nobody will mind. If you change your mind, that's okay as well.

What if I feel upset about the study?

If you feel upset about anything to do with the study, tell your mum or dad or carer.

What are the good or bad things about taking part?

We can't promise that taking part will help your epilepsy, but we don't think that there are any bad things about taking part. We hope taking part won't use too much of your time.

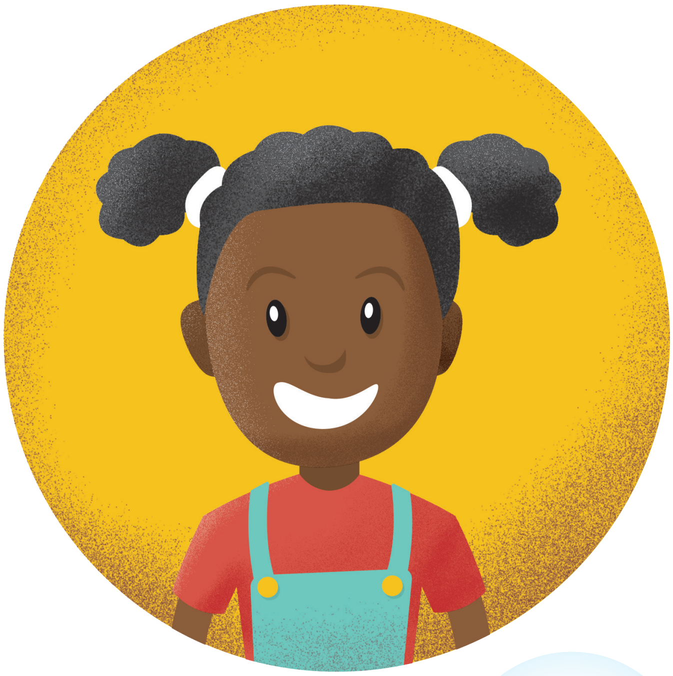

Who can I ask about this?

Your mum or dad or carer have been given lots of information, but you can also talk to a nurse or doctor who is doing the study. <Insert local nurse name> AND <number>

Who is doing the study?

The study is being run in your hospital. It is organised by King's College Hospital NHS Foundation Trust, King's College London and the University of Liverpool.

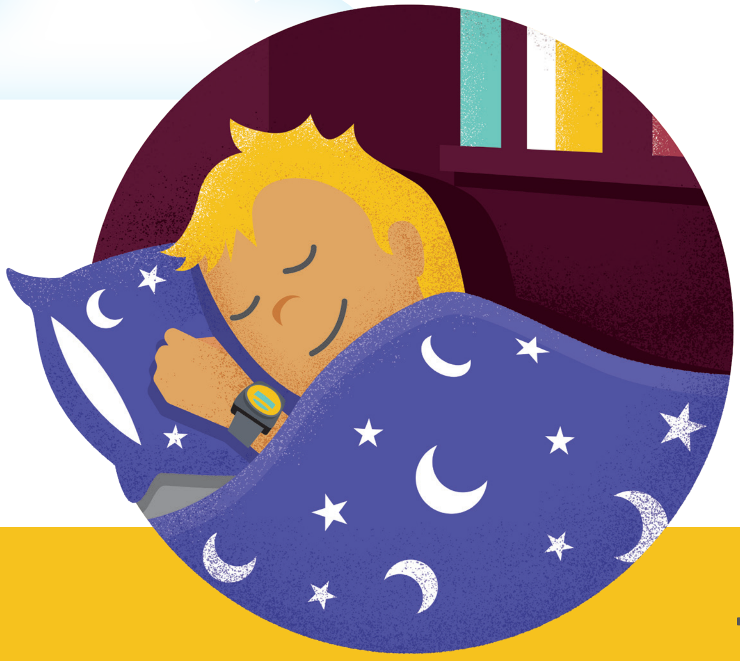

Thank you for reading about this study.
If you have any questions, please do ask.

Trust/Site Address 1
Trust/Site Address 1 | Postcode
Telephone Number: 00000 000 000

CASTLE SLEEP-E

King's College LONDON

NHS King's College Hospital NHS Foundation Trust

NHS

To be completed by the Researcher:

Site Name:

Participant Study Number

Participant Initials

Participant DOB:

Assent Form CASTLE Sleep-E Study (Child: 7-12 years old)

To be completed by the Researcher:

**Section 1 – Assessing Developmental Capacity**		
**Does the child / young person have the developmental capability / mental capacity to consider assent?** If "YES", you should approach the child / young person for assent - proceed to section 2. If "NO", you should not approach the child for assent – skip section 2, sign and date section 3 and return a copy to LCTC	Yes ☐	No ☐
**Section 2 – Approaching for Assent**		
**If approached for assent, did the child / young person express to you that they did NOT wish to make a decision about assent?** If "YES", you should not approach the child / young person for assent – sign and date section 3 and return a copy to LCTC If "NO", please sign and date section 3, then give this form to the child / young person and the adult consenting on their behalf for completion.	Yes ☐	No ☐

**Section 3 – Signature**	
Name and Role:	Signature and Date:

Child / Young person to select all they agree with:

**Have you read (or had read to you) information about this study?**	Yes	No
**Has somebody else explained this study to you?**	Yes	No
**Do you understand what this study is about?**	Yes	No
**Have you asked the questions you want?**	Yes	No
**Did you understand the answers to your questions?**	Yes	No
**Do you understand it's OK to stop taking part at any time?**	Yes	No
**Are you happy to be part of this study?**	Yes	No
**Do you understand that the person looking after you has agreed to you taking part in this study?**	Yes	No

CASTLE SLEEP-E

KING'S College LONDON

NHS King's College Hospital NHS Foundation Trust

NHS

Trust/Site Address 1 | Trust/Site Address 1 | Postcode | Telephone Number: 00000 000 000

To be completed by the Researcher:

Site Name: ______________________

Participant Study Number ______________________

Participant Initials ______________________

Participant DOB: ______________________

Assent Form CASTLE Sleep-E Study (Child: 7-12 years old)

If any answers are "no" or you do not want to take part, please leave this section blank.

If you do want to take part, please write your name and today's date.

Your Name: ______________________

Date: ______________________

Your Parent/Guardian must write their name here to if they are happy for you to take part in the study.

Name of Parent/Guardian: ______________________

Signature: ______________________

Date: ______________________

The researcher who explained this study needs to sign too:

Name of Researcher: ______________________

Signature: ______________________

Date: ______________________

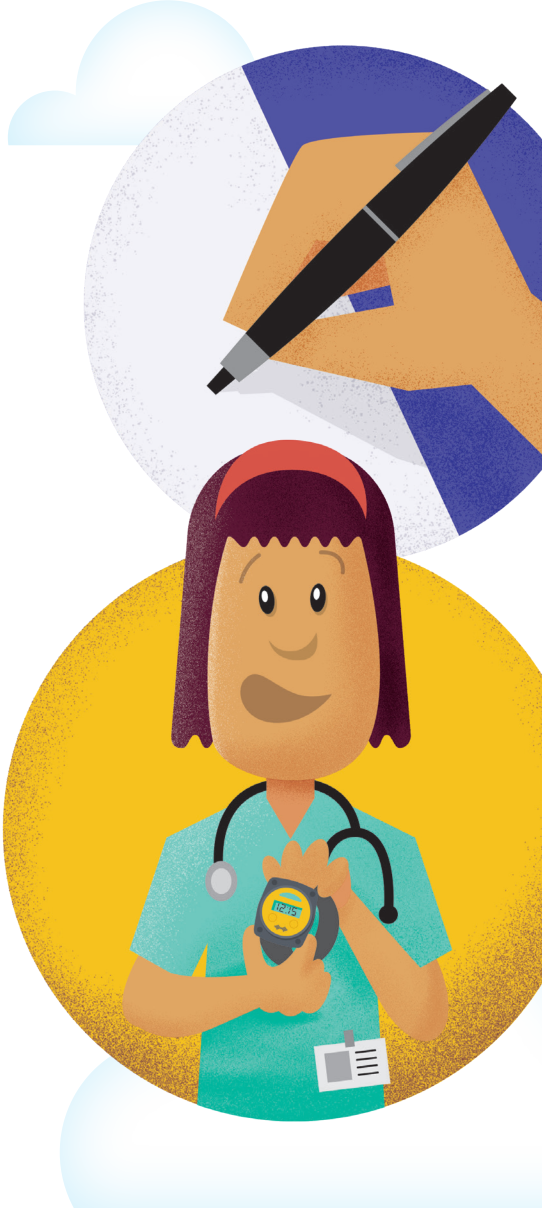

Thank you for reading about this study.
If you have any questions, please do ask.
